# Supplementary material for: CANDiT: A machine learning framework for differentiation therapy in colorectal cancer
Source: Cell Rep Med. 2025 Oct 20;6(11):102421. doi: 10.1016/j.xcrm.2025.102421 (PMC12711679; doi:10.1016/j.xcrm.2025.102421)
Supplement: Document S1. Figures S1–S8 and Tables S1, S3, and S7 [file mmc1.pdf]

Cell Reports Medicine, Volume *6*

Supplemental information

CANDiT: A machine learning framework

for differentiation therapy in colorectal cancer

Saptarshi Sinha, Joshua Alcantara, Kevin Perry, Vanessa Castillo, Annelies K. Ondersma, Satarupa Banerjee, Ella McLaren, Celia R. Espinoza, Sahar Taheri, Eleadah Vidales, Courtney Tindle, Adel Adel, Siamak Amirfakhri, Joseph R. Sawires, Jerry Yang, Michael Bouvet, and Pradipta Ghosh

SUPPLEMENTARY FIGURES AND LEGENDS

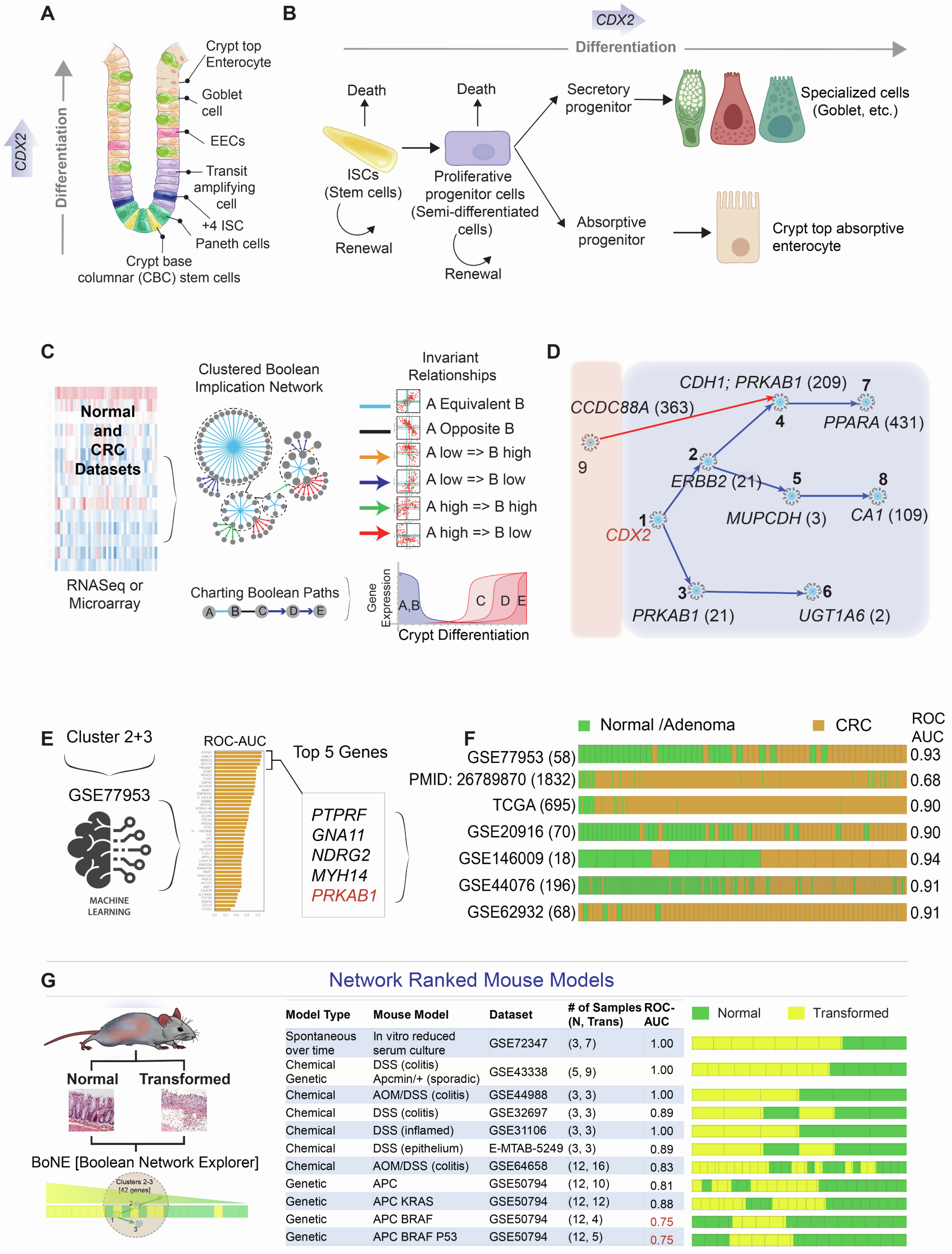

Model Type	Mouse Model	Dataset	# of Samples (N, Trans)	ROC-AUC
Spontaneous over time	In vitro reduced serum culture	GSE72347	(3, 7)	1.00
Chemical Genetic	DSS (colitis) Apcmin/+ (sporadic)	GSE43338	(5, 9)	1.00
Chemical	AOM/DSS (colitis)	GSE44988	(3, 3)	1.00
Chemical	DSS (colitis)	GSE32697	(3, 3)	0.89
Chemical	DSS (inflamed)	GSE31106	(3, 3)	1.00
Chemical	DSS (epithelium)	E-MTAB-5249	(3, 3)	0.89
Chemical	AOM/DSS (colitis)	GSE64658	(12, 16)	0.83
Genetic	APC	GSE50794	(12, 10)	0.81
Genetic	APC KRAS	GSE50794	(12, 12)	0.88
Genetic	APC BRAF	GSE50794	(12, 4)	0.75
Genetic	APC BRAF P53	GSE50794	(12, 5)	0.75

Figure S1 [Related to Figure 1].

Boolean Network-Based Identification of CDX2-Linked Differentiation Targets. A. Schematic of epithelial differentiation in the colonic crypt, where stem cells reside at the crypt base, and fully differentiated goblet cells and enterocytes localize toward the top. **B**.

Illustration of the continuum of crypt differentiation, highlighting various progenitor cell states along the axis from stemness to terminal differentiation. **C**. Overview of the Clustered Boolean Implication Network (BoNE) workflow. Diverse transcriptomic datasets are integrated to generate clusters of co-expressed genes, which are interconnected through one of six invariant Boolean relationships. **D**. Boolean network centered on *CDX2* as a seed gene, generated using the computational platform, Boolean Network Explorer (BoNE[30]), using a pooled microarray dataset (1662 CRCs, 68 adenomas, 170 normal colon tissues). Directed edges indicate Boolean relationships. **E-F**. Machine learning-driven target prioritization. Boolean clusters directly connected to *CDX2* (clusters #2 and #3) were evaluated by linear regression on test datasets. Top five genes (E) from each cluster were selected based on their ability to distinguish CRC from normal tissue (F: GSE dataset identifiers, bar plots, and ROC-AUC values shown). **G**. ROC-AUC analysis showing the ability of Boolean clusters #2 and #3 (which include *PRKAB1* and *CDX2*) to distinguish normal from transformed (and/or inflamed colitic) states in murine colitis models based on gene expression patterns.

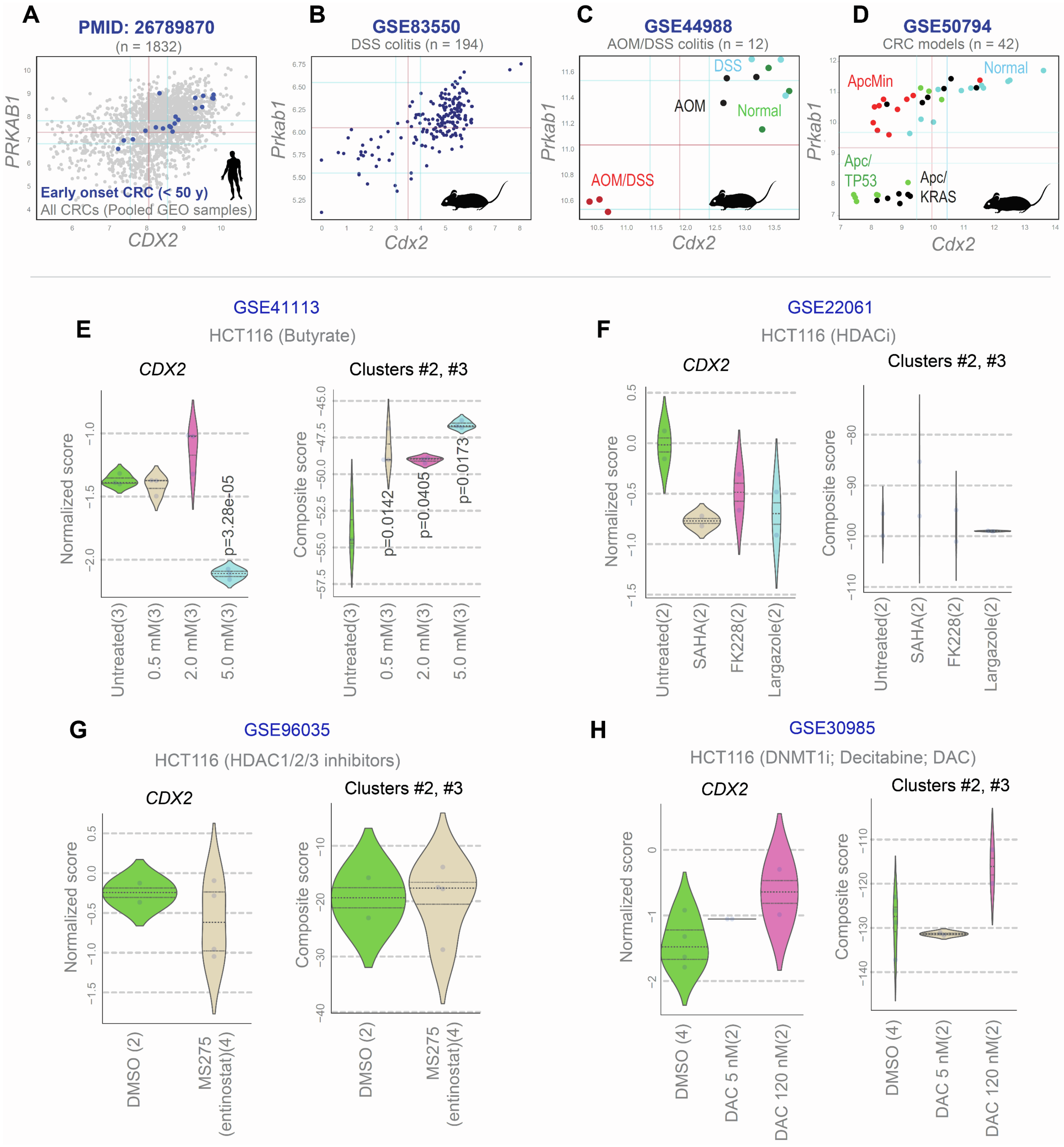

Figure S2 [Related to Figure 1].

PRKAB1 Exhibits a Conserved Universal Relationship with and Predicted to Restore CDX2 when Prior CDX2-Restorative Therapeutics have Failed. A-D. Scatter plots demonstrating a universal equivalent Boolean relationship between *PRKAB1* and *CDX2* across multiple pooled transcriptomic datasets from human and murine models of colorectal cancer (CRC) and colitis. **E-H**. Violin plots show the expression pattern of *CDX2* (left) and network-derived gene clusters #2 and #3 (right) as an effect of various treatments in HCT116 cell lines. *Statistics*: p-values were calculated by Welch's t-test (compared to untreated or control sample#1 in each plot). Only significant p-values are shown.

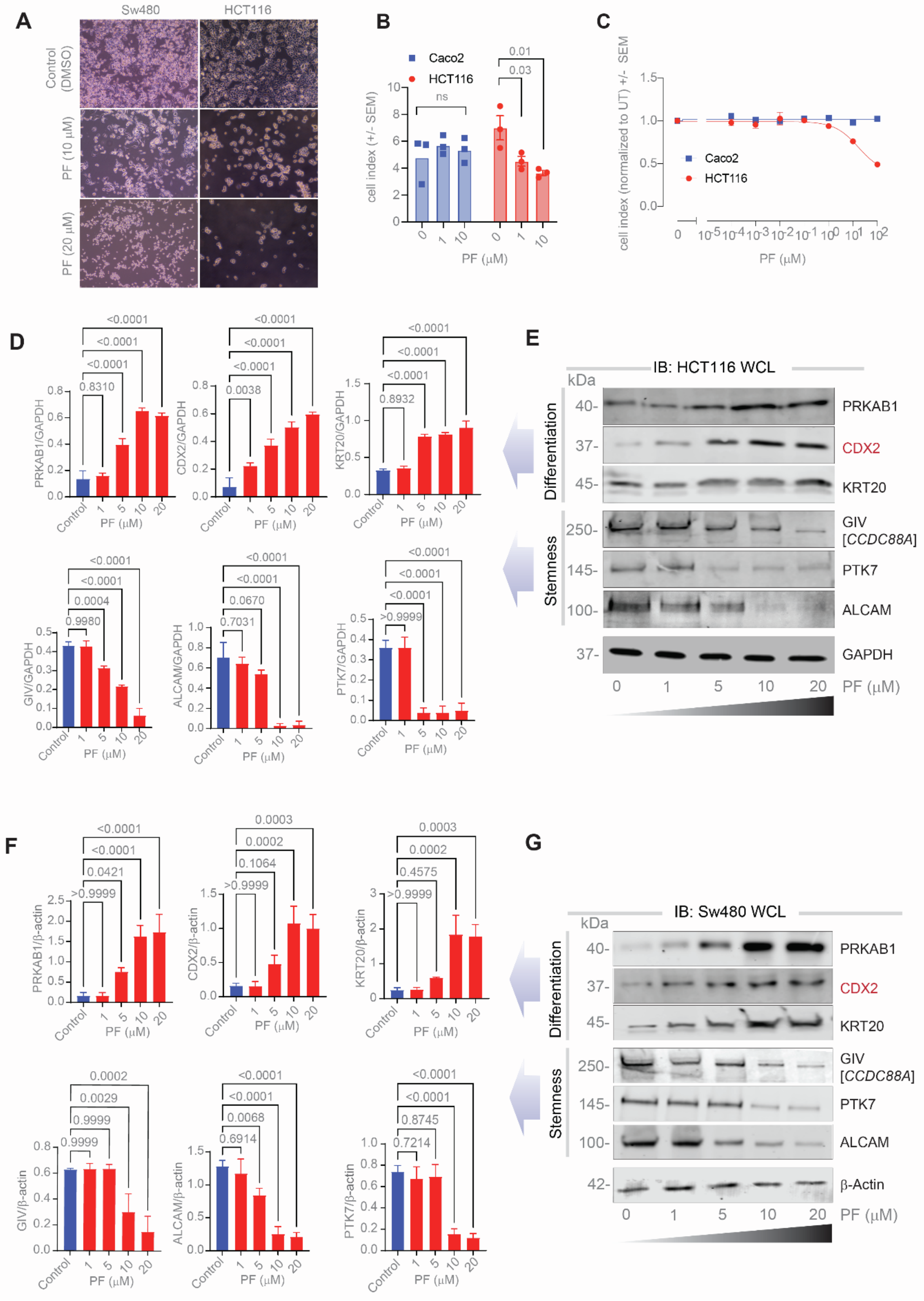

Figure S3 [Related to Figure 2].

PRKAB1 Agonist (PF) Induces Differentiation in CRC Cell Lines. A. Bright-field images (10×) of CRC cell lines treated with 10 μM and 20 μM PF compound for 16 hours. **B-C**. Bar plot (B) showing cell index, measured as impedance, for CDX2-high Caco2 cells and CDX2-low HCT116 cells after 128 h of incubation with varying concentrations of PF compound. Line plot showing normalized cell index (relative to untreated control) during 128 h of incubation with varying concentrations of PF compound. **D-G**. Immunoblots and quantification of HCT116 WCLs (D-E) and Sw480 WCLs (F-G) treated with increasing PF doses for 16 hours prior to lysis (when there is <1-2% cell death). *Statistics*: p-values were calculated using one-way ANOVA. Error bars represent S.E.M. from three independent biological replicates.

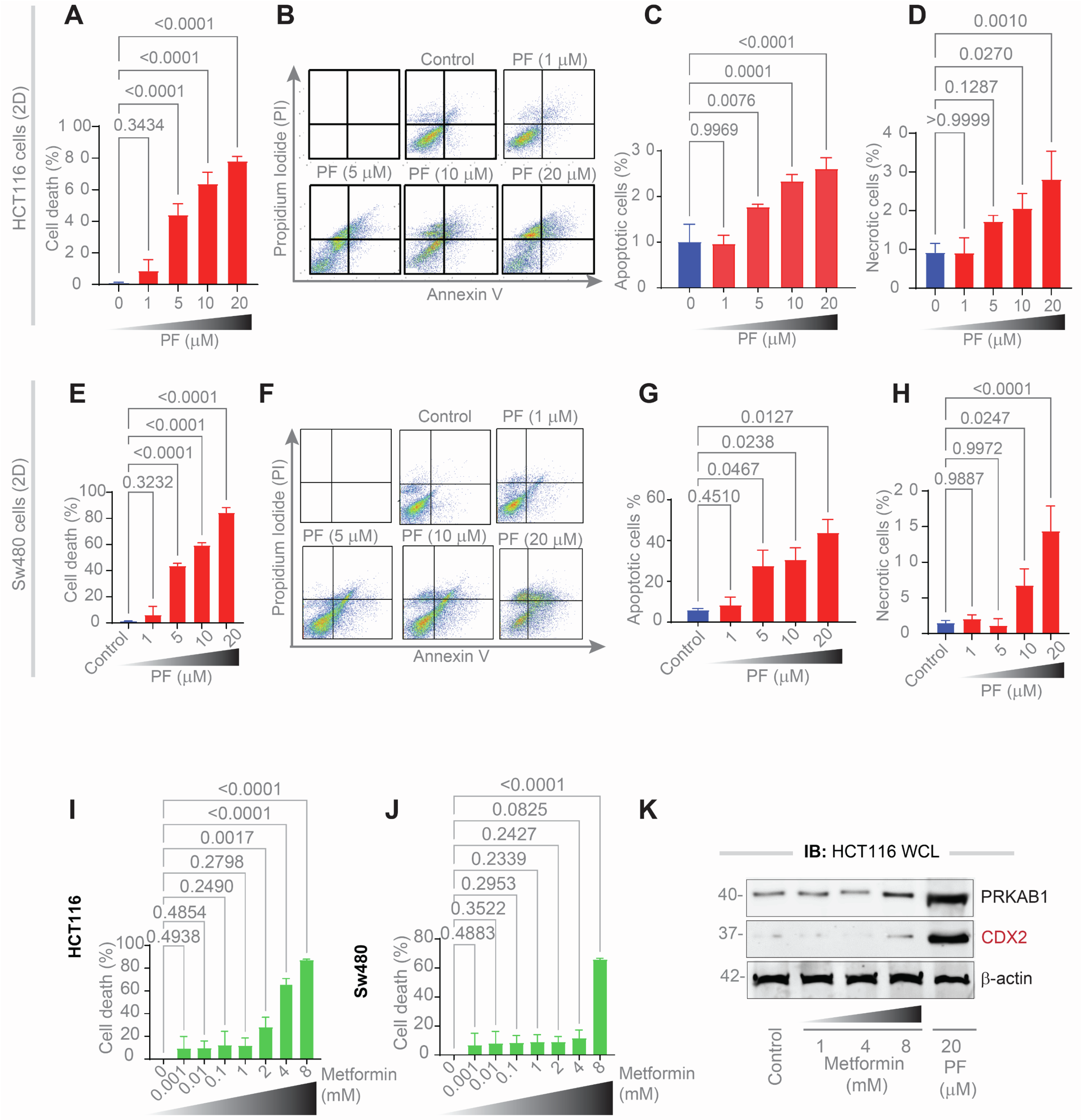

Figure S4 [Related to Figure 2].

β1-AMPK Specific Agonist (PRKAB1-agonist, PF), but Not the Indirect AMPK Activator Metformin, Induces Dose-Dependent Cell Death. A-H. Monolayers of HCT116 (A-D) and Sw480 (E-H) cells were treated with increasing concentrations of the PRKAB1 agonist PF and assessed for apoptosis using Annexin V staining followed by flow cytometry. Representative scatter plots from flow cytometry are shown in B and F. **I-J**. Monolayers of HCT116 (I) and Sw480 (J) cells were treated with various concentrations of the indirect AMPK activator Metformin, and cell viability was measured via MTT assay. **K**. Immunoblots of equal aliquots of whole cell lysates (WCL) of HCT116 cells treated with increasing doses of Metformin and 20 μM PF for 16 h prior to lysis (when there is virtually no cell death). β-actin was used as loading control. *Statistics*: p-values were calculated by one-way ANOVA. Error bars indicate S.E.M of 3 independent biological replicates.

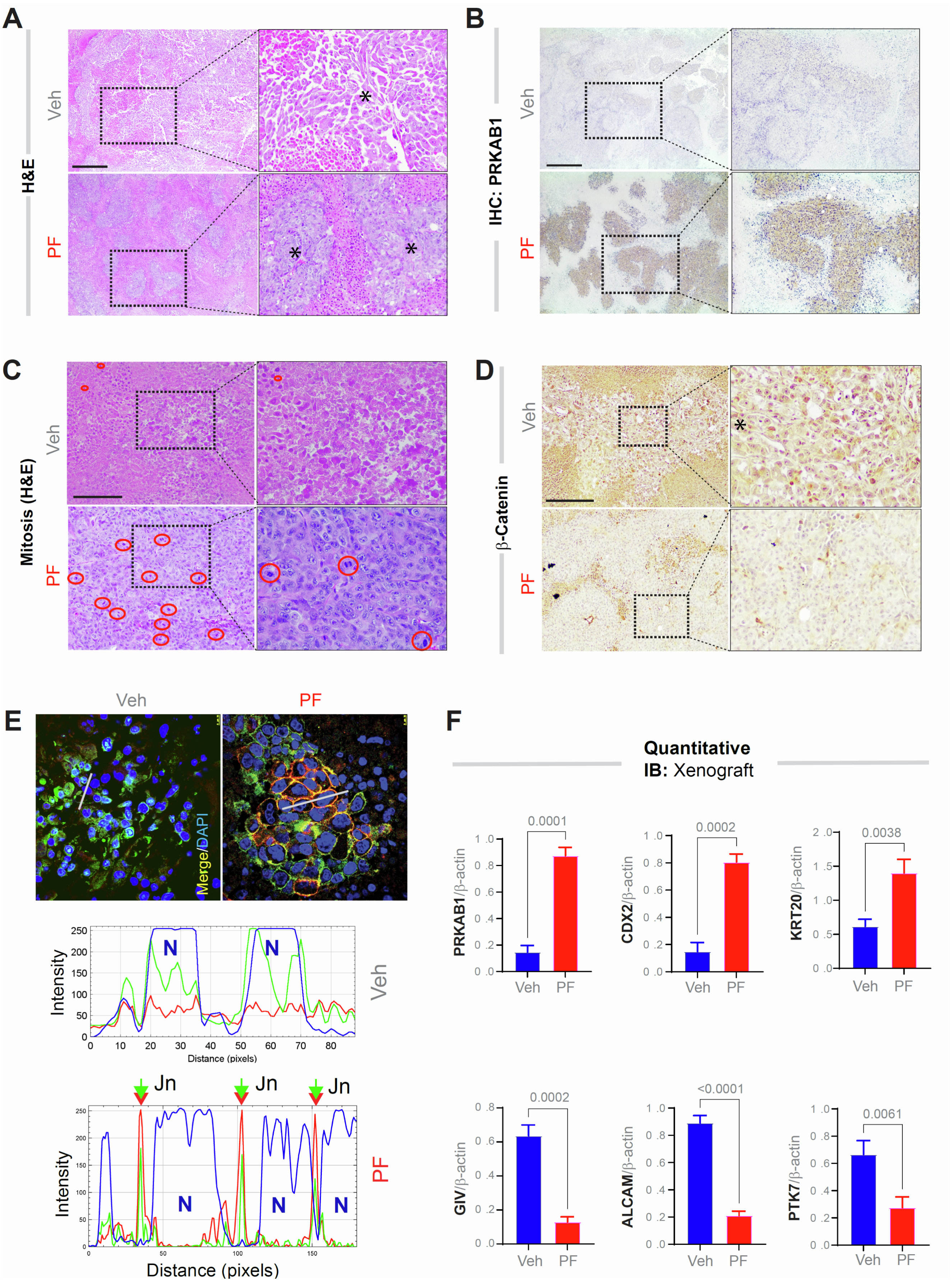

Figure S5 [Related to Figure 2].

PRKAB1-agonist (PF) Promotes Differentiation in Xenotransplants. A. Representative H&E-stained sections of FFPE tumor xenografts. Vehicle (Veh) treated tumors exhibit poorly differentiated, rhomboid-shaped cells arranged in sheet-like patterns with

minimal extracellular matrix. In contrast, PF-treated tumors display focal regions of glandular architecture with prominent cell-cell contacts. Asterisks (*) denote viable tumor cell islands; red circles in panel C highlight mitotic figures. **B-D**. Representative fields of H&E and IHC-stained FFPE tumor sections. Scale Bars = 100 µm. **E**. Representative merged images (top) of xenograft sections co-stained for β-catenin (green), E-cadherin (red), and DAPI (blue). RGB intensity plots (bottom, generated in ImageJ) demonstrate co-localization of β-catenin and E-cadherin at cell-cell junctions, indicating membrane-localized β-catenin specifically in PF-treated tumors. Scale bar = 10 µm. See individual channels in **Figure 2N**. **F**. Quantification of differentiation and stemness markers from immunoblots of xenograft lysates in **Figure 2O**. *Statistics*: p-values were calculated by paired t-test. Error bars indicate S.E.M of 3 independent biological replicates.

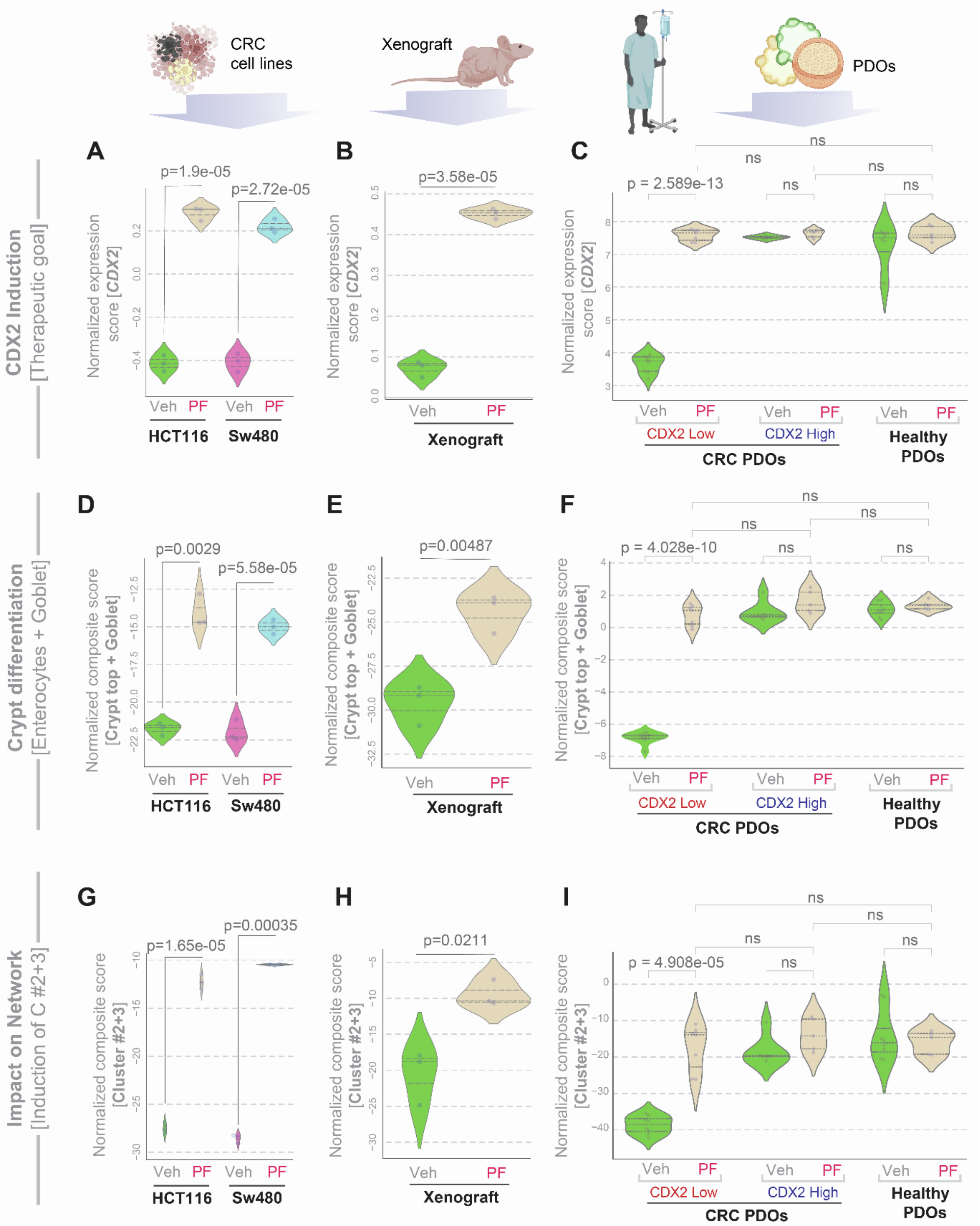

Figure S6 [Related to Figure 4].

PF treatment induces *CDX2*, markers of crypt differentiation, and genes in cluster #2 + #3 in the CRC network in CRC cell lines, xenografts, and PDOs. Violin plots display the normalized expression of *CDX2* (A-C), markers of absorptive enterocytes and Goblet cells indicative of crypt differentiation (D-F), and composite score of genes in clusters #2 and #3 from proposed CRC network (G-I) in CRC cell lines (A, D, G), tumor xenografts (B, E, H) and PDOs (C, F, I). See **Supplemental Table 2** for a compete catalogue of genes in clusters #2 and #3 and the markers of crypt differentiation. *Statistics*: p-values were calculated by Welch's t-test.

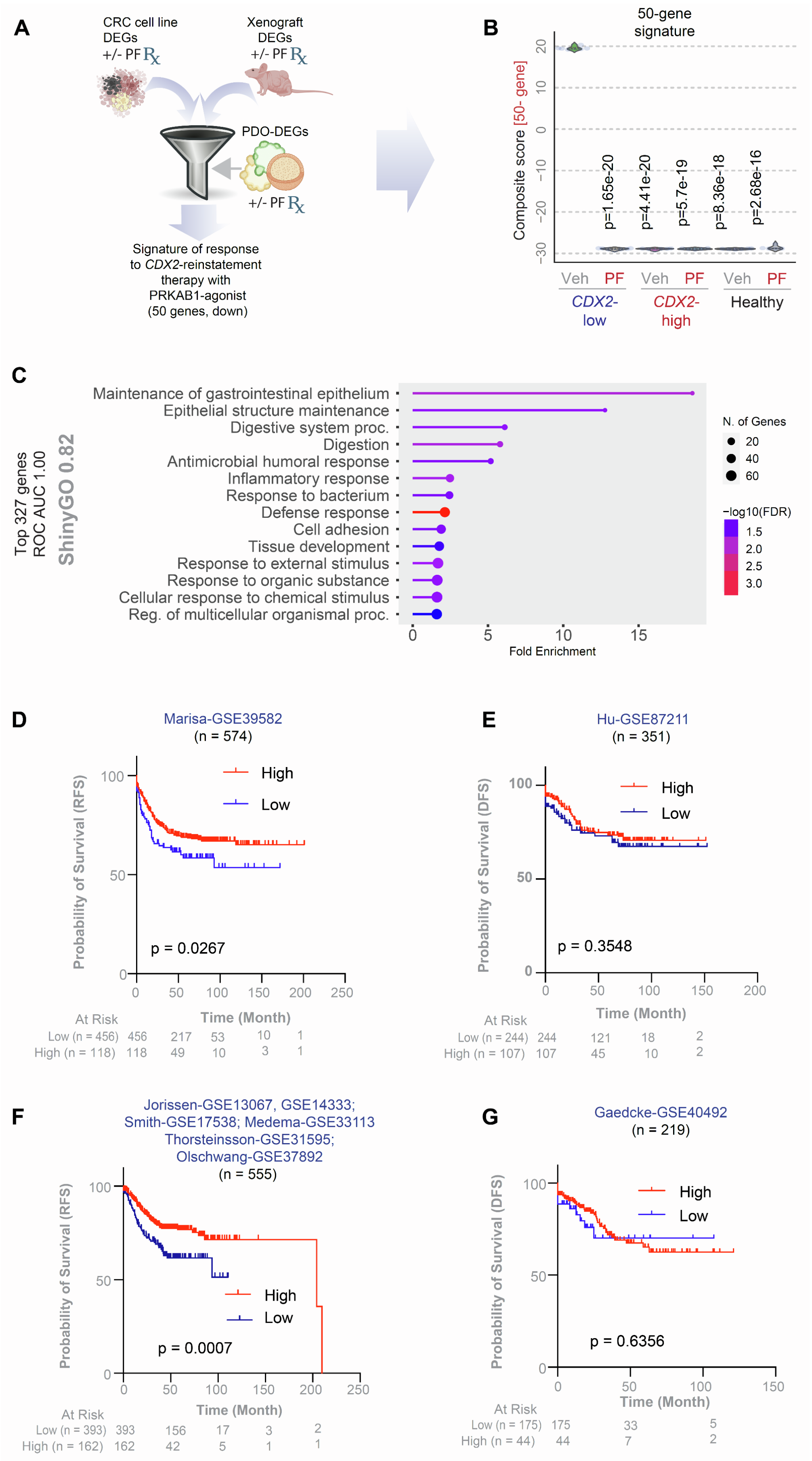

Figure S7 [Related to Figure 5].
A gene signature of therapeutic response predicts better survival in patients.

A. Computational workflow used in the derivation of a 50-gene signature of therapeutic response using an integrated DEA approach. Differentially expressed genes (DEGs) from CRC cell lines and xenografts (see **Supplemental Table 4-5**) were further refined based on their ability to accurately classify PDO samples with/without PF treatment (see **Supplemental Table 6** for gene list rank-ordered by ROC-AUC score). **B.** Violin plots show composite scores of the 50-gene signature in PDOs. p-values are based on Welch's t-test compared to vehicle (Veh)-treated *CDX2*-low PDOs. **C**. Lollipop plots depict pathway enrichment for the top 327 differentially expressed genes (DEGs) induced upon PF treatment in CRC cell lines and xenografts, which also achieved 100% classification accuracy (i.e., ROC AUC 1.00; **Supplemental Table 6**) in PDO samples. **D-G.** Kaplan-Meier plots showing probability of survival (DFS/RFS) over time in various cohort, segregated by high vs low expression scores of *CDX2*, computed using the *StepMiner* algorithm[82] within each cohort. p-value was determined by Log Rank test. See **Supplemental Table 7** for the corresponding O.R.

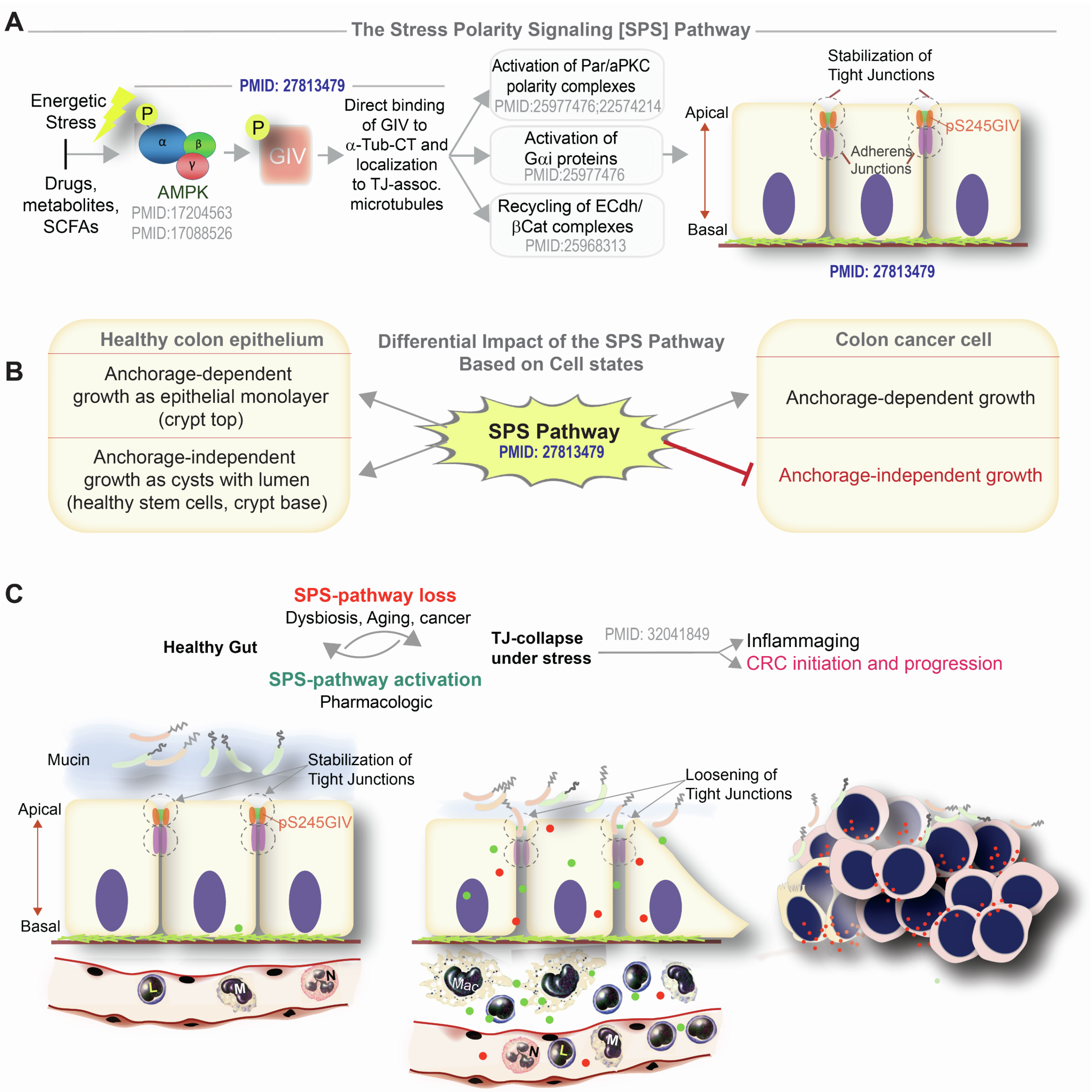

Figure S8 [Related to Figure 6].

The Stress Polarity Signaling (SPS) Pathway Maintains Epithelial Polarity in Health and Restrains Malignant Transformation.

A. Schematic overview of the SPS pathway[78-80]. Activation of a junction-localized β1-specific pool of trimeric αβγ-AMPK leads to phosphorylation of GIV at a Serine in position 245 (pSer[245]), which in turn activates Par/aPKC complexes and Gαi proteins. This cascade promotes the recycling of E-cadherin/β-catenin complexes, thereby stabilizing tight junctions and reinforcing apical polarity in epithelial cells. Corresponding PMID#s are listed.

B. Diagram illustrating previously published[6] work on the cell state-dependent cell fates induced by the SPS pathway. In healthy epithelial cells, SPS pathway activation permits anchorage-independent cystic growth with preserved lumens and anchorage-dependent growth as monolayers with enhanced barrier function. By contrast, activation of the SPS pathway in SPS-deficient transformed CRC cells is permissive only to anchorage-dependent growth as polarized monolayer but virtually abolishes growth in anchorage independent mode.

C. Schematic summarizes published work on the status of the SPS pathway in health and disease. In healthy intestinal epithelium, physiological or drug-induced SPS activation enhances apico-basal polarity and tight junction integrity via GIV phosphorylation. In pathological states (e.g., dysbiosis, aging, or cancer), SPS pathway is suppressed or lost, leading to tight junction breakdown, compromised barrier function, and increased susceptibility to inflammation and CRC progression

SUPPLEMENTARY TABLES

Supplemental Table 1 [Related to Figure 1 and S1]: A catalogue of all datasets used in model training and validation.

Model Training					
**Dataset Identifier**	**Species**	**No. of samples used**	**Normal colon**	**Cancer colon**	**Adenomas**
GSE2109	Human	393	0	393	0
GSE14333	Human	225	0	225	0
GSE26682	Human	175	0	175	0
GSE13294	Human	155	0	155	0
GSE37892	Human	129	0	129	0
GSE18105	Human	110	16	94	0
GSE20916	Human	145	44	91	10
GSE13067	Human	73	0	73	0
GSE9348	Human	82	12	70	0
GSE17538	Human	64	0	64	0
GSE26906	Human	57	0	57	0
GSE18088	Human	53	0	53	0
GSE31595	Human	37	0	37	0
GSE4183	Human	38	8	15	15
GSE4107	Human	20	10	10	0
GSE10714	Human	15	3	7	5
GSE15960	Human	18	6	6	6
GSE13471	Human	8	4	4	0
GSE10961	Human	4	0	4	0
GSE8671	Human	64	32	0	32
GSE9254	Human	18	18	0	0
GSE11831	Human	17	17	0	0
GSE31255	Human	11	0	0	11
**Model Validation**					
**Dataset Identifier**	**Species**	**No. of samples used**	**Normal colon**	**Cancer colon**	**Adenomas**
GSE77953	Human	58	30	28	-
PMID:26789870	Human	1832	170	1662	-
TCGA	Human	695	51	644	-
GSE20916	Human	70	34	36	-
GSE146009	Human	18	9	9	-
GSE44076	Human	196	98	98	-
GSE62932	Human	68	4	64	-
GSE83550	Mouse	194	-	-	-

GSE44988	Mouse	12	-	-	-
GSE50794	Mouse	42	-	-	-
GSE72347	Mouse	10	-	-	-
GSE43338	Mouse	14	-	-	-
GSE32697	Mouse	6	-	-	-
GSE31106	Mouse	6	-	-	-
E-MTAB-5249	Mouse	6	-	-	-
GSE64658	Mouse	28	-	-	-

Supplemental Table 3 [Related to Figure 3]: A catalogue of all covariates used in the multivariate analysis of the patient-derived organoids.

Catalog number	Age	Gender	Race	Primary Site	Acquisition Site	Clinical Stage Group	Histological Grade	APC	BRAF	KRAS	TP53	PTEN	PIK3CA	Number of mutated genes	Neoadjuvant therapy	IC50
PDM-255	73	Female	White	Colon	Cecum	Stage IVC	GB	no	no	no	no	no	no	218	yes	0.84
PDM-356	58	Male	Unknown	Colon	Sigmoid colon	ungrouped	G2	yes	no	no	yes	no	no	151	no	0.95
PDM-8	75	Male	Asian	Colon	Sigmoid colon	Stage IIA	G2	yes	no	no	yes	no	no	343	no	2
PDM-191	56	Male	White	Rectum	Sigmoid Colon	Stage IIIC	GX	no	yes	no	yes	no	no	219	yes	3.61
PDM-264	67	Female	White	Rectosigmoid junction	Rectosigmoid junction	Stage IIIB	G2	yes	no	no	yes	no	no	203	no	4.4
PDM-5	60	Female	White	Colon	Sigmoid colon	Stage IIIB	G2	yes	no	no	yes	no	no	131	no	4.5
PDM-275	73	Female	White	Colon	Lymph node(s)	ungrouped	ungrouped	no	no	no	no	no	no	211	yes	4.67
PDM-4	50	Male	White	Colon	Sigmoid colon	Stage IIIB	G2	yes	no	no	yes	no	no	102	no	5.14
PDM-95	61	Male	Black or African American	Colon	Sigmoid colon	Stage IIIB	GB	yes	no	no	yes	no	no	116	no	5.45
PDM-279	51	Male	Black	Colon	Cecum	Stage 0	GX	yes	yes	no	no	no	yes	173	no	6.9
PDM-2	68	Male	White	Colon	Sigmoid colon	Stage IVA	GB	yes	no	no	yes	no	no	129	yes	7.86
PDM-50	78	Male	White	Colon	Ascending colon	Stage IIIB	G3	no	no	no	no	no	no	0	no	8.53
PDM-9	63	Male	Asian	Colon	Liver	Stage IVA	G2	yes	no	no	yes	no	no	139	no	11.1
PDM-276	54	Male	Black or African American	Colon	Transverse colon	ungrouped	ungrouped	yes	no	yes	no	no	no	109	no	19.85
PDM-1	75	Male	White	Colon	Cecum	Stage I	G2	yes	no	yes	yes	no	no	191	no	27.9
PDM-185	71	Male	White	Colon	Cecum	Stage IIA	G2	yes	no	yes	no	no	no	154	no	40
PDM-94	67	Male	White	Colon	Sigmoid colon	ungrouped	G2	yes	no	no	yes	yes	no	168	no	40
PDM-7	75	Male	Black or African American	Colon	Sigmoid colon	Stage IIIA	G2	yes	no	yes	yes	no	no	156	no	40
PDM-257	53	Female	Unknown	Rectosigmoid junction	Rectosigmoid junction	Stage IIIB	G2	yes	no	yes	yes	yes	no	155	no	40
PDM-363	72	Male	Unknown	Colon	Cecum	ungrouped	GX	yes	yes	no	yes	yes	yes	2701	no	40
PDM-277	76	Female	White	Colon	Lymph node(s)	Stage IVA	G2	yes	no	yes	yes	no	no	209	no	40

PDM-359	64	Female	Unknown	Colon	Ascending colon	ungrouped	G2	yes	yes	no	no	no	yes	1502	no	40
PDM-103	51	Female	Black or African American	Colon	Liver	ungrouped	ungrouped	yes	no	yes	yes	no	no	171	yes	40

Supplemental Table 7 **[Related to Figure 5 and S7]:** A catalogue of all odds ratios and associated statistics for the 50-gene signature and CDX2 alone across various publicly available datasets.

50 gene signature	
Marisa-GSE39582 (n = 545; Colorectal cancers; cohort enriched for KRAS/BRAF mut) [Entire cohort]	
Odds ratio	1.546
95 % CI:	1.0826 to 2.2077
z statistic	2.397
Significance level	P = 0.0166
Hu-GSE87211 (n = 353; Colon cancers)	
Odds ratio	2.1917
95 % CI:	1.3313 to 3.6082
z statistic	3.085
Significance level	P = 0.0020
Gaedcke-GSE40492 (n = 219; Rectal cancers)	
Odds ratio	1.7234
95 % CI:	0.8983 to 3.3065
z statistic	1.637
Significance level	P = 0.1015
Jorissen-GSE13067, GSE14333; Smith-GSE17538; Medema-GSE33113; Thorsteinsson-GSE31595; Olschwang-GSE37892 (Pooled: n = 555; Colon cancers)	
Odds ratio	2.1306
95 % CI:	1.4365 to 3.1599
z statistic	3.761
Significance level	P = 0.0002
CDX2	
Marisa-GSE39582 (n = 545; Colorectal cancers; cohort enriched for KRAS/BRAF mut) [Entire cohort]	
Odds ratio	0.6751
95 % CI:	0.4424 to 1.0301
z statistic	1.822
Significance level	P = 0.0684
Hu-GSE87211 (n = 353; Colon cancers)	
Odds ratio	0.86
95 % CI:	0.5096 to 1.4512
z statistic	0.565
Significance level	P = 0.5721
Gaedcke-GSE40492 (n = 219; Rectal cancers)	
Odds ratio	1.0074
95 % CI:	0.4579 to 2.2163
z statistic	0.018
Significance level	P = 0.9854

Jorissen-GSE13067, GSE14333; Smith-GSE17538; Medema-GSE33113; Thorsteinsson-GSE31595; Olschwang-GSE37892 (Pooled: n = 555; Colon cancers)	
Odds ratio	0.5603
95 % CI:	0.3737 to 0.8399
z statistic	2.805
Significance level	P = 0.005
