# Supplementary material for: CANDiT: A machine learning framework for differentiation therapy in colorectal cancer
Source: Cell Rep Med. 2025 Oct 20;6(11):102421. doi: 10.1016/j.xcrm.2025.102421 (PMC12711679; doi:10.1016/j.xcrm.2025.102421)
Supplement: Document S2. Article plus supplemental information [file mmc6.pdf]

Cell Reports
Medicine

Article

CANDiT: A machine learning framework for differentiation therapy in colorectal cancer

Graphical abstract

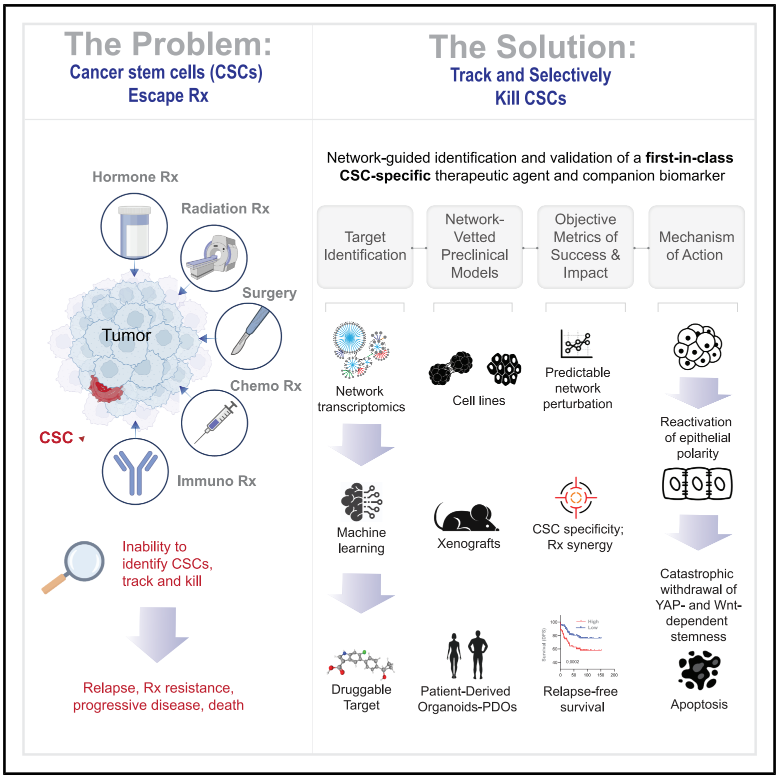

Authors

Saptarshi Sinha, Joshua Alcantara, Kevin Perry, ..., Jerry Yang, Michael Bouvet, Pradipta Ghosh

Correspondence

prghosh@ucsd.edu

In brief

Sinha et al. develop a machine learning–driven systems biology framework (CANDiT) to identify differentiation therapy targets in colorectal cancers (CRCs). Reactivating intestinal lineage regulators selectively eliminates cancer stem cells (CSCs), uncovering an unexpected therapeutic precision that reduces recurrence and enhances treatment specificity.

Highlights

- A machine learning framework nominates targets for CRC differentiation therapy
- Treatment induces crypt maturation and selectively kills CSCs
- Low CDX2 expression defines a vulnerable CSC state and predicts response
- Therapy dismantles stemness via reactivation of stress polarity signaling

Sinha et al., 2025, Cell Reports Medicine *6*, 102421
November 18, 2025 © 2025 The Author(s). Published by Elsevier Inc.
https://doi.org/10.1016/j.xcrm.2025.102421

CellPress

Cell Reports Medicine

CellPress
OPEN ACCESS

Article

CANDiT: A machine learning framework for differentiation therapy in colorectal cancer

Saptarshi Sinha,[1] Joshua Alcantara,[1,2] Kevin Perry,[1,2] Vanessa Castillo,[1] Annelies K. Ondersma,[1,2] Satarupa Banerjee,[1] Ella McLaren,[1,2] Celia R. Espinoza,[1] Sahar Taheri,[3] Eleadah Vidales,[1,2] Courtney Tindle,[1,2] Adel Adel,[1] Siamak Amirfakhri,[4,5] Joseph R. Sawires,[6] Jerry Yang,[6] Michael Bouvet,[4,5,7] and Pradipta Ghosh[1,2,7,8,9,*]

[1]Department of Cellular and Molecular Medicine, School of Medicine, University of California, San Diego, La Jolla, CA 92093, USA
[2]HUMANOID™ Center of Research Excellence, University of California, San Diego, La Jolla, CA 92093, USA
[3]Department of Computer Science and Engineering, Jacob's School of Engineering, University of California, San Diego, San Diego, CA 92093, USA
[4]Department of Surgery, University of California, San Diego, San Diego, CA 92093, USA
[5]Department of Surgery, VA San Diego Healthcare System, San Diego, CA, USA
[6]Department of Chemistry and Biochemistry, University of California, San Diego, San Diego, CA 92093, USA
[7]Moores Cancer Center, University of California, San Diego, San Diego, CA 92093, USA
[8]Department of Medicine, University of California, San Diego, San Diego, CA 92093, USA
[9]Lead contact
*Correspondence: prghosh@ucsd.edu
https://doi.org/10.1016/j.xcrm.2025.102421

SUMMARY

Reactivating lineage commitment to differentiate, and hence eliminate, cancer stem cells (CSCs) remains a therapeutic challenge. Here, we present CANDiT (cancer-associated nodes for differentiation targeting), a machine learning framework that identifies transcriptomic vulnerabilities for differentiation therapy in colorectal cancer (CRC). Centering on CDX2—a master intestinal lineage factor lost in high-risk, poorly differentiated CRCs—we identify PRKAB1, a stress polarity sensor, as a top therapeutic target. A clinical-grade PRKAB1 agonist reactivates lineage programs, dismantles Wnt/YAP-driven stemness, and selectively eliminates CDX2-low CSCs across CRC cell lines, xenografts, and patient-derived organoids (PDOs). Multivariate analysis reveals a strong therapeutic index tied to the CDX2-low state. A 50-gene response signature, derived from integrated modeling across all platforms, predicts ~50% reduction in recurrence and mortality risk. Like immunotherapy, CANDiT resurrects a physiologic program—differentiation—to selectively eliminate CSCs, offering a scalable, precision framework for lineage restoration in solid tumors.

INTRODUCTION

Poor differentiation, marked by elevated stemness, is a hallmark of cancers. Differentiation therapy, which targets cancer stem cells (CSCs) to induce maturation, has shown success in hematologic malignancies—most notably with all-trans-retinoic acid (ATRA) in acute promyelocytic leukemia.[1–10] While other agents have also shown promise in leukemias,[11,12] differentiation therapy has not yet translated to carcinomas. A major obstacle is the profound intra- and inter-tumoral heterogeneity that obscures identification of CSCs.[13]

To address this, computational methods have been developed to interrogate transcriptomic data, construct gene networks, and identify therapeutic targets.[14–19] Traditional symmetric frameworks—such as correlation,[20–25] mutual information,[17] linear regression,[26] dimension reduction,[27] and clustering[28,29]—often fail in the face of real-world biological complexity. In contrast, Boolean implication-based network transcriptomics[30,31] uses asymmetric, invariant gene relationships to construct directed networks that map evolving cellular states. This approach has identified translationally relevant states and targets across tissues and contexts[32–45] with high degrees of precision, including a first-in-class therapeutic to protect gut barrier function in inflammatory bowel diseases.[30]

Here we applied this method to identify targets that restore expression of CDX2, a caudal-related homeobox transcription factor (TF) and tumor suppressor.[46–49] In 2016, an unbiased search (using the same Boolean logic approach[50,51]) identified CDX2 as the top marker of colonic epithelial differentiation.[52] Its expression inversely correlates with "activated leukocyte cell adhesion molecule" (*ALCAM*/CD166), a stem cell marker that is present at the crypt base[53,54] and on highly tumorigenic human colorectal cancer (CRC) cells.[55] CDX2 loss—seen in ~9% of CRCs—correlates with poor differentiation, worse prognosis, and enhanced chemotherapy benefit even in stage II disease.[52] Since then, 32 independent studies involving >13,000 patients have confirmed CDX2-low CRCs are associated with worse overall survival (OS) and disease-free survival (DFS), independent of stage, mismatch repair status, or ethnicity[56–70] (Figure 1-*Step 1*). One study integrated immunohistochemistry

This is an open access article under the CC BY license (http://creativecommons.org/licenses/by/4.0/).

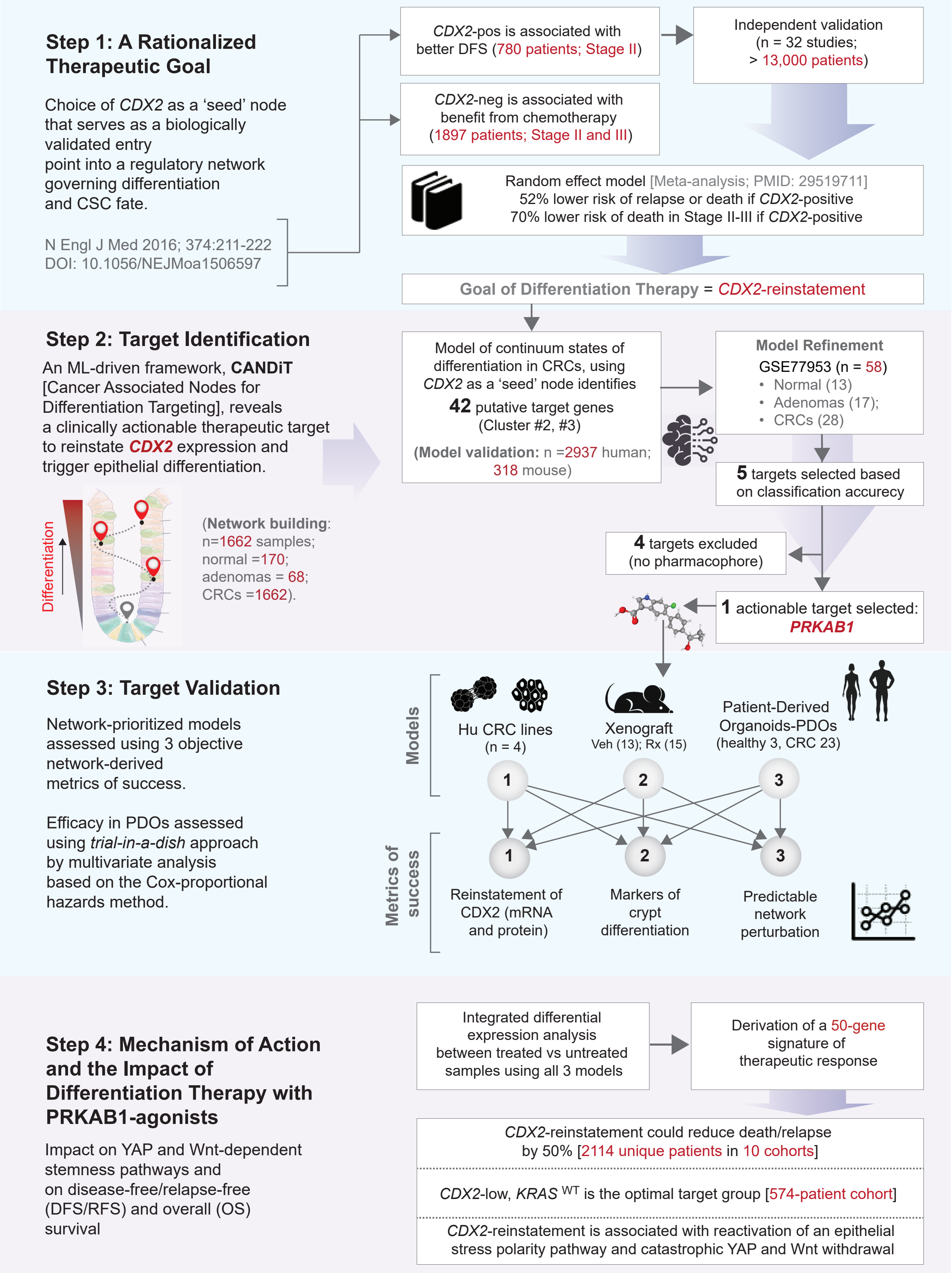

(legend on next page)

(IHC) with quantitative mass spectrometry.[59] Absence of CDX2 in tumors is associated with several adverse prognostic features, such as poor differentiation, advanced stage, vascular invasion, right-sided location, CpG island methylator phenotype (CIMP), and *BRAF* mutation.[71–73] Conversely, CDX2 presence lowers recurrence and mortality risk by ∼50% and 52%, respectively[69]—with even greater benefit (∼70%) in stage II–III CRCs. Collectively, these findings establish the restoration of CDX2 as a clinically meaningful and high-priority therapeutic goal (see Figure 1-*Step 1*). While its biological importance is widely recognized, no pharmacologic approach has yet succeeded in reliably inducing *CDX2*. Because *CDX2* alone is insufficient to fully activate the intestinal lineage program[74]—and that its precise regulation is critical to preserve mucosal architecture—exogenous expression approaches are impractical.

Here, we used network-transcriptomic analysis[50,51] to identify therapeutic targets that upregulate CDX2, triggering a network-wide, domino-like cascade that drives differentiation in CRC. We selected CDX2 as a biologically validated master regulator whose reactivation enables reprograms CSC fate,[75] and bridges cellular differentiation states to clinical outcomes, linking CSC behavior to patient prognosis.

RESULTS

A network-rationalized approach: CANDiT

We applied the machine learning-based computational platform, Boolean Network Explorer (*BoNE*),[30] and *CDX2* as a "seed" gene (i.e., node) to model progressive gene regulatory events during colonic differentiation (Figure 1-*Step 2*; Figures S1A and S1B). This framework—*CANDiT* (cancer-associated nodes for differentiation targeting)—prioritizes actionable differentiation nodes in cancer networks to enable therapeutic reprogramming. The model was first trained on a maximally heterogeneous dataset comprising 1,662 CRCs, 68 adenomas, and 170 normal tissues (Figures S1C and S1D; Table S1), and subsequently refined using statistical learning on an independent dataset (GEO: GSE77953). The resulting Clustered Boolean Implication Network (CBIN) comprises nine gene-clusters (Table S2). Classification accuracy identified five statistically top-ranked target genes from *CDX2*-proximal clusters (2 and 3; Figure S1D) that are predicted to upregulate *CDX2* when activated (Figure 1-*Step 2*; Figure S1E). These predictions were validated in 2,937 additional human colon samples (Figure S1F) and 318 mouse samples (Figure S1G), confirming cross-species and cross-cohort robustness (Table S1).

To identify potent, selective modulators of the five targets, we queried the Protein DataBank (PDB; https://www.rcsb.org) for structure-resolved agonists. Selective ligands were identified only for PRKAB1 (25 molecules/structures); none were found for the other targets. Cross-referencing these hits in ClinicalTrials.gov yielded PF-06409577 (PF), a clinical-grade PRKAB1 agonist. Although not yet approved for a clinical indication, PF has been deemed safe in a randomized, double-blind, placebo-controlled phase 1 trial (NCT02286882[76]). PF was recently shown to specifically upregulate PRKAB1 at both transcript and protein levels in the colonic epithelium *ex vivo* and in mouse colon *in vivo*.[30]

The *CANDiT*-inferred network predicted that agonizing PRKAB1, the protein product of *PRKAB1* (protein kinase AMP-activated non-catalytic subunit beta-1), would (1) upregulate *PRKAB1* itself and co-clustered genes (via "equivalent" relationships), (2) induce *CDX2* and its proximal clusters (2 and #) via "hi⇒hi" links, and (3) repress stemness-associated genes (e.g., *CCDC88A*, cluster 9) via "hi⇒lo" links (Figure S1D). This regulatory logic holds consistently across diverse human and mouse CRC datasets, including early-onset CRCs (Figures S2A–S2D). Further supporting its selection, *PRKAB1*—but not its paralog *PRKAB2*—is highly expressed in the gastrointestinal tract[30] and PRKAB1 agonists promote epithelial polarity in the gut.[77–80]

For validation studies *in cellulo* (CRC cell lines), *in vivo* (murine xenografts) and *ex vivo* (a "living biobank" of patient-derived organoids [PDOs]), we prioritized models with contrasting CDX2 expression (high vs. low, as determined based on a threshold that was determined using *StepMiner*[81]; see STAR Methods): CDX2-low (target phenotype) and CDX2-high (negative control). In addition to conventional anti-tumor readouts, three pre-specified success metrics were used to evaluate therapeutic efficacy; all three models were required to meet all three endpoints (see Figure 1-*Step 3*). Previous attempts to induce CDX2 transiently,[82] failed to produce durable differentiation and showed inconsistent impact across these same metrics (Figures S2E–S2H). Finally, through an integrated transcriptomic approach validated in tumor tissues, we define the molecular mechanism of action and translational impact of successful *CDX2* reinstatement via PRKAB1 agonism (see Figure 1-*Step 4*).

Target validation on CRC cell lines and xenograft models

We prioritized two poorly differentiated CRC cell lines—HCT116 and SW480—which exhibited the lowest CDX2 transcript levels among 26 sequenced CRC cell lines profiled by microarray (GEO: GSE10843)—as optimal models for hypothesis testing. Two well-differentiated, *CDX2*-high cell lines (DLD1, $KRAS^{mut}$ and Caco2, $KRAS^{WT}$) were selected as a negative

Figure 1. Study design: Logical network perturbation to reinvigorate CDX2 expression as a therapeutic strategy in CRCs

Step 1. Flowchart outlines the chronological accumulation of evidence supporting *CDX2*'s biological role as a master regulator of intestinal epithelial differentiation and its consistent association with poor outcomes.[75]

Step 2. Key steps in the network-based identification of PRKAB1 as a potent, actionable target, whose agonism is predicted to restore CDX2 expression. See Table S1 for datasets used in model training and validation, and Table S2 for gene clusters co-induced with *CDX2* upon PRKAB1 activation.

Step 3. Three experimental models and objective criteria used to validate PRKAB1 agonism as a strategy for CDX2 reinstatement and induction of cellular differentiation.

Step 4. Computationally driven insights into underlying mechanism of action and therapeutic impact. CDX2-reinstatement may halve the risk of death/relapse and is associated with a catastrophic collapse of signals that sustain cancer stemness—programs to which CSCs, but not other cancer cells are addicted—and with the reactivation of an epithelial stress polarity pathway that is selectively lost in cancers.

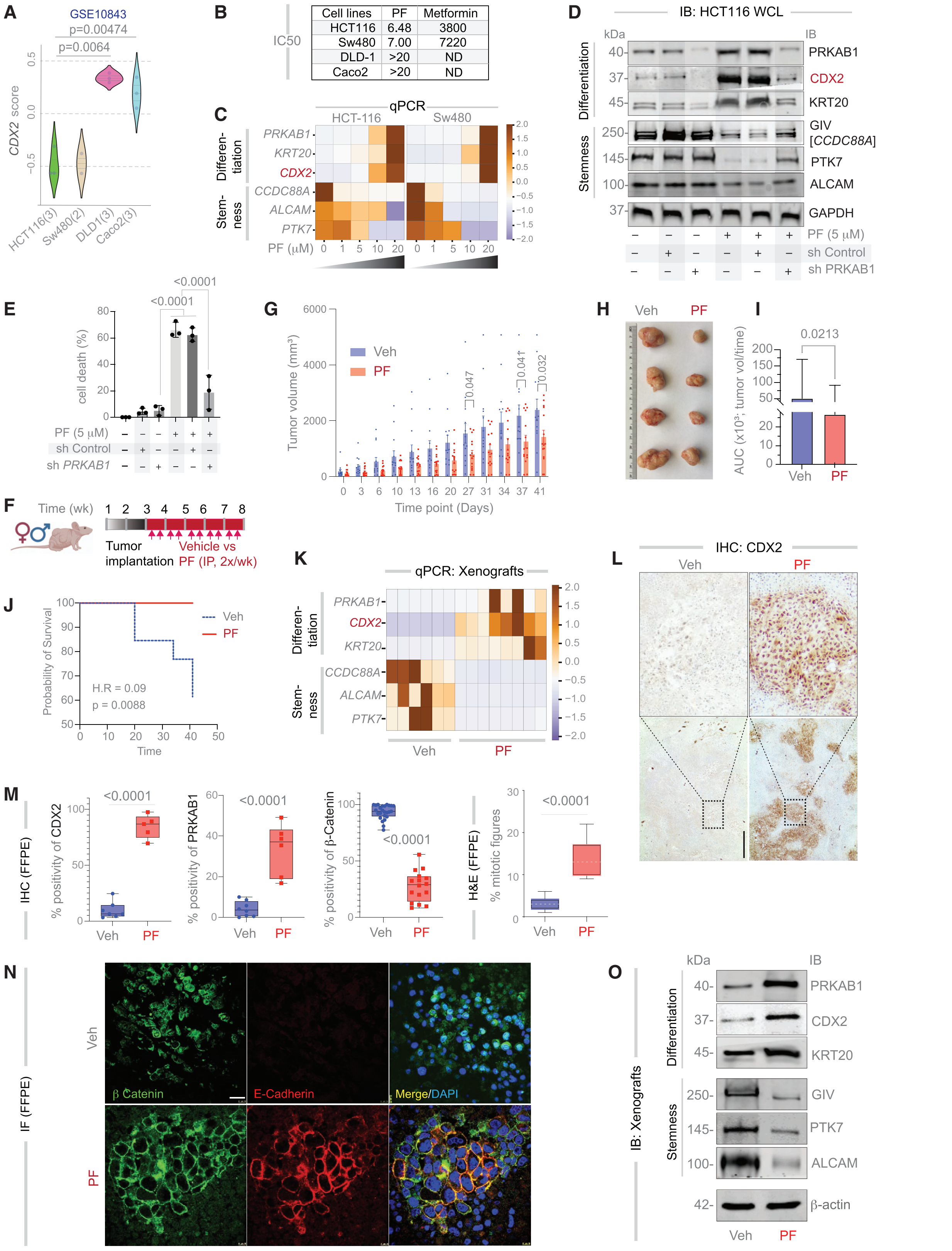

(legend on next page)

control (Figure 2A). PF induced cell death within 48 h in CDX2-low HCT116 and SW480 cells (IC_{50} ~6–7 μM), but not in *CDX2*-high DLD1 or Caco2 cells (IC_{50} > 20 μM; Figures 2B and S3A–S3C). In both *CDX2*-low cell lines, PF-induced cell death was preceded by dose-dependent induction of differentiation and suppression of stemness markers, at both transcript (Figure 2C) and protein (Figures S3D–S3G) levels. These effects coincided with a dose-dependent increase in apoptosis (Figures S4A–S4H). Importantly, PF failed to reinstate CDX2, alter differentiation or stemness markers (Figure 2D), or induce cell death (Figure 2E) in *PRKAB1*-depleted cells, confirming target specificity of its anti-cancer effects. In contrast, metformin—a non-specific, indirect agonist of AMP-kinase (activating both PRKAB1- and PRKAB2-containing complexes)—neither induced CDX2 expression nor triggered cell death, even at high doses (Figures 2B and S4I–S4K), underscoring the necessity of PRKAB1-specific activation for therapeutic efficacy.

In vivo, HCT116 xenografts implanted subcutaneously in nude mice exhibited a 68% reduction in tumor volume with PF treatment (area under the curve [AUC]: vehicle = 51,280.23; PF = 26,676.77; Figures 2G–2I) and a 62.5% increase in survival (Figure 2J; Mantel-Haenszel hazard ratio = 0.09 for reduction in death rates). PF-treated tumors developed glandular structures (Figure S5A), reinstated *CDX2* expression, and showed increased differentiation and reduced stemness markers by qPCR (Figure 2K). IHC confirmed nuclear localization of CDX2 and upregulation of PRKAB1 protein (IHC; Figures 2L, 2M, and S5B). Reflecting the slow division rate of colonic stem cells,[83] PF triggered a ~3-fold increase in mitotic bodies (Figures S5C and 2M). Additional signs of tumor differentiation included a reduction in nuclear β-catenin (Figure S5D) and a corresponding increase in junctional β-catenin (Figure 2N), which co-localized with E-cadherin (Figures 2N and S5E).

Together, these findings demonstrate that CDX2-reinstatement therapy drives differentiation and apoptosis selectively in *CDX2*-low CRC models, both *in vitro* and *in vivo*, through PRKAB1-specific activation.

Target validation in patient-derived organoids

We next evaluated the therapeutic efficacy of PF in PDOs. To evaluate potential toxicity, we first tested PF on healthy colon PDOs using eTOX Red staining, which selectively labels dead cells based on membrane permeability (Figure 3A-*left*). At 5 and 20 μM, PF, there was no significant increase in cell death compared to vehicle control. As expected, hydrogen peroxide ($H_2O_{2,200\ nM}$), a positive control, induced significant cytotoxicity relative to both PF-treated and control groups (Figure 3A-*right*; Figure 3B). We then tested PF across two prospective cohorts comprising 23 CRC PDOs and 3 healthy colon PDOs (see key resources table and STAR Methods; Figure 3C). PF exhibited selective anti-cancer activity in CDX2-low CRC PDOs (as determined based on a threshold that was determined using *StepMiner*[81] cutoff; see STAR Methods), reflected by low IC_{50} values. In contrast, CDX2-high CRC PDOs and all healthy colon PDOs were resistant (IC_{50} > 40 μM; Figure 3D). Notably, IC_{50} values positively correlated with expression of CDX2 (seed gene) and PRKAB1 (network-derived target), while showing an inverse correlation with CCDC88A, a network-linked stemness marker (Figures S1D and 3D).

Using only these three network-derived genes (*CDX2, PRKAB1*, and *CCDC88A*), hierarchical unsupervised clustering accurately classified sensitive versus resistant PDOs (Figure 3E). As in prior models, PF promoted differentiation and suppressed stemness markers in CRC PDOs (Figure 3F); effects were again restricted to CDX2-low tumors, consistent with findings from CRC cell lines (Figure 2C) and xenografts (Figure 2K).

Multivariate analysis of CRC PDOs (see Table S3 for covariates) identified CDX2 expression as the most significant predictor of IC_{50} (Figure 3G). Two additional variables also emerged: histological grade and *KRAS* mutation status. Specifically, PF was less effective in moderately or well-differentiated tumors that more frequently harbor KRAS mutations (Figure 3G). The interplay between CDX2 induction and *KRAS* status suggested a potential ceiling effect, i.e., CDX2 may remain constitutively high in $KRAS^{mut}$ tumors, limiting the therapeutic benefit of PF.

Figure 2. Target validation in CRC cell lines and xenograft models

(A) Violin plot of normalized *CDX2* expression in CRC cell lines. *p* values are calculated using Welch's *t* test.

(B) Table summarizing IC_{50} values (μM) for a specific (PF) or a non-specific (metformin) PRKAB1-agonist in CRC cell lines (*n* = 3 in each cell lines), measured by cellular impedance (cell index) assays at 48 h (see STAR Methods).

(C) RT-qPCR analysis of differentiation and stemness markers in >95% viable cells (16 h post-treatment), displayed as a *Z* score heatmap.

(D and E) Immunoblots (D) and MTT-based survival assays (E) showing the effects of PRKAB1 depletion (via shRNA in HCT116 cells) on the anti-cancer efficacy of PF. *p* values via one-way ANOVA; error bars represent SEM of three independent biological replicates. See Figures S3 and S4 for additional dose-response studies using PF (S3 and S4A–S4H) and metformin (S4I–S4K).

(F) Schematic of the xenograft experimental design and workflow.

(G) Tumor growth kinetics in vehicle-treated (Veh; *n* = 13) vs. PF-treated (*n* = 15) mice, measured twice weekly with calipers across three experimental cohorts. *p* values by multiple paired *t* tests with Benjamini, Krieger, and Yekutieli correction.

(H) Representative images of xenograft tumors at 8 weeks post-implantation.

(I) Bar plots comparing area under the curve (AUC) for tumor growth: Veh (95% CI: 31,788–70,772) vs. PF (95% CI: 16,949–36,404). *p* value by unpaired two-tailed *t* test; error bars indicate SEM.

(J) Kaplan-Meier analysis of time to IACUC-mandated endpoints (euthanasia due to death or discomfort). *p* value was derived via log rank test; H.R., hazard ratio.

(K) RT-qPCR analysis of differentiation and stemness markers in xenografts displayed as a heatmap of *Z* score normalized values.

(L) Representative images of CDX2 immunostaining in formalin-fixed paraffin-embedded (FFPE) xenografts. Scale bars, 100 μm.

(M) Quantification of CDX2, PRKAB1, and nuclear β-catenin (via IHC Profiler in ImageJ) and mitotic bodies (manually counted in H&E sections). *p* values are calculated by unpaired two-tailed *t* test. See Figures S5B–S5D for images from representative fields.

(N) Representative fields from FFPE tumors co-stained for E-cadherin (red), β-catenin (green) and DAPI (nuclei; blue) are shown. Scale bars, 20 μm. See also Figure S5K for colocalization analysis using ImageJ.

(O) Immunoblots from viable tumor lysates. See Figure S5F for quantification.

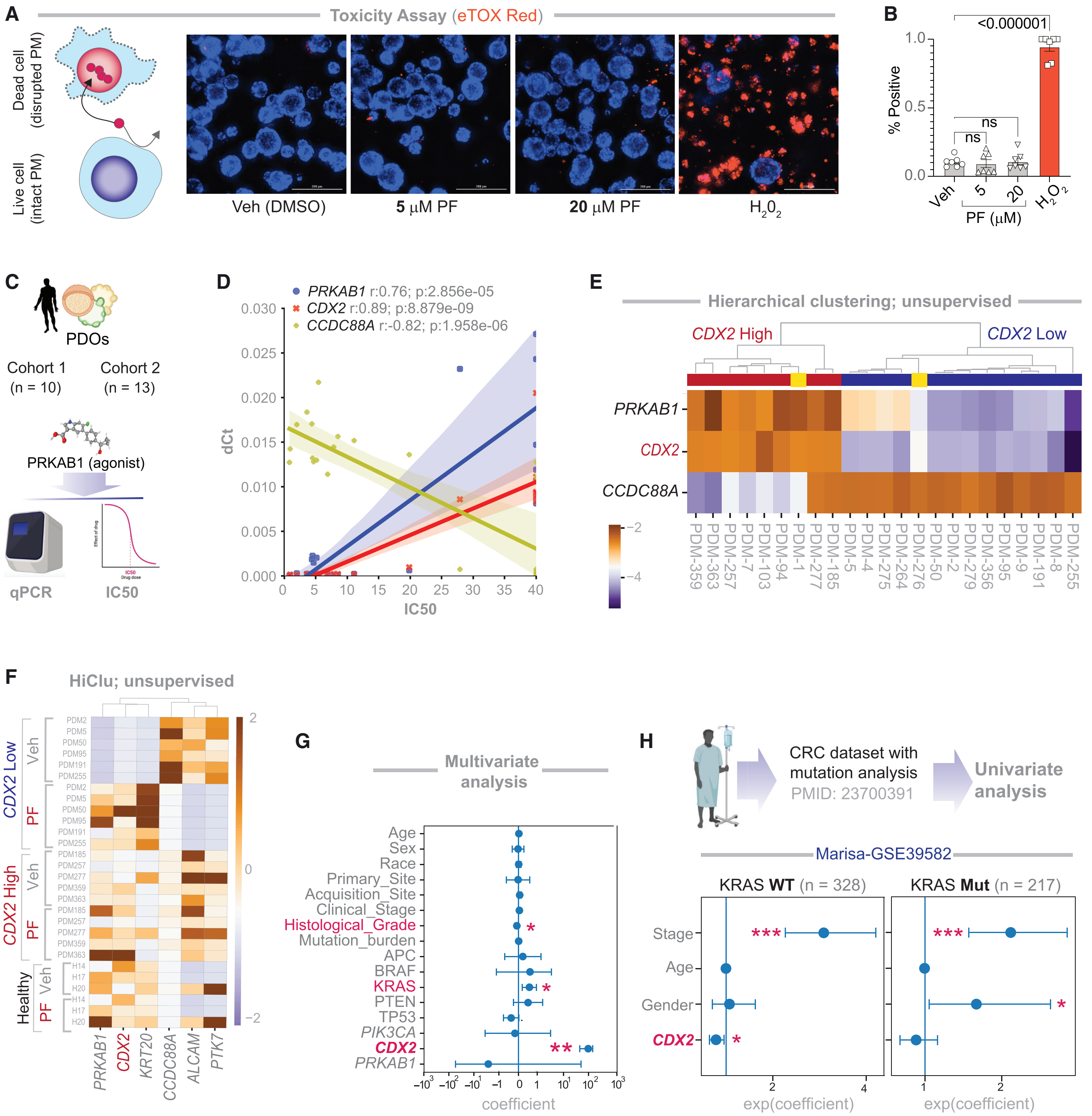

Figure 3. Differentiation and anti-cancer effects of PRKAB1 agonism are restricted to *CDX2*-low PDOs

(A) Schematic illustrating the principle of eTOX Red staining, which selectively labels dead cells based on increased membrane permeability (left). Representative immunofluorescence images show healthy colon PDOs treated with vehicle (control), 5 μM PF, 10 μM PF, or H_2O_2 (positive control for cell death) (right). Scale bars, 200 μm.

(B) Quantification of the % of marker-positive (dead) organoids from IF images shown in (A) (right). Statistical comparisons between treatment groups ($n = 7$) were performed using one-way ANOVA.

(C) Schematic of the experimental workflow showing sequential analyses conducted in two independent patient cohorts. See Table S3 for patient demographics, tumor characteristics, and mutation status of key genes.

(D) Correlation plots showing the relationship between IC_{50} values for PF and mRNA expression levels of 3 network-derived genes, as determined by qPCR.

(E) Heatmap showing unsupervised clustering of CRC PDOs based on *Z* score-normalized qPCR estimation of the 3 genes in D.

(legend continued on next page)

A univariate analysis in a large CRC cohort with known driver mutations and patient outcomes confirmed this relationship: CDX2 was prognostic in *KRAS*^{WT} tumors (Figure 3H-*left*), but not in *KRAS*^{mut} tumors (Figure 3H-*right*).

These findings indicate that despite its modest size, our PDO cohort captures clinically relevant heterogeneity and reveals a key interaction between *CDX2* status and *KRAS* mutation in determining therapeutic response.

Indicators of therapeutic efficacy and target cell specificity

RNA sequencing (RNA-seq) across all three model systems—CRC cell lines, xenografts, and PDOs—confirmed that PRKAB1 agonism met the three *a priori* defined therapeutic success metrics: (1) reinstatement of CDX2, the primary therapeutic goal (bubble plots in Figure 4A-*top row*; 4B; violin plots in Figures S6A–S6C), (2) induction of markers for crypt-top enterocytes and goblet cells, established surrogates of differentiation in the colon crypt[51,84] (Figure 4A-*middle row*; Figures S6D–S6F), and (3) predictable perturbation of the network, specifically induction of genes in clusters 2 and 3 (Figure 4A-*bottom row*; Figures S6G–S6I). Importantly, these effects were observed exclusively in *CDX2*-low CRC models—cell lines, xenografts, and PDOs—but not in *CDX2*-high PDOs or healthy colon PDOs (Figure 4A-*columns 5 and 6*; Figure S6C, S6F, and S6I).

A recent study using a machine learning algorithm defined transcriptomic and epigenetic signatures that distinguish normal stem cells from their differentiated progeny, unveiling CSC-specific gene sets indicative of oncogenic dedifferentiation.[85] Applying these signatures, we found that CDX2-reinstatement therapy did not affect normal stem cell–related genes in either healthy or CRC PDOs (Figure 4C). In contrast, it selectively downregulated (1) genes associated with cancer-related stemness (Figure 4D), (2) Yamanaka factors that promote somatic cell reprogramming and are implicated in CSC origins[89] (Figure 4E), and (3) CRC-specific CSC gene signatures (Figure 4F).

These findings demonstrate that *CDX2*-reinstatement acts as a trigger for broader transcriptomic reprogramming that selectively suppresses CSC programs unique to *CDX2*-low tumors, while sparing normal stem cells in healthy colon and *CDX2*-high tumor cells.

Potential for synergy with other therapeutic modalities

CDX2-low stage II–III CRCs have previously been shown to derive benefit from adjuvant chemotherapy, whereas CDX2-high tumors do not.[52] We therefore asked whether therapeutic reinstatement of CDX2 in CDX2-low tumors would enhance responsiveness to chemotherapy. Across all models tested, PF treatment consistently altered patient-derived gene expression signatures, inducing those associated with favorable response and suppressing those linked to resistance to various chemotherapeutics (e.g., 5-FU, oxaliplatin, and FOLFIRI) (Figure 4Gi). In line with these transcriptional changes, PF (5 μM, 24 h) exhibited synergy with multiple chemotherapeutic agents (see Figure 4H legend).

We extended this analysis to other treatment modalities. PF similarly induced gene signatures predictive of response to anti-EGFR therapy (cetuximab; Figure 4Gii) and several tyrosine kinase inhibitors (TKIs; Figure 4Giii), with a few notable exceptions (afatinib and gefitinib; Figure 4Giii). It also enhanced signatures associated with immunotherapy response while suppressing those of non-response (Figure 4Giv). Consistent with this, PF robustly induced the consensus molecular subtype CMS1 across all three model systems (Figure 4Gv). This subtype is characterized by a favorable immune landscape and enhanced sensitivity to immune checkpoint inhibitors.[90] *In vivo* xenograft studies also revealed a treatment-associated reduction in the consensus molecular subtype CMS4, which is linked to poor prognosis and characterized by high stromal content and transforming growth factor β (TGF-β) signaling.[90,91] Consistent with this, PF treatment significantly reduced stromal infiltration, as determined by two independent gene expression signatures (see Figures 4I and 4J legend).

These findings suggest that *CDX2*-reinstatement acts as a trigger for broader transcriptomic reprogramming that favors existing treatment modalities, including chemotherapy, targeted therapies, and immunotherapy.

A molecular signature of therapeutic response and selectivity

To characterize the therapeutic response to CDX2-reinstatement and its impact on the broader transcriptome, we performed integrated differential expression analysis using RNA-seq data from all three models (CRC cell lines, xenografts, and PDOs; see STAR Methods). Differentially expressed genes (DEGs) from two independent models (cell lines and xenografts; Tables S4 and S5) were refined using data from the third (CRC PDOs), yielding a 50-gene signature (Figure 5A). These genes were significantly upregulated in *CDX2*-low CRC PDOs compared to healthy and *CDX2*-high CRC PDOs (Figures 5B, S7A, and S7B). Upon PF treatment, this signature was downregulated to levels comparable to healthy and *CDX2*-high CRC PDOs (Figure 5B; see Table S6), suggesting a shift from a stem-like to a differentiated state.

This 50-gene signature included several unique CSC-associated genes—*AGR2*,[93] *ASCL2*,[94,95] *ALDH3A1*,[96] and *TM4SF1*[97,98]—not previously captured in machine-learning-derived CSC signatures (Figures 4C–4F). Pathway enrichment analysis revealed activation of pro-survival programs known to sustain CSCs,[99–102] including PTK6-dependent JAK/STAT signaling, growth factor receptor and non-receptor tyrosine

(F) Heatmap comparing expression profiles of stemness and differentiation markers based on *Z* score-normalized qPCR estimation in vehicle- versus PF-treated CRC PDOs.
(G) Multivariate regression analysis of IC_{50} values using a linear model incorporating all measured variables. Bar plot shows the coefficient estimates (center values), 95% confidence intervals (error bars), and *p* values for each variable. The *p* value for each term tests the null hypothesis that the coefficient is equal to zero (no effect). Red = statistically significant covariates. $^{*}p \leq 0.05$ and $^{**}p \leq 0.01$. See Table S3 for source data.
(H) Univariate analysis of relapse-free survival (RFS) stratified by KRAS mutation status (WT vs. mutant) using database GEO: GSE39582. $^{*}p \leq 0.05$, $^{**}p \leq 0.01$, and $^{***}p \leq 0.001$.

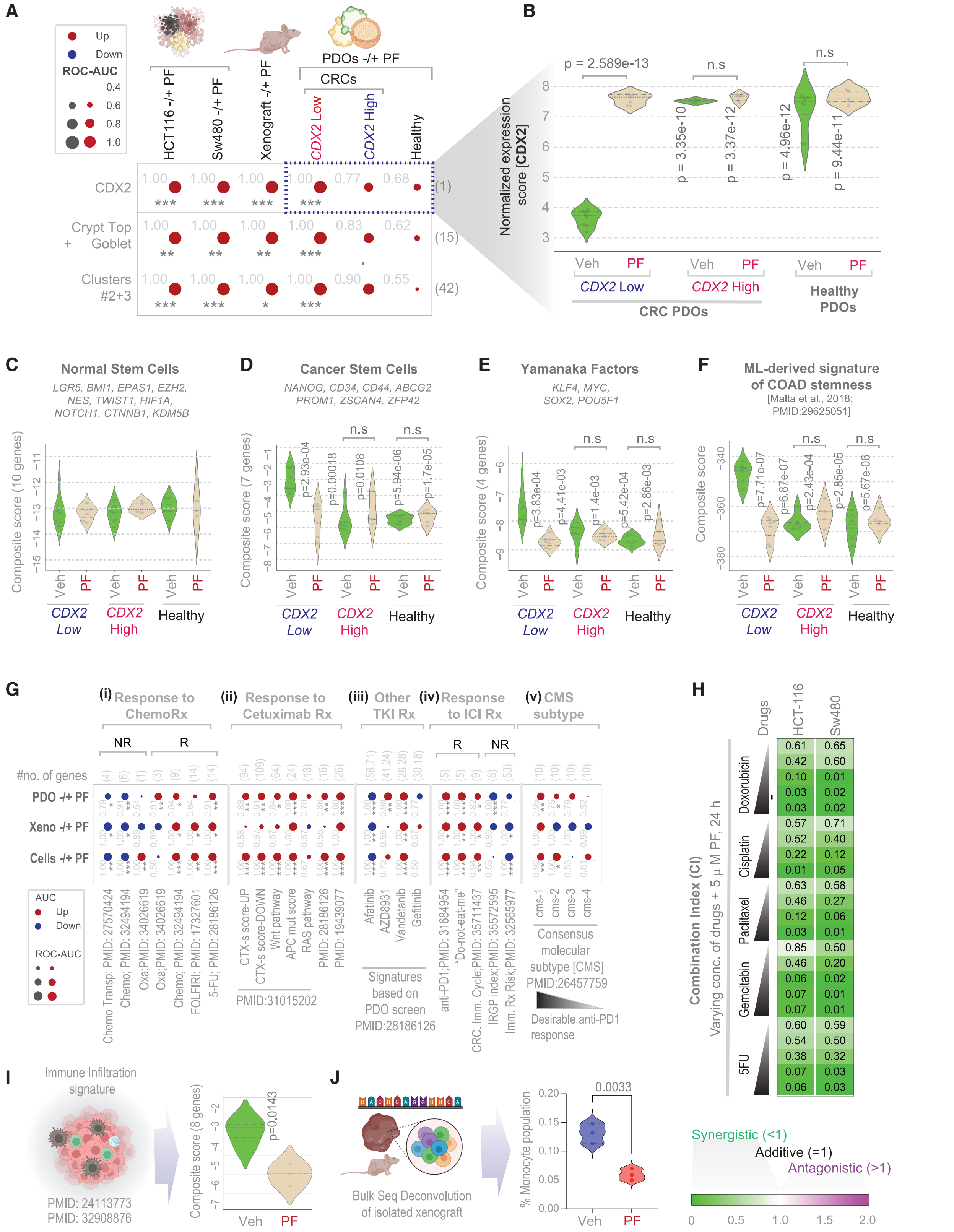

(legend on next page)

kinases, Hippo/YAP signaling, and TGF-β suppression (Figure 5C). Notably, these pathways were selectively downregulated in *CDX2*-low CRCs, but not in healthy or *CDX2*-high CRCs. Similar pathway enrichment analyses of the upregulated genes showed genes associated with epithelial structure maintenance (Figure S7C).

Impact of CDX2 reinstatement therapy on survival

To evaluate clinical relevance, we tested whether the 50-gene signature could stratify relapse-free/disease-free survival (RFS/DFS) in multiple CRC patient cohorts. Across all datasets, patients with low 50-gene scores—resembling PF-treated or *CDX2*-high tumors—had significantly better outcomes than those with high scores (Figures 5D and 5F–5K). The associated odds ratio was ∼2.0 (Table S7), indicating that elevated expression of the signature doubles the risk of recurrence or death—risk that may be mitigated by CDX2-reinstatement therapy.

Univariate analysis showed that both *CDX2* expression and the 50-gene signature were significant predictors of RFS (Figure 5E-*left*), but the signature performed better, especially in $KRAS^{WT}$ tumors (Figure 5E-*middle*). Across all patient cohorts, the 50-gene score consistently outperformed *CDX2* as a prognostic indicator (Figures S7D–S7G; Table S7). As seen previously for *CDX2* (Figure 3E), the 50-gene signature did not correlate with outcome in $KRAS^{mut}$ tumors (Figure 5E-*right*), suggesting that *KRAS* mutation may bypass the CDX2 axis. In fact, within the $KRAS^{WT}$ group, patients with low 50-gene scores resembling PF-treated tumors had significantly better outcomes than those with high scores regardless of microsatellite stability status (Figures 5J–5L).

Together, these findings identify a robust, target-derived 50-gene signature that not only captures the molecular response to *CDX2*-reinstatement but also stratifies survival risk. Therapeutic suppression of this signature emerges as a measurable and meaningful objective, with potential to improve patient outcomes.

State-specific cell fates dictate selectivity of CDX2-restorative therapy

Hallmark pathway analysis revealed that *CDX2*-restoration via PF treatment consistently activated a transcriptional program across all three models centered on the restoration of epithelial integrity, including polarity and cell junction assembly (Figure 6A). Integration of downregulated genes across models identified a core set of commonly suppressed transcripts (Figure 6B), and TF enrichment analysis implicated key stemness-associated TFs, e.g., *TEAD4*, *SP1*, and *KLF1/4* as top regulators of these genes (Figure 6C).

Histological analysis of xenograft tumors reinforced these transcriptional findings. PF-treated tumors displayed a significantly increased number of asymmetrically stretched nuclei, consistent with chromatin reorganization during epithelial reprogramming (Figure 6D). These morphological changes were accompanied by pronounced alterations in Hippo signaling: PF reduced nuclear localization of YAP (Figure 6E) while increasing its inactive, phosphorylated (p)-Ser[87] form (Figure 6F). This is consistent with pSer[87]-mediated cytosolic retention and phosphodegron-dependent degradation of YAP oncogenic activity,[103] explaining its near-total loss in treated tumors (Figure 6E).

PRKAB1 is also known to activate the stress polarity signaling (SPS) pathway[77–79]; it is a sentinel system that integrates metabolic stress with epithelial polarity, coordinating cell-cell junction restoration under bioenergetic challenge (see Figure S8A). In normal epithelial cells, SPS is inducible and supports junctional reassembly, anchorage-dependent growth as monolayer, and epithelial barrier integrity.[30,78] In contrast, transformed cells lose this pathway,[78] enabling anchorage-independent growth and junctional disassembly (Figures S8B and S8C). Notably, (re)activation of SPS permits anchorage-independent growth of normal cells but not transformed cells[79], highlighting its potential to discriminate between cell states and a plausible reason for PF's ability to selectively target CSCs, but spare healthy stem cells. We found that PF induced the SPS pathway, as evidenced by upregulation of a canonical biomarker $pSer^{245}GIV$[30,78,79] (Figure 6G).

Next, we hypothesized that SPS reactivation preserves the function of normal stem cells while selectively dismantling the survival programs of CSCs, which rely on SPS pathway silencing. To test this, we analyzed expression scores for core transcriptional targets of oncogenic stemness in PF-treated healthy and CRC PDOs. PF significantly reduced the expression of *annexin A1* (Figure 6H), *MYC* (Figure 6I), and transcriptional

Figure 4. Indicators of therapeutic success, target-cell specificity, and potential for synergy with other treatment modalities

(A) Bubble plots display ROC-AUC values for the classification of vehicle (Veh) vs. PF-treated samples across multiple models (HCT116, SW480, xenografts, and PDOs). Shown are *CDX2* expression patters (top row), crypt-top and goblet cell markers (middle row), and genes from network clusters 2–3 (bottom row). Circle size reflects the magnitude of the ROC-AUC; color indicates direction of regulation in treated samples (red = upregulated, blue = downregulated). Statistical significance was calculated using Welch's *t* test and annotated using standard codes (''$p > 0.1$, '.'$p \leq 0.1$, *$p \leq 0.05$, **$p \leq 0.01$, and ***$p \leq 0.001$). See Figure S7 for the same data shown as violin plots with actual *p*-values.

(B) Violin plots show *CDX2* expression levels in PDOs. Comparisons are made against vehicle (Veh)-treated *CDX2*-low PDOs using Welch's *t* test.

(C–F) Violin plots display the composite score of stemness markers derived from a recent pan-cancer machine learning study.[85] Comparisons were made against vehicle (Veh)-treated *CDX2*-low PDOs using Welch's *t* test.

(G) Bubble plots display ROC-AUC values for gene signatures associated with response (default or indicated with "R") and non-response ("N.R.") to multiple therapeutic modalities, across CRC cell lines, xenografts, and PDOs. Circle size reflects ROC-AUC; colors indicate direction of gene regulation. Significance was assessed using Welch's *t* test and annotated as in (A).

(H) Heatmap shows combination index (CI) values derived from MTT assays in HCT116 and SW480 cell lines treated with a fixed concentration of PF (5 μM, 24 h) and increasing doses of chemotherapeutic agents. CI values were calculated using a previously established formula.[86]

(I) Violin plot shows the composite score of an 8-gene immune infiltration signature[87] (derived from a large pan-cancer study[88] [https://bioinformatics.mdanderson.org/estimate/] after training on outcome-annotated datasets) in PF-treated and vehicle (Veh) treated xenografts. *p* value was derived using Welch's *t* test.

(J) Violin plot shows the % of monocyte population in PF-treated vs. vehicle (Veh)-treated xenografts, estimated via bulk RNA-seq deconvolution. *p* value was derived using Welch's *t* test.

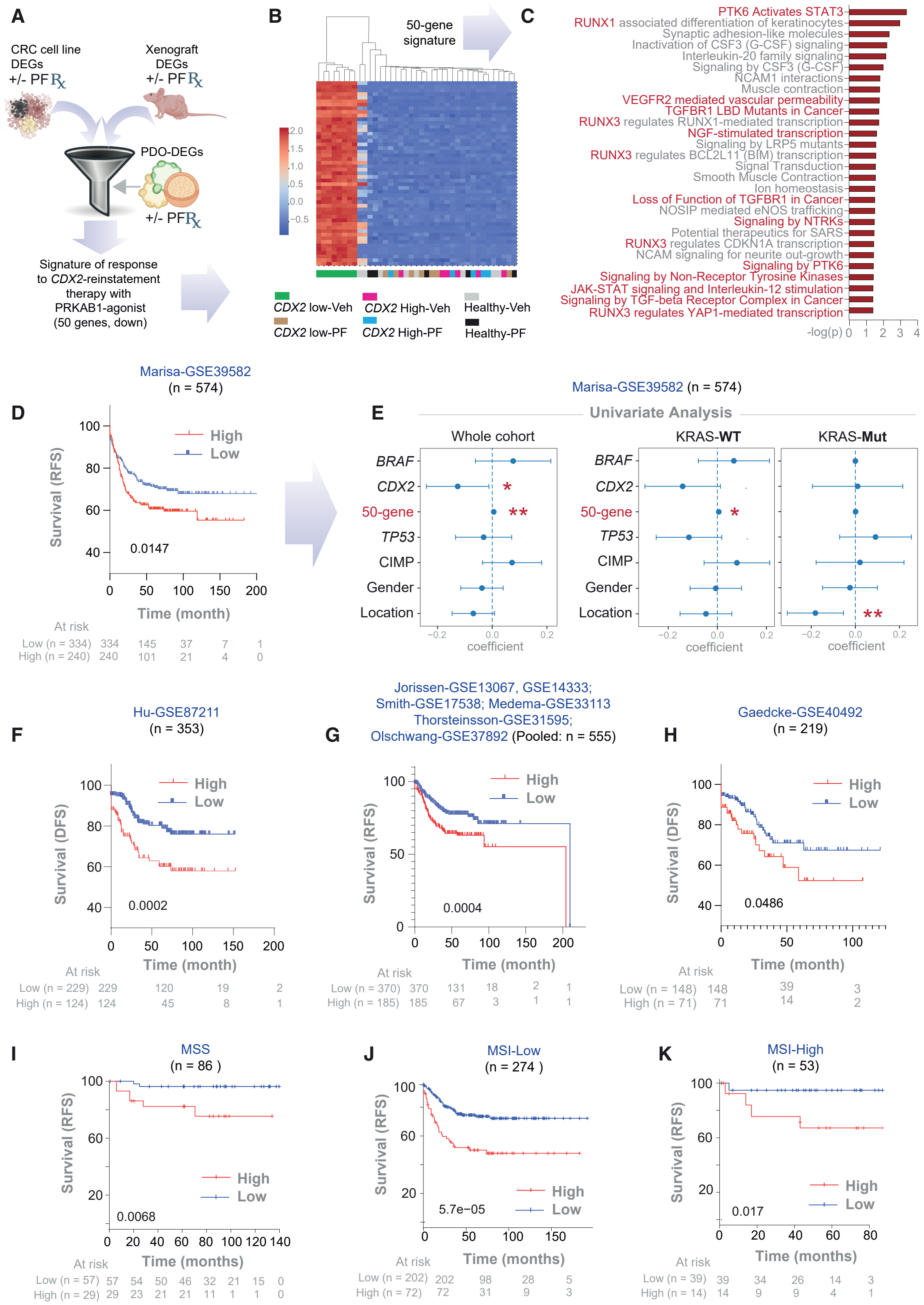

(legend on next page)

programs of Wnt/β-catenin (Figure 6J) and YAP (Figure 6K)—all of which were elevated in *CDX2*-low CSC-rich PDOs but not in healthy- or *CDX2*-high CSC-poor PDOs.

Together, these findings demonstrate that CDX2-reinstatement reprograms CSCs by restoring epithelial polarity through SPS pathway reactivation. This reactivation triggers the collapse of two key junction-informed transcriptional programs—YAP and Wnt/β-catenin—on which CDX2-low CSCs are critically dependent (see Figures 7A and 7B legend). This mechanism underpins the selectivity and efficacy of PF, driving differentiation in all cells but apoptotic fate specifically in CSCs (Figure 7C).

DISCUSSION

The central discovery of this study is a network-guided differentiation therapy that reinstates CDX2 expression to selectively eliminate CSCs in CRCs. Much like immunotherapy reawakens immune surveillance, CDX2-restorative therapy reactivates a physiologic lineage commitment. We identify *PRKAB1* as a key upstream node and show that PF-06409577, a clinically safe first-in-class agonist, triggers *CDX2*-restoration, cell-type specific differentiation, and anti-tumor responses. The therapeutic effect meets rigorous, pre-defined benchmarks for efficacy: *CDX2* induction, a robust 50-gene companion biomarker signature, suppression of stemness and survival pathways, and sparing of normal stem cells. Additional analyses of human CRCs using a panel of treatment-perturbed genes promises a ∼50% reduction in relapse and mortality risk in *CDX2*-low CRCs.

A scalable framework for differentiation therapy

Our use of the BoNE enabled the development of *CANDiT*, a rational, systems-level framework for inducing differentiation in solid tumors. By using *CDX2*—a master regulator of intestinal lineage—as a seed node, BoNE traced upstream regulatory hubs capable of reinstating epithelial identity while suppressing CSC-associated programs in colorectal cancers. This approach is generalizable: master regulators of differentiation in other tissues can similarly be used to drive lineage restoration, examples of which are emerging.[109]

Although *CDX2* is insufficient to fully activate the lineage program,[74] our findings suggest that PRKAB1 agonism by PF enables broader commitment by sustaining *CDX2* induction and engaging additional, underexplored and incompletely mapped pathways essential for terminal differentiation. *CANDiT* distinguishes itself from conventional screening methods by revealing hidden transcriptomic vulnerabilities in dedifferentiated CSCs and pinpointing high-value targets to exploit them. In the PRKAB1-activated state, *CDX2* plays a central but not solitary role in driving differentiation and CSC-specific death. Like *CDX2*, *PRKAB1* is likely to be necessary, but not sufficient to impose such broader commitment to differentiation. Our findings exemplify how *CANDiT* offers a scalable, mechanistically grounded blueprint for developing lineage-based therapies across solid tumors, guided by tissue-specific master regulators.

Mechanism of selectivity

We show that CDX2-restorative therapy exploits selective vulnerability in CSCs by reactivating the SPS pathway, a circuit integrating bioenergetic stress with epithelial polarity in 3D growth contexts. While this pathway is silenced in cancer, it remains functional in healthy tissues.[77,78] PRKAB1 agonism restores epithelial polarity through SPS activation, disrupting anchorage-independent growth and silencing junction-informed oncogenic programs (e.g., YAP, Wnt/β-catenin; Figures 7A and 7B), leading to a collapse of stemness signaling in CSCs. In contrast, in normal epithelial and stem cells, SPS activation supports barrier function and preserves homeostasis. This cell-state-specific response explains the therapeutic selectivity of PF, enabling CSC-targeted killing while sparing healthy stem cells (see Figure 7C).

It is noteworthy that the Cancer Dependency Map (DepMap; https://depmap.org/portal/home/) includes data for HCT116 and SW480, but not for key lines used here such as DLD1 and Caco2. Among the cell lines represented, PF IC_{50} values do not correlate with CDX2 transcript abundance. Our mechanistic data—highlighting the role of 3D context and anchorage-independent signaling in PF response—offer a plausible explanation, aligning with DepMap's own push to include 3D IC_{50} datasets.[110]

Translational potential and clinical prioritization

We advocate translating these findings into clinical trials with a focus on CDX2-low CRCs, especially those with a high 50-gene response signature. CDX2-low status, as assessed by

Figure 5. A 50-gene signature for tracking therapeutic response and estimating impact on survival

(A) Computational workflow used to derive a 50-gene signature of therapeutic response through an integrated differential expression analysis (DEA) approach. Differentially expressed genes (DEGs) from CRC cell lines and xenografts (see Tables S4–S6) were further refined based on their ability to classify accurately PDO samples treated with or without PF (see Table S6 for gene list).

(B) Heatmap shows the results of unsupervised clustering of PDOs based on the 50-gene signature. Veh, vehicle. See Figures S8A and S8B for violin plot visualizations.

(C) Reactome pathway analysis of the 50-gene signature, highlighting key biological processes. See Figure S8C for enrichment of KEGG pathways.

(D and F–H) Kaplan-Meier (KM) plots show recurrence-free (RFS) or disease-free (DFS) survival in various CRC patient cohorts, stratified by high vs. low expression scores of the 50-gene signature based on *StepMiner* algorithm[81] computed within each cohort. *p* values were calculated using the log rank test. For comparison, see Figure S8C for KM plots on the same cohorts based on *CDX2* expression, computed using the *StepMiner* algorithm[81] within each cohort.

(I–K) KM plots show recurrence-free (RFS) survival in all *KRAS*^{WT} CRC patients available in KM-plotter database,[92] grouped by their microsatellite stability status. The patient groups are stratified by high vs. low expression scores of the 50-gene signature based on *auto selected best percentile cutoff* algorithm used in KM plotter.[92] *p* values were calculated using the log rank test.

(E) Univariate Cox regression analysis of RFS in a large CRC cohort. Plots display the coefficient for each variable (center point) with corresponding 95% confidence intervals (error bars) and associated *p* values. The analysis includes the 50-gene signature along with other clinical variables. Left: entire cohort; middle: KRAS wild-type CRCs; right: KRAS mutant CRCs. *$p \leq 0.05$ and **$p \leq 0.01$.

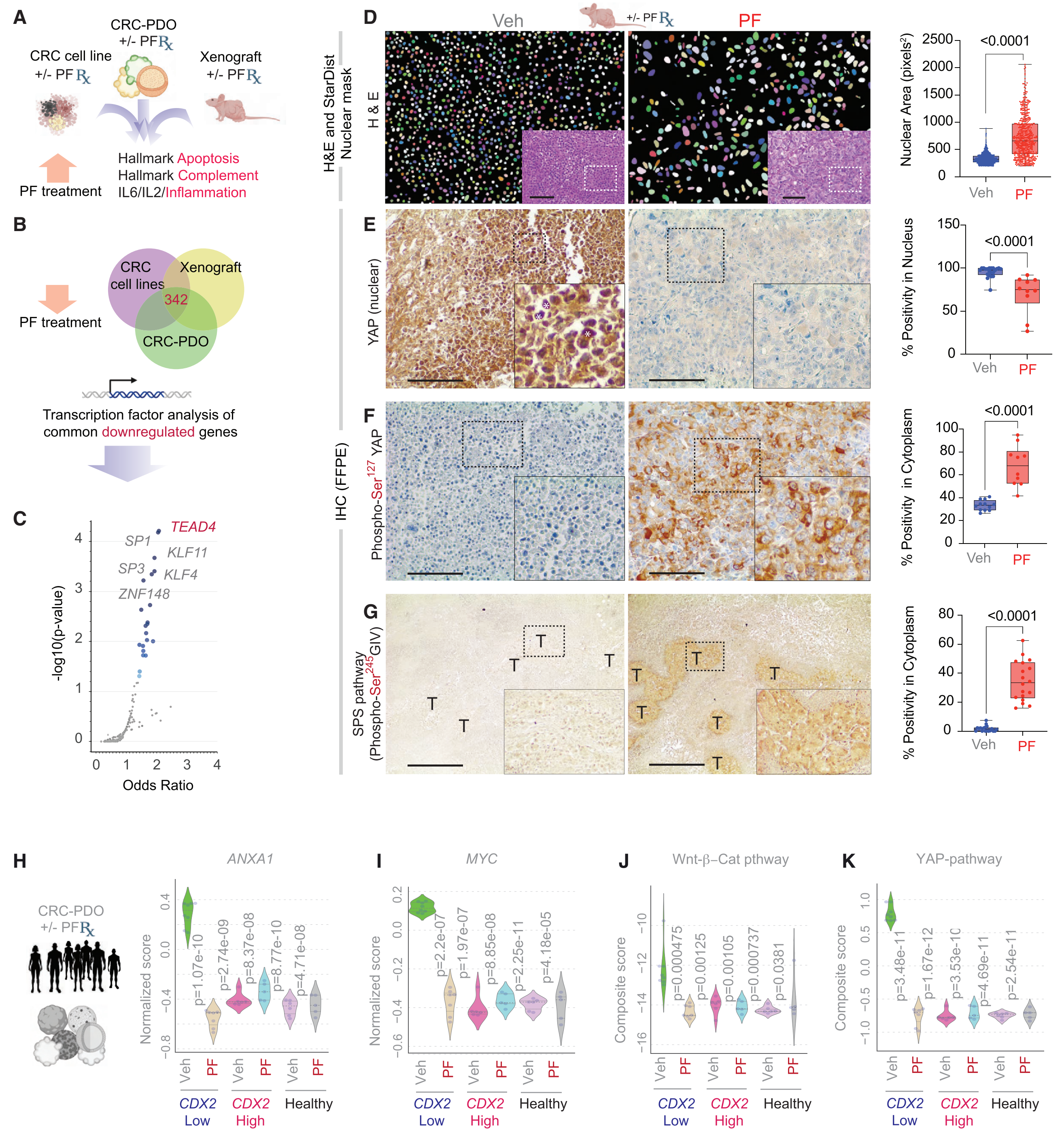

Figure 6. CDX2-reinstatement reprograms CRC stem cells by restoring epithelial polarity

(A) Schematic summarizes top hallmark pathways identified using integrated differential expression analysis (DEA) approach that are induced upon PF treatment in three model systems.

(B and C) Venn diagram (B) depicts downregulated genes common in all the three models used in this work. Transcription factor (TF) analysis (C) identified top TFs associated with common downregulated genes upon PF treatment in all the models.

(D) Representative H&E-stained sections (left, with inset) of FFPE xenograft tumors from vehicle (Veh) and PF-treated groups, with corresponding quantification of nuclear area (right) using StarDist-based segmentation in ImageJ. (n = 10 fields are analyzed).

(legend continued on next page)

IHC, is prognostic in stage III/IV disease and is frequently associated with *BRAF* mutations.[111] Conversely, *KRAS*^{mut} tumors tend to retain CDX2, reflecting the role of *KRAS*^{mut} in promoting hyperplasia[112] rather than stemness.[113] This intriguing distinction could be attributed to cell-type specific roles of *KRAS*/*BRAF*^{mut} reported recently.[114]

However, KRAS status alone should not preclude PF therapy: both HCT116 and SW480 cells (CDX2-low and *KRAS*^{mut}) were PF-sensitive. Notably, population studies confirm that a subset (~9%) of *KRAS*^{mut} metastatic CRCs with low CDX2 have poor prognosis[111] and could benefit from differentiation therapy. Thus, CDX2-low status and high therapeutic response signature, not KRAS or BRAF status, should drive clinical candidacy. Because loss of *CDX2* is an adverse prognostic factor in stage IV and stage III/*BRAF*^{mut} CRCs,[73,115] these subgroups can be prioritized for *CDX2*-reinstatement therapy with PRKAB1 agonist as single agent or together with other modalities.

Although *CDX2* is prognostic in stage II(MSS)[52,65,69] and stage III CRCs,[52,116] caution is warranted when combining PF with conventional chemotherapy. CDX2 expression is associated with MDR1 upregulation, which could contribute to chemoresistance.[117,118] Until further data are available, PF should be prioritized as monotherapy or in combination with non-cytotoxic agents in patients with relapsed or metastatic *CDX2*-low CRC. Finally, early-onset CRCs is a subgroup that show worse outcome (poorer DFS) than its late-onset counterparts.[119] Because the *CDX2-PRKAB1* relationship is conserved in this subgroup (Figure S2A), we propose including this group in future PF-focused trials.

Implications for pre-neoplastic disease

It has been recently shown that CDX2 loss plays a permissive role in CRC initiation, particularly along the serrated pathway[120–122] and in inflammation-associated CRCs.[123–125] Mouse models have shown that *CDX2*-deficient metaplastic cells can induce tumorigenesis non-cell-autonomously.[126,127] Our findings provide an impetus for future studies to evaluate whether *PRKAB1* agonists can serve as prophylactic agents in high-risk populations with pre-neoplastic lesions or chronic inflammation.

Conclusions

We present *CANDiT*, a machine learning-guided, network-based differentiation strategy that fills a critical gap in cancer therapy. *CANDiT* differs fundamentally from traditional pipelines in three key ways: (1) network-transcriptomics and ML-guided target discovery, (2) validation studies on network-vetted cell line and xenograft models, and (3) validation in PDOs utilizing biomarker-driven stratification and adjusting for confounding variables as done in clinical trial analysis. This strategy enables selective CSC targeting and spares normal stem cells. Despite strong evidence for CDX2 as a prognostic biomarker,[52,59,65,66,68,69,82,128,129] and a compelling pharmacoeconomic justification (i.e., cost $ 50 000 per QALY) for a CDX2-stratified decision to offer chemotherapy,[130] its clinical utility has not been adopted into guidelines due to lack of prospective validation. Our demonstration that CDX2 serves as both a predictive biomarker and therapeutic effector for PF, a drug already deemed safe in humans (NCT02286882), provides compelling justification for CDX2-guided clinical trials. More broadly, *CANDiT* opens the door to rational, scalable differentiation therapy across solid tumors.

Limitations of the study

The exact mechanism(s) linking PRKAB1, SPS activation, *CDX2* restoration and crypt differentiation was not fully delineated. Future studies will examine whether SPS (re)activation reverses aberrant *CDX2* promoter methylation.[106,107] The PDO cohort was limited in size but reflective of real-world diversity, capturing the interplay between tumor differentiation, CDX2 expression, and *KRAS*^{mut} status; larger cohorts and randomized clinical trials with RNA-seq endpoints is warranted. Epigenetic profiling and cell fate trajectory analysis will be essential to uncover the basis for the divergent cell fates—differentiation versus apoptosis—triggered by PF in normal vs. CSCs. We acknowledge that the prognostic role of CDX2 is nuanced and context-dependent across the histopathological and molecular spectrum of CRCs, not all captured in our PDO models. Thus, our findings should be viewed as a mechanistic proof-of-concept to target CSCs, rather than a universal paradigm to target cancers.

RESOURCE AVAILABILITY

Lead contact

Further information and requests for resources and reagents should be directed to and will be fulfilled by the lead contact, Pradipta Ghosh, prghosh@ucsd.edu.

Materials availability

This study has generated CRC organoid biobank, RNA and DNA from the organoids. These materials are available from the lead contact with a completed materials transfer agreement and patented technology agreement following the guidelines of the University of California, San Diego.

Data and code availability

- All transcriptomic datasets generated in this study have been deposited in the NCBI Gene Expression Omnibus (GEO) and are publicly available under accession numbers: GEO: GSE237623, GSE237624, and GSE237625.
- All code used for Boolean analysis framework is publicly available at https://github.com/sinha7290/Prodiff.
- The data underlying all the figures and tables are available in the article and its online supplemental materials. Any additional information

(E–G) Immunohistochemistry (IHC) images (left) and quantification (right) of xenografts showing expression of: nuclear YAP (E; white asterisk, inset); phospho-Ser[87] YAP (F); and phospho-Ser[245] GIV (G) (a biomarker of the SPS pathway[77–79]; see also Figure S8A). Scale bars, 100 μm. (n = 10–15 fields are analyzed in each case).
(H–K) Violin plots showing normalized expression scores in vehicle (Veh)- and PF-treated PDOs for transcriptional markers of oncogenic stemness: annexin (H), MYC (I), composite Wnt/β-catenin (J), and YAP (K) target genes (listed in Figure 7-*left*). p values were calculated using Welch's t test, comparing each sample to vehicle-treated *CDX2*-low PDOs.

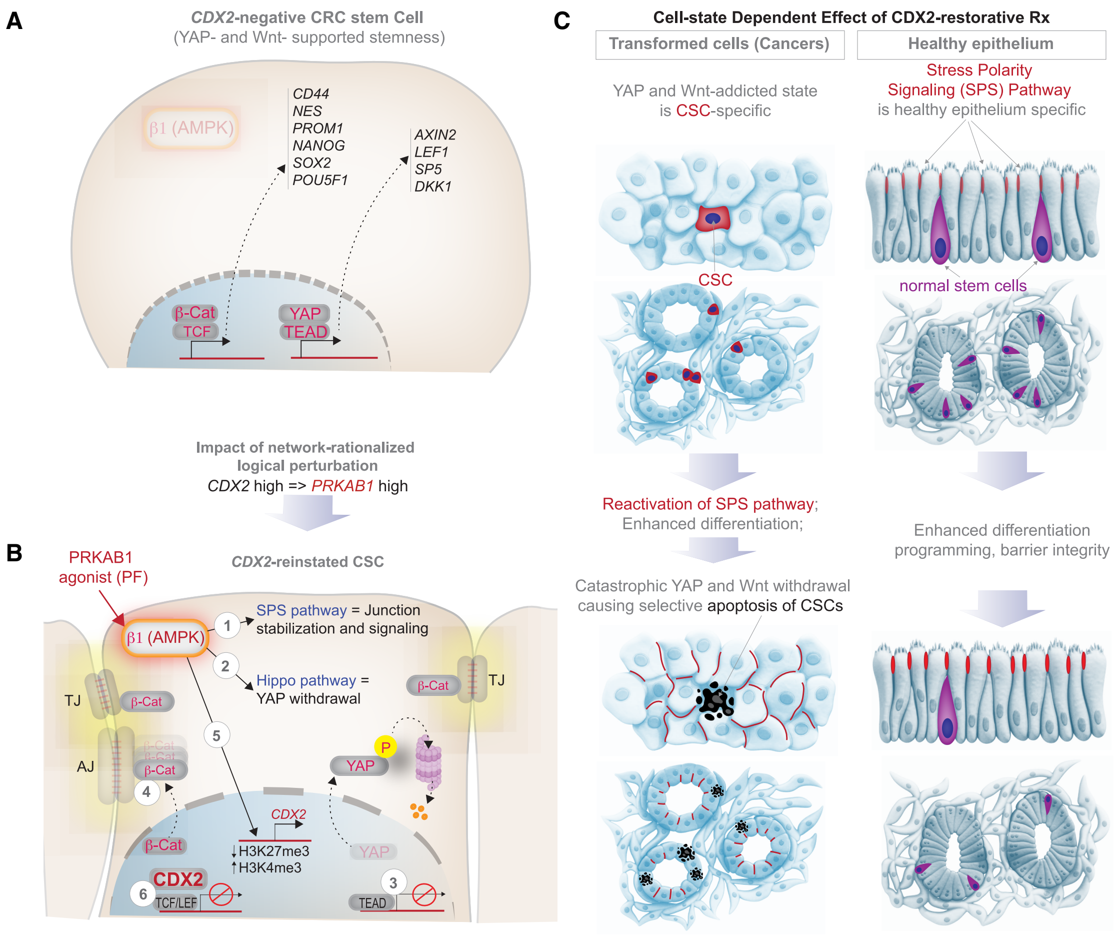

Figure 7. Summary and working model of how CDX2-reinstatement selectively targets CRC stem cells

(A) CRC stem cells, but not normal stem cells, are characterized by enhanced transcription of Wnt/β-catenin and YAP target genes that are established markers of CSCs. Both pathways are known to collaboratively enhance stemness in the colon.[104,105]

(B) PF treatment activates β1-specific AMPK, reactivates a tumor suppressor pathway (SPS pathway; 1), which attempts to restore tight- (TJ) and adherens (AJ) junctions in the setting of bioenergetic stress. Bioenergetic stress (AMPK activation) and junction restoration reshapes two junction-informed stemness programs, leading to their catastrophic withdrawal: *(i)* the Hippo-YAP pathway is *activated* (2), resulting in YAP phosphorylation followed by proteasomal degradation, and consequently, its nuclear exclusion (3) and withdrawal of the YAP/TEAD transcriptional program; *(ii)* junctional sequestration of β-catenin and consequently, its nuclear exclusion (4) and termination of the β-catenin·TCF/LEF transcriptional program. *CDX2*-reinstatement, and differentiation programs that accompanies the same, is likely via AMPK-dependent epigenetic events[106,107] (5). Once translated into protein, CDX2 is known to complete and displace β-catenin from TCF/LEF complexes[108] (6), which further accentuates the impact of the SPS pathway on the β-catenin·TCF/LEF transcriptional program.

(C) Schematic illustrates the cell state-specific impact of CDX2-reinstatement therapy and the impact of restoration of the stress polarity signaling (SPS) pathway. Pre- (top) and post- (bottom) treatment states of cancers (left) and healthy (right) epithelium are shown. Although activation of the SPS pathway enhances differentiation and junction formation in both cell states, the apoptotic fate is selectively seen in *CDX2*-low CSCs.

required for reanalysis of the data reported in this work paper is available from the lead contact upon request.

ACKNOWLEDGMENTS

We are grateful to the staffs of the UC San Diego HUMANOID™ Center of Research Excellence, for logistical support in conducting all studies on human PDOs. This work was supported by the National Institutes of Health (NIH) grants UG3 TR002968, UH3 TR002968, R01-CA238042, R01-CA100768, and R01-CA160911 (to P.G.) and VA Merit awards 1 I01 BX003856-01A1, I01CX002707-01A1 and 1 I01 BX004494-01 (to M.B.). S.S. was supported in part by an American Association of Immunologists (AAI) Intersect Fellowship Program for Computational Scientists and Immunologists. S.B. was supported by SERB International Research Experience Fellowship (SIR/2022/001374) by Govt. of India, during which she maintained her affiliation in the Department of Biotechnology, School of Biosciences and Technology, Vellore Institute of Technology, Vellore, Tamil Nadu 632014, India. Other sources of support include R01-AI155696, R01-AI141630, and UG3TR003355 (to P.G.); a Torrey Coast Foundation Award (to P.G.); Curebound Foundation award 23DG04; and by three Padres Pedal the Cause awards PTC2021, PTC2022, and PTC2024. P.G. was also supported by the Leona M. and Harry B. Helmsley Charitable Trust. Funders had no role in study design or conclusions. Authors acknowledge the instrumentation resources at the US San Diego Agilent Center of Excellence in Cellular Intelligence and Debashis Sahoo (UC San Diego) for providing access to computational resources.

AUTHOR CONTRIBUTIONS

S.S. and P.G. conceptualized the study; P.G. designed and analyzed all parts of the study; J.A., K.P., A.K.O., E.V., and C.T. acquired, expanded, biobanked, and conducted all experiments with PDOs and CRC cell lines; V.C. conducted IHC and immunofluorescence (IF) and hematoxylin and eosin (H&E) staining studies; C.R.E. conducted qPCR studies and assisted S.S., V.C., and P.G. on key parts of the study; S.B., E.M., K.P., S.S., and V.C. conducted IHC, IF, and H&E quantifications; S.T. built and validated the network model; S.S. and P.G. conducted all other computational, statistical, and bioinformatic analyses; S.A. and M.B. conducted the xenotransplantation studies; J.R.S. and J.Y. synthesized the pharmacophore; M.B., J.Y., and P.G. secured funding; S.S. and P.G. prepared all display items; P.G. wrote the first draft of the manuscript; and all authors edited and approved the final version.

DECLARATION OF INTERESTS

The authors no competing interests.

STAR★METHODS

Detailed methods are provided in the online version of this paper and include the following:

- KEY RESOURCES TABLE
- EXPERIMENTAL MODEL AND STUDY PARTICIPANT DETAILS
  - Cell culture
  - Animal model
  - Patient-derived organoid culture
  - Ethics statement
- METHOD DETAILS
  - Computational methods
  - Chemical synthesis of PRKAB1 agonist
  - Experimental methods
- QUANTIFICATION AND STATISTICAL ANALYSIS

SUPPLEMENTAL INFORMATION

Supplemental information can be found online at https://doi.org/10.1016/j.xcrm.2025.102421.

Received: February 5, 2024
Revised: July 24, 2025
Accepted: September 26, 2025
Published: October 20, 2025

REFERENCES

1. Stone, R.M., Maguire, M., Goldberg, M.A., Antin, J.H., Rosenthal, D.S., and Mayer, R.J. (1988). Complete remission in acute promyelocytic leukemia despite persistence of abnormal bone marrow promyelocytes during induction therapy: experience in 34 patients. Blood *71*, 690–696.
2. Kantarjian, H.M., Keating, M.J., Walters, R.S., Estey, E.H., McCredie, K. B., Smith, T.L., Dalton, W.T., Jr., Cork, A., Trujillo, J.M., and Freireich, E.J. (1986). Acute promyelocytic leukemia. M.D. Anderson Hospital experience. Am. J. Med. *80*, 789–797.
3. Cunningham, I., Gee, T.S., Reich, L.M., Kempin, S.J., Naval, A.N., and Clarkson, B.D. (1989). Acute promyelocytic leukemia: treatment results during a decade at Memorial Hospital. Blood *73*, 1116–1122.
4. Tallman, M.S., Andersen, J.W., Schiffer, C.A., Appelbaum, F.R., Feusner, J.H., Ogden, A., Shepherd, L., Willman, C., Bloomfield, C.D., Rowe, J.M., and Wiernik, P.H. (1997). All-trans-retinoic acid in acute promyelocytic leukemia. N. Engl. J. Med. *337*, 1021–1028. https://doi.org/10.1056/NEJM199710093371501.
5. Tallman, M.S., Andersen, J.W., Schiffer, C.A., Appelbaum, F.R., Feusner, J.H., Woods, W.G., Ogden, A., Weinstein, H., Shepherd, L., Willman, C., et al. (2002). All-trans retinoic acid in acute promyelocytic leukemia: long-term outcome and prognostic factor analysis from the North American Intergroup protocol. Blood *100*, 4298–4302. https://doi.org/10.1182/blood-2002-02-0632.
6. De Botton, S., Dombret, H., Sanz, M., Miguel, J.S., Caillot, D., Zittoun, R., Gardembas, M., Stamatoulas, A., Condé, E., Guerci, A., et al. (1998). Incidence, clinical features, and outcome of all trans-retinoic acid syndrome in 413 cases of newly diagnosed acute promyelocytic leukemia. Blood *92*, 2712–2718.
7. Testi, A.M., Biondi, A., Lo Coco, F., Moleti, M.L., Giona, F., Vignetti, M., Menna, G., Locatelli, F., Pession, A., Barisone, E., et al. (2005). GIMEMA-AIEOPAIDA protocol for the treatment of newly diagnosed acute promyelocytic leukemia (APL) in children. Blood *106*, 447–453. https://doi.org/10.1182/blood-2004-05-1971.
8. Fenaux, P., Chevret, S., Guerci, A., Fegueux, N., Dombret, H., Thomas, X., Sanz, M., Link, H., Maloisel, F., Gardin, C., et al. (2000). Long-term follow-up confirms the benefit of all-trans retinoic acid in acute promyelocytic leukemia. Leukemia *14*, 1371–1377.
9. Sanz, M.A., Martin, G., Rayon, C., Esteve, J., Gonzalez, M., Diaz-Mediavilla, J., Bolufer, P., Barragan, E., Terol, M.J., Gonzalez, J.D., et al. (1999). A modified AIDA protocol with anthracycline-based consolidation results in high antileukemic efficacy and reduced toxicity in newly diagnosed PML/RARalpha-positive acute promyelocytic leukemia. PETHEMA group. Blood *94*, 3015–3021.
10. Mandelli, F., Diverio, D., Avvisati, G., Luciano, A., Barbui, T., Bernasconi, C., Broccia, G., Cerri, R., Falda, M., Fioritoni, G., et al. (1997). Molecular remission in PML/RAR alpha-positive acute promyelocytic leukemia by combined all-trans retinoic acid and idarubicin (AIDA) therapy. Gruppo Italiano-Malattie Ematologiche Maligne dell'Adulto and Associazione Italiana di Ematologia ed Oncologia Pediatrica Cooperative Groups. Blood *90*, 1014–1021.
11. Bots, M., Verbrugge, I., Martin, B.P., Salmon, J.M., Ghisi, M., Baker, A., Stanley, K., Shortt, J., Ossenkoppele, G.J., Zuber, J., et al. (2014). Differentiation therapy for the treatment of t(8;21) acute myeloid leukemia using histone deacetylase inhibitors. Blood *123*, 1341–1352. https://doi.org/10.1182/blood-2013-03-488114.
12. Yen, K., Travins, J., Wang, F., David, M.D., Artin, E., Straley, K., Padyana, A., Gross, S., DeLaBarre, B., Tobin, E., et al. (2017). AG-221, a First-in-Class Therapy Targeting Acute Myeloid Leukemia Harboring Oncogenic IDH2 Mutations. Cancer Discov. *7*, 478–493. https://doi.org/10.1158/2159-8290.CD-16-1034.
13. Medema, J.P. (2017). Targeting the Colorectal Cancer Stem Cell. N. Engl. J. Med. *377*, 888–890. https://doi.org/10.1056/NEJMcibr1706541.
14. Butte, A.J., and Kohane, I.S. (1999). Unsupervised knowledge discovery in medical databases using relevance networks. In Proc. AMIA Symp., pp. 711–715.
15. Shameer, K., Readhead, B., and Dudley, J.T. (2015). Computational and experimental advances in drug repositioning for accelerated therapeutic stratification. Curr. Top. Med. Chem. *15*, 5–20.
16. Shen, Y., Alvarez, M.J., Bisikirska, B., Lachmann, A., Realubit, R., Pampou, S., Coku, J., Karan, C., and Califano, A. (2017). Systematic, network-based characterization of therapeutic target inhibitors. PLoS Comput. Biol. *13*, e1005599. https://doi.org/10.1371/journal.pcbi.1005599.
17. Margolin, A.A., Wang, K., Lim, W.K., Kustagi, M., Nemenman, I., and Califano, A. (2006). Reverse engineering cellular networks. Nat. Protoc. *1*, 662–671. https://doi.org/10.1038/nprot.2006.106.
18. van Someren, E.P., Wessels, L.F.A., Backer, E., and Reinders, M.J.T. (2002). Genetic network modeling. Pharmacogenomics *3*, 507–525. https://doi.org/10.1517/14622416.3.4.507.
19. Kusonmano, K. (2017). Gene Expression Analysis Through Network Biology: Bioinformatics Approaches. Adv. Biochem. Eng. Biotechnol. *160*, 15–32. https://doi.org/10.1007/10_2016_44.

20. Allocco, D.J., Kohane, I.S., and Butte, A.J. (2004). Quantifying the relationship between co-expression, co-regulation and gene function. BMC Bioinf. *5*, 18. https://doi.org/10.1186/1471-2105-5-18.

21. Arkin, A., and Ross, J. (1995). Statistical Construction of Chemical Reaction Mechanisms from Measured Time-Series. J. Phys. Chem. *99*, 970–979. https://doi.org/10.1021/j100003a020.

22. Jordan, I.K., Mariño-Ramírez, L., Wolf, Y.I., and Koonin, E.V. (2004). Conservation and coevolution in the scale-free human gene coexpression network. Mol. Biol. Evol. *21*, 2058–2070. https://doi.org/10.1093/molbev/msh222.

23. Lee, H.K., Hsu, A.K., Sajdak, J., Qin, J., and Pavlidis, P. (2004). Coexpression analysis of human genes across many microarray data sets. Genome Res. *14*, 1085–1094. https://doi.org/10.1101/gr.1910904.

24. Tavazoie, S., Hughes, J.D., Campbell, M.J., Cho, R.J., and Church, G.M. (1999). Systematic determination of genetic network architecture. Nat. Genet. *22*, 281–285. https://doi.org/10.1038/10343.

25. Butte, A.J., Tamayo, P., Slonim, D., Golub, T.R., and Kohane, I.S. (2000). Discovering functional relationships between RNA expression and chemotherapeutic susceptibility using relevance networks. Proc. Natl. Acad. Sci. USA *97*, 12182–12186. https://doi.org/10.1073/pnas.220392197.

26. Paik, S., Shak, S., Tang, G., Kim, C., Baker, J., Cronin, M., Baehner, F.L., Walker, M.G., Watson, D., Park, T., et al. (2004). A multigene assay to predict recurrence of tamoxifen-treated, node-negative breast cancer. N. Engl. J. Med. *351*, 2817–2826. https://doi.org/10.1056/NEJMoa041588.

27. Witten, D.M., and Tibshirani, R. (2010). Survival analysis with high-dimensional covariates. Stat. Methods Med. Res. *19*, 29–51. https://doi.org/10.1177/0962280209105024.

28. Alizadeh, A.A., Eisen, M.B., Davis, R.E., Ma, C., Lossos, I.S., Rosenwald, A., Boldrick, J.C., Sabet, H., Tran, T., Yu, X., et al. (2000). Distinct types of diffuse large B-cell lymphoma identified by gene expression profiling. Nature *403*, 503–511. https://doi.org/10.1038/35000501.

29. Zhao, H., Ljungberg, B., Grankvist, K., Rasmuson, T., Tibshirani, R., and Brooks, J.D. (2006). Gene expression profiling predicts survival in conventional renal cell carcinoma. PLoS Med. *3*, e13. https://doi.org/10.1371/journal.pmed.0030013.

30. Sahoo, D., Swanson, L., Sayed, I.M., Katkar, G.D., Ibeawuchi, S.R., Mittal, Y., Pranadinata, R.F., Tindle, C., Fuller, M., Stec, D.L., et al. (2021). Artificial intelligence guided discovery of a barrier-protective therapy in inflammatory bowel disease. Nat. Commun. *12*, 4246. https://doi.org/10.1038/s41467-021-24470-5.

31. Ghosh, P., Sinha, S., Katkar, G.D., Vo, D., Taheri, S., Dang, D., Das, S., and Sahoo, D. (2023). Machine learning identifies signatures of macrophage reactivity and tolerance that predict disease outcomes. EBioMedicine *94*, 104719. https://doi.org/10.1016/j.ebiom.2023.104719.

32. Sahoo, D., Seita, J., Bhattacharya, D., Inlay, M.A., Weissman, I.L., Plevritis, S.K., and Dill, D.L. (2010). MiDReG: a method of mining developmentally regulated genes using Boolean implications. Proc. Natl. Acad. Sci. USA *107*, 5732–5737. https://doi.org/10.1073/pnas.0913635107.

33. Inlay, M.A., Bhattacharya, D., Sahoo, D., Serwold, T., Seita, J., Karsunky, H., Plevritis, S.K., Dill, D.L., and Weissman, I.L. (2009). Ly6d marks the earliest stage of B-cell specification and identifies the branchpoint between B-cell and T-cell development. Genes Dev. *23*, 2376–2381. https://doi.org/10.1101/gad.1836009.

34. Chen, J.Y., Miyanishi, M., Wang, S.K., Yamazaki, S., Sinha, R., Kao, K.S., Seita, J., Sahoo, D., Nakauchi, H., and Weissman, I.L. (2016). Hoxb5 marks long-term haematopoietic stem cells and reveals a homogenous perivascular niche. Nature *530*, 223–227. https://doi.org/10.1038/nature16943.

35. Chan, C.K.F., Seo, E.Y., Chen, J.Y., Lo, D., McArdle, A., Sinha, R., Tevlin, R., Seita, J., Vincent-Tompkins, J., Wearda, T., et al. (2015). Identification and specification of the mouse skeletal stem cell. Cell *160*, 285–298. https://doi.org/10.1016/j.cell.2014.12.002.

36. Dimov, I.K., Lu, R., Lee, E.P., Seita, J., Sahoo, D., Park, S.M., Weissman, I.L., and Lee, L.P. (2014). Discriminating cellular heterogeneity using microwell-based RNA cytometry. Nat. Commun. *5*, 3451. https://doi.org/10.1038/ncomms4451.

37. Chan, C.K.F., Lindau, P., Jiang, W., Chen, J.Y., Zhang, L.F., Chen, C.C., Seita, J., Sahoo, D., Kim, J.B., Lee, A., et al. (2013). Clonal precursor of bone, cartilage, and hematopoietic niche stromal cells. Proc. Natl. Acad. Sci. USA *110*, 12643–12648. https://doi.org/10.1073/pnas.1310212110.

38. Seita, J., Sahoo, D., Rossi, D.J., Bhattacharya, D., Serwold, T., Inlay, M.A., Ehrlich, L.I.R., Fathman, J.W., Dill, D.L., and Weissman, I.L. (2012). Gene Expression Commons: an open platform for absolute gene expression profiling. PLoS One *7*, e40321. https://doi.org/10.1371/journal.pone.0040321.

39. Cheah, M.T., Chen, J.Y., Sahoo, D., Contreras-Trujillo, H., Volkmer, A.K., Scheeren, F.A., Volkmer, J.P., and Weissman, I.L. (2015). CD14-expressing cancer cells establish the inflammatory and proliferative tumor microenvironment in bladder cancer. Proc. Natl. Acad. Sci. USA *112*, 4725–4730. https://doi.org/10.1073/pnas.1424795112.

40. Volkmer, J.P., Sahoo, D., Chin, R.K., Ho, P.L., Tang, C., Kurtova, A.V., Willingham, S.B., Pazhanisamy, S.K., Contreras-Trujillo, H., Storm, T.A., et al. (2012). Three differentiation states risk-stratify bladder cancer into distinct subtypes. Proc. Natl. Acad. Sci. USA *109*, 2078–2083. https://doi.org/10.1073/pnas.1120605109.

41. Shin, K., Lim, A., Zhao, C., Sahoo, D., Pan, Y., Spiekerkoetter, E., Liao, J.C., and Beachy, P.A. (2014). Hedgehog signaling restrains bladder cancer progression by eliciting stromal production of urothelial differentiation factors. Cancer Cell *26*, 521–533. https://doi.org/10.1016/j.ccell.2014.09.001.

42. Sin, M.L.Y., Mach, K.E., Sinha, R., Wu, F., Trivedi, D.R., Altobelli, E., Jensen, K.C., Sahoo, D., Lu, Y., and Liao, J.C. (2017). Deep Sequencing of Urinary RNAs for Bladder Cancer Molecular Diagnostics. Clin. Cancer Res. *23*, 3700–3710. https://doi.org/10.1158/1078-0432.CCR-16-2610.

43. Sahoo, D., Wei, W., Auman, H., Hurtado-Coll, A., Carroll, P.R., Fazli, L., Gleave, M.E., Lin, D.W., Nelson, P.S., Simko, J., et al. (2018). Boolean analysis identifies CD38 as a biomarker of aggressive localized prostate cancer. Oncotarget *9*, 6550–6561. https://doi.org/10.18632/oncotarget.23973.

44. Bhamre, S., Sahoo, D., Tibshirani, R., Dill, D.L., and Brooks, J.D. (2009). Temporal changes in gene expression induced by sulforaphane in human prostate cancer cells. Prostate *69*, 181–190. https://doi.org/10.1002/pros.20869.

45. Ghosh, P., Campos, V.J., Vo, D.T., Guccione, C., Goheen-Holland, V., Tindle, C., Mazzini, G.S., He, Y., Alexandrov, L.B., Lippman, S.M., et al. (2022). AI-assisted discovery of an ethnicity-influenced driver of cell transformation in esophageal and gastroesophageal junction adenocarcinomas. JCI Insight *7*, e161334. https://doi.org/10.1172/jci.insight.161334.

46. Bonhomme, C., Duluc, I., Martin, E., Chawengsaksophak, K., Chenard, M.P., Kedinger, M., Beck, F., Freund, J.N., and Domon-Dell, C. (2003). The Cdx2 homeobox gene has a tumour suppressor function in the distal colon in addition to a homeotic role during gut development. Gut *52*, 1465–1471.

47. Silberg, D.G., Swain, G.P., Suh, E.R., and Traber, P.G. (2000). Cdx1 and cdx2 expression during intestinal development. Gastroenterology *119*, 961–971.

48. Chawengsaksophak, K., James, R., Hammond, V.E., Köntgen, F., and Beck, F. (1997). Homeosis and intestinal tumours in Cdx2 mutant mice. Nature *386*, 84–87.

49. Hryniuk, A., Grainger, S., Savory, J.G.A., and Lohnes, D. (2014). Cdx1 and Cdx2 function as tumor suppressors. J. Biol. Chem. *289*, 33343–33354.

CellPress
OPEN ACCESS

50. Sahoo, D., Dill, D.L., Gentles, A.J., Tibshirani, R., and Plevritis, S.K. (2008). Boolean implication networks derived from large scale, whole genome microarray datasets. Genome Biol. *9*, R157.
51. Dalerba, P., Kalisky, T., Sahoo, D., Rajendran, P.S., Rothenberg, M.E., Leyrat, A.A., Sim, S., Okamoto, J., Johnston, D.M., Qian, D., et al. (2011). Single-cell dissection of transcriptional heterogeneity in human colon tumors. Nat. Biotechnol. *29*, 1120–1127.
52. Dalerba, P., Sahoo, D., Paik, S., Guo, X., Yothers, G., Song, N., Wilcox-Fogel, N., Forgó, E., Rajendran, P.S., Miranda, S.P., et al. (2016). CDX2 as a Prognostic Biomarker in Stage II and Stage III Colon Cancer. N. Engl. J. Med. *374*, 211–222. https://doi.org/10.1056/NEJMoa1506597.
53. Levin, T.G., Powell, A.E., Davies, P.S., Silk, A.D., Dismuke, A.D., Anderson, E.C., Swain, J.R., and Wong, M.H. (2010). Characterization of the intestinal cancer stem cell marker CD166 in the human and mouse gastrointestinal tract. Gastroenterology *139*, 2072–2082.e5. https://doi.org/10.1053/j.gastro.2010.08.053.
54. Weichert, W., Knösel, T., Bellach, J., Dietel, M., and Kristiansen, G. (2004). ALCAM/CD166 is overexpressed in colorectal carcinoma and correlates with shortened patient survival. J. Clin. Pathol. *57*, 1160–1164.
55. Dalerba, P., Dylla, S.J., Park, I.K., Liu, R., Wang, X., Cho, R.W., Hoey, T., Gurney, A., Huang, E.H., Simeone, D.M., et al. (2007). Phenotypic characterization of human colorectal cancer stem cells. Proc. Natl. Acad. Sci. USA *104*, 10158–10163.
56. Derangère, V., Lecuelle, J., Lepage, C., Aoulad-Ben Salem, O., Allatessem, B.M., Ilie, A., Bouché, O., Phelip, J.M., Baconnier, M., Pezet, D., et al. (2022). Combination of CDX2 H-score quantitative analysis with CD3 AI-guided analysis identifies patients with a good prognosis only in stage III colon cancer. Eur. J. Cancer *172*, 221–230. https://doi.org/10.1016/j.ejca.2022.05.040.
57. Bennedsen, A.L.B., Cai, L., Hasselager, R.P., Özcan, A.A., Mohamed, K. B., Eriksen, J.O., Eiholm, S., Bzorek, M., Fiehn, A.M.K., Hviid, T.V.F., and Gögenur, I. (2022). An exploration of immunohistochemistry-based prognostic markers in patients undergoing curative resections for colon cancer. BMC Cancer *22*, 62. https://doi.org/10.1186/s12885-022-09169-0.
58. Azcue, P., Guerrero Setas, D., Encío, I., Ibáñez-Beroiz, B., Mercado, M., Vera, R., and Gómez-Dorronsoro, M.L. (2021). A Novel Prognostic Biomarker Panel for Early-Stage Colon Carcinoma. Cancers (Basel) *13*, 5909. https://doi.org/10.3390/cancers13235909.
59. den Uil, S.H., de Wit, M., Slebos, R.J.C., Delis-van Diemen, P.M., Sanders, J., Piersma, S.R., Pham, T.V., Coupé, V.M.H., Bril, H., Stockmann, H.B.A.C., et al. (2021). Quantitative analysis of CDX2 protein expression improves its clinical utility as a prognostic biomarker in stage II and III colon cancer. Eur. J. Cancer *144*, 91–100. https://doi.org/10.1016/j.ejca.2020.10.029.
60. Tarazona, N., Gimeno-Valiente, F., Gambardella, V., Huerta, M., Roselló, S., Zuniga, S., Calon, A., Carbonell-Asins, J.A., Fontana, E., Martinez-Ciarpaglini, C., et al. (2020). Detection of postoperative plasma circulating tumour DNA and lack of CDX2 expression as markers of recurrence in patients with localised colon cancer. ESMO Open *5*, e000847. https://doi.org/10.1136/esmoopen-2020-000847.
61. Hestetun, K.E., Aasebø, K., Rosenlund, N.B., Müller, Y., Dahl, O., and Myklebust, M.P. (2021). Mismatch repair phenotype determines the implications of tumor grade and CDX2 expression in stage II-III colon cancer. Mod. Pathol. *34*, 161–170. https://doi.org/10.1038/s41379-020-0634-9.
62. Asgari-Karchekani, S., Karimian, M., Mazoochi, T., Taheri, M.A., and Khamehchian, T. (2020). CDX2 Protein Expression in Colorectal Cancer and ItsCorrelation with Clinical and Pathological Characteristics, Prognosis, and Survival Rate of Patients. J. Gastrointest. Cancer *51*, 844–849. https://doi.org/10.1007/s12029-019-00314-w.
63. Ma, C., Olevian, D., Miller, C., Herbst, C., Jayachandran, P., Kozak, M.M., Chang, D.T., and Pai, R.K. (2019). SATB2 and CDX2 are prognostic biomarkers in DNA mismatch repair protein deficient colon cancer. Mod. Pathol. *32*, 1217–1231. https://doi.org/10.1038/s41379-019-0265-1.
64. Ben-Aharon, I., Goshen-Lago, T., Sternschuss, M., Morgenstern, S., Geva, R., Beny, A., Dror, Y., Steiner, M., Hubert, A., Idelevich, E., et al. (2019). Sidedness Matters: Surrogate Biomarkers Prognosticate Colorectal Cancer upon Anatomic Location. Oncologist *24*, e696–e701. https://doi.org/10.1634/theoncologist.2018-0351.
65. Hansen, T.F., Kjær-Frifeldt, S., Eriksen, A.C., Lindebjerg, J., Jensen, L.H., Sørensen, F.B., and Jakobsen, A. (2018). Prognostic impact of CDX2 in stage II colon cancer: results from two nationwide cohorts. Br. J. Cancer *119*, 1367–1373.
66. Pilati, C., Taieb, J., Balogoun, R., Marisa, L., De Reyniès, A., and Laurent-Puig, P. (2017). CDX2 prognostic value in stage II/III resected colon cancer is related to CMS classification. Ann. Oncol. *28*, 1032–1035.
67. Nolte, S., Zlobec, I., Lugli, A., Hohenberger, W., Croner, R., Merkel, S., Hartmann, A., Geppert, C.I., and Rau, T.T. (2017). Construction and analysis of tissue microarrays in the era of digital pathology: a pilot study targeting CDX1 and CDX2 in a colon cancer cohort of 612 patients. J. Pathol. Clin. Res. *3*, 58–70. https://doi.org/10.1002/cjp2.62.
68. Zhang, B.Y., Jones, J.C., Briggler, A.M., Hubbard, J.M., Kipp, B.R., Sargent, D.J., Dixon, J.G., and Grothey, A. (2017). Lack of caudal-type homeobox transcription factor 2 expression as a prognostic biomarker in metastatic colorectal cancer. Clin. Colorectal Cancer *16*, 124–128.
69. Tomasello, G., Barni, S., Turati, L., Ghidini, M., Pezzica, E., Passalacqua, R., and Petrelli, F. (2018). Association of CDX2 expression with survival in early colorectal cancer: a systematic review and meta-analysis. Clin. Colorectal Cancer *17*, 97–103.
70. Bruun, J., Sveen, A., Barros, R., Eide, P.W., Eilertsen, I., Kolberg, M., Pellinen, T., David, L., Svindland, A., Kallioniemi, O., et al. (2018). Prognostic, predictive, and pharmacogenomic assessments of CDX2 refine stratification of colorectal cancer. Mol. Oncol. *12*, 1639–1655. https://doi.org/10.1002/1878-0261.12347.
71. Baba, Y., Nosho, K., Shima, K., Freed, E., Irahara, N., Philips, J., Meyerhardt, J.A., Hornick, J.L., Shivdasani, R.A., Fuchs, C.S., and Ogino, S. (2009). Relationship of CDX2 loss with molecular features and prognosis in colorectal cancer. Clin. Cancer Res. *15*, 4665–4673.
72. Olsen, J., Eiholm, S., Kirkeby, L.T., Espersen, M.L.M., Jess, P., Gögenür, I., Olsen, J., and Troelsen, J.T. (2016). CDX2 downregulation is associated with poor differentiation and MMR deficiency in colon cancer. Exp. Mol. Pathol. *100*, 59–66.
73. Bruun, J., Sveen, A., Barros, R., Eide, P.W., Eilertsen, I., Kolberg, M., Pellinen, T., David, L., Svindland, A., Kallioniemi, O., et al. (2018). Prognostic, predictive, and pharmacogenomic assessments of CDX 2 refine stratification of colorectal cancer. Mol. Oncol. *12*, 1639–1655.
74. Kumar, N., Tsai, Y.H., Chen, L., Zhou, A., Banerjee, K.K., Saxena, M., Huang, S., Toke, N.H., Xing, J., Shivdasani, R.A., et al. (2019). The lineage-specific transcription factor CDX2 navigates dynamic chromatin to control distinct stages of intestine development. Development *146*, dev172189. https://doi.org/10.1242/dev.172189.
75. Fearon, E.R., and Huang, E.H. (2016). CDX2: Linking Cell and Patient Fates in Colon Cancer. Cell Stem Cell *18*, 168–169. https://doi.org/10.1016/j.stem.2016.01.011.
76. Fouqueray, P., Bolze, S., Dubourg, J., Hallakou-Bozec, S., Theurey, P., Grouin, J.M., Chevalier, C., Gluais-Dagorn, P., Moller, D.E., and Cusi, K. (2021). Pharmacodynamic effects of direct AMP kinase activation in humans with insulin resistance and non-alcoholic fatty liver disease: A phase 1b study. Cell Rep. Med. *2*, 100474. https://doi.org/10.1016/j.xcrm.2021.100474.
77. Ghosh, P. (2017). The stress polarity pathway: AMPK 'GIV'-es protection against metabolic insults. Aging (Albany NY) *9*, 303–314. https://doi.org/10.18632/aging.101179.
78. Ghosh, P., Swanson, L., Sayed, I.M., Mittal, Y., Lim, B.B., Ibeawuchi, S. R., Foretz, M., Viollet, B., Sahoo, D., and Das, S. (2020). The stress polarity signaling (SPS) pathway serves as a marker and a target in the leaky gut barrier: implications in aging and cancer. Life Sci. Alliance *3*, e201900481. https://doi.org/10.26508/lsa.201900481.

79. Aznar, N., Patel, A., Rohena, C.C., Dunkel, Y., Joosen, L.P., Taupin, V., Kufareva, I., Farquhar, M.G., and Ghosh, P. (2016). AMP-activated protein kinase fortifies epithelial tight junctions during energetic stress via its effector GIV/Girdin. eLife *5*, e20795. https://doi.org/10.7554/eLife.20795.

80. Gough, N.R. (2007). Maintaining Polarity in the Face of Energetic Stress. Sci. STKE *2007*, tw166. https://doi.org/10.1126/stke.3862007tw166.

81. Sahoo, D., Dill, D.L., Tibshirani, R., and Plevritis, S.K. (2007). Extracting binary signals from microarray time-course data. Nucleic Acids Res. *35*, 3705–3712. https://doi.org/10.1093/nar/gkm284.

82. Graule, J., Uth, K., Fischer, E., Centeno, I., Galván, J.A., Eichmann, M., Rau, T.T., Langer, R., Dawson, H., Nitsche, U., et al. (2018). CDX2 in colorectal cancer is an independent prognostic factor and regulated by promoter methylation and histone deacetylation in tumors of the serrated pathway. Clin. Epigenetics *10*, 120.

83. Boman, B.M., and Huang, E. (2008). Human colon cancer stem cells: a new paradigm in gastrointestinal oncology. J. Clin. Oncol. *26*, 2828–2838. https://doi.org/10.1200/JCO.2008.17.6941.

84. Kalisky, T., Rajendran, P.S., Sahoo, D., Sim, S., Okamoto, J., Miranda, S. P., Johnston, D.M., Clarke, M.F., Quake, S.R., and Dalerba, P. (2013). Analysis of human colon tissue cell composition using single-cell gene-expression PCR. J. Biomol. Tech.: J. Biochem. (Tokyo) *24*, S11.

85. Malta, T.M., Sokolov, A., Gentles, A.J., Burzykowski, T., Poisson, L., Weinstein, J.N., Kamińska, B., Huelsken, J., Omberg, L., Gevaert, O., et al. (2018). Machine Learning Identifies Stemness Features Associated with Oncogenic Dedifferentiation. Cell *173*, 338–354.e15. https://doi.org/10.1016/j.cell.2018.03.034.

86. Foucquier, J., and Guedj, M. (2015). Analysis of drug combinations: current methodological landscape. Pharmacol. Res. Perspect. *3*, e00149. https://doi.org/10.1002/prp2.149.

87. Wu, B., Tao, L., Yang, D., Li, W., Xu, H., and He, Q. (2020). Development of an Immune Infiltration-Related Eight-Gene Prognostic Signature in Colorectal Cancer Microenvironment. BioMed Res. Int. *2020*, 2719739. https://doi.org/10.1155/2020/2719739.

88. Yoshihara, K., Shahmoradgoli, M., Martínez, E., Vegesna, R., Kim, H., Torres-Garcia, W., Treviño, V., Shen, H., Laird, P.W., Levine, D.A., et al. (2013). Inferring tumour purity and stromal and immune cell admixture from expression data. Nat. Commun. *4*, 2612. https://doi.org/10.1038/ncomms3612.

89. Friedmann-Morvinski, D., and Verma, I.M. (2014). Dedifferentiation and reprogramming: origins of cancer stem cells. EMBO Rep. *15*, 244–253. https://doi.org/10.1002/embr.201338254.

90. Guinney, J., Dienstmann, R., Wang, X., de Reyniès, A., Schlicker, A., Soneson, C., Marisa, L., Roepman, P., Nyamundanda, G., Angelino, P., et al. (2015). The consensus molecular subtypes of colorectal cancer. Nat. Med. *21*, 1350–1356. https://doi.org/10.1038/nm.3967.

91. Isella, C., Terrasi, A., Bellomo, S.E., Petti, C., Galatola, G., Muratore, A., Mellano, A., Senetta, R., Cassenti, A., Sonetto, C., et al. (2015). Stromal contribution to the colorectal cancer transcriptome. Nat. Genet. *47*, 312–319. https://doi.org/10.1038/ng.3224.

92. Győrffy, B. (2024). Integrated analysis of public datasets for the discovery and validation of survival-associated genes in solid tumors. Innovation *5*, 100625. https://doi.org/10.1016/j.xinn.2024.100625.

93. Valladares-Ayerbes, M., Blanco-Calvo, M., Reboredo, M., Lorenzo-Patiño, M.J., Iglesias-Díaz, P., Haz, M., Díaz-Prado, S., Medina, V., Santamarina, I., Pértega, S., et al. (2012). Evaluation of the adenocarcinoma-associated gene AGR2 and the intestinal stem cell marker LGR5 as biomarkers in colorectal cancer. Int. J. Mol. Sci. *13*, 4367–4387. https://doi.org/10.3390/ijms13044367.

94. Li, Y., Yuan, Y., Yang, L., Chen, H., Zhang, X., Wen, T., Liao, W., Zhao, M., Zhao, Z., and Hu, Q. (2023). AT7867 Inhibits the Growth of Colorectal Cancer Stem-Like Cells and Stemness by Regulating the Stem Cell Maintenance Factor Ascl2 and Akt Signaling. Stem Cells Int. *2023*, 4199052. https://doi.org/10.1155/2023/4199052.

95. Jubb, A.M., Chalasani, S., Frantz, G.D., Smits, R., Grabsch, H.I., Kavi, V., Maughan, N.J., Hillan, K.J., Quirke, P., and Koeppen, H. (2006). Achaete-scute like 2 (ascl2) is a target of Wnt signalling and is upregulated in intestinal neoplasia. Oncogene *25*, 3445–3457. https://doi.org/10.1038/sj.onc.1209382.

96. Durinikova, E., Kozovska, Z., Poturnajova, M., Plava, J., Cierna, Z., Babelova, A., Bohovic, R., Schmidtova, S., Tomas, M., Kucerova, L., and Matuskova, M. (2018). ALDH1A3 upregulation and spontaneous metastasis formation is associated with acquired chemoresistance in colorectal cancer cells. BMC Cancer *18*, 848. https://doi.org/10.1186/s12885-018-4758-y.

97. Chen, G., She, X., Yin, Y., Ma, J., Gao, Y., Gao, H., Qin, H., and Fang, J. (2022). Targeting TM4SF1 exhibits therapeutic potential via inhibition of cancer stem cells. Signal Transduct. Target. Ther. *7*, 350. https://doi.org/10.1038/s41392-022-01177-7.

98. Tang, Q., Chen, J., Di, Z., Yuan, W., Zhou, Z., Liu, Z., Han, S., Liu, Y., Ying, G., Shu, X., and Di, M. (2020). TM4SF1 promotes EMT and cancer stemness via the Wnt/β-catenin/SOX2 pathway in colorectal cancer. J. Exp. Clin. Cancer Res. *39*, 232. https://doi.org/10.1186/s13046-020-01690-z.

99. Matsui, W.H. (2016). Cancer stem cell signaling pathways. Medicine (Baltim.) *95*, S8–S19. https://doi.org/10.1097/MD.0000000000004765.

100. Yang, L., Shi, P., Zhao, G., Xu, J., Peng, W., Zhang, J., Zhang, G., Wang, X., Dong, Z., Chen, F., and Cui, H. (2020). Targeting cancer stem cell pathways for cancer therapy. Signal Transduct. Target. Ther. *5*, 8. https://doi.org/10.1038/s41392-020-0110-5.

101. Gierut, J., Zheng, Y., Bie, W., Carroll, R.E., Ball-Kell, S., Haegebarth, A., and Tyner, A.L. (2011). Disruption of the mouse protein tyrosine kinase 6 gene prevents STAT3 activation and confers resistance to azoxymethane. Gastroenterology *141*, e1371. https://doi.org/10.1053/j.gastro.2011.06.071.

102. Liu, C., Pan, Z., Chen, Q., Chen, Z., Liu, W., Wu, L., Jiang, M., Lin, W., Zhang, Y., Lin, W., et al. (2021). Pharmacological targeting PTK6 inhibits the JAK2/STAT3 sustained stemness and reverses chemoresistance of colorectal cancer. J. Exp. Clin. Cancer Res. *40*, 297. https://doi.org/10.1186/s13046-021-02059-6.

103. Zhao, B., Li, L., Tumaneng, K., Wang, C.Y., and Guan, K.L. (2010). A coordinated phosphorylation by Lats and CK1 regulates YAP stability through SCF(beta-TRCP). Genes Dev. *24*, 72–85. https://doi.org/10.1101/gad.1843810.

104. Deng, F., Peng, L., Li, Z., Tan, G., Liang, E., Chen, S., Zhao, X., and Zhi, F. (2018). YAP triggers the Wnt/β-catenin signalling pathway and promotes enterocyte self-renewal, regeneration and tumorigenesis after DSS-induced injury. Cell Death Dis. *9*, 153. https://doi.org/10.1038/s41419-017-0244-8.

105. Park, H.W., Kim, Y.C., Yu, B., Moroishi, T., Mo, J.S., Plouffe, S.W., Meng, Z., Lin, K.C., Yu, F.X., Alexander, C.M., et al. (2015). Alternative Wnt Signaling Activates YAP/TAZ. Cell *162*, 780–794. https://doi.org/10.1016/j.cell.2015.07.013.

106. Kawai, H., Tomii, K., Toyooka, S., Yano, M., Murakami, M., Tsukuda, K., and Shimizu, N. (2005). Promoter methylation downregulates CDX2 expression in colorectal carcinomas. Oncol. Rep. *13*, 547–551.

107. Sun, X., Yang, Q., Rogers, C.J., Du, M., and Zhu, M.-J. (2017). AMPK improves gut epithelial differentiation and barrier function via regulating Cdx2 expression. Cell Death Differ. *24*, 819–831.

108. Guo, R.J., Funakoshi, S., Lee, H.H., Kong, J., and Lynch, J.P. (2010). The intestine-specific transcription factor Cdx2 inhibits beta-catenin/TCF transcriptional activity by disrupting the beta-catenin-TCF protein complex. Carcinogenesis *31*, 159–166. https://doi.org/10.1093/carcin/bgp213.

109. Zage, P.E., Huo, Y., Subramonian, D., Le Clorennec, C., Ghosh, P., and Sahoo, D. (2023). Identification of a novel gene signature for

CellPress
OPEN ACCESS

neuroblastoma differentiation using a Boolean implication network. Genes Chromosomes Cancer *62*, 313–331. https://doi.org/10.1002/gcc.23124.

110. Arafeh, R., Shibue, T., Dempster, J.M., Hahn, W.C., and Vazquez, F. (2025). The present and future of the Cancer Dependency Map. Nat. Rev. Cancer *25*, 59–73. https://doi.org/10.1038/s41568-024-00763-x.

111. Aasebø, K., Dragomir, A., Sundström, M., Mezheyeuski, A., Edqvist, P. H., Eide, G.E., Ponten, F., Pfeiffer, P., Glimelius, B., and Sorbye, H. (2020). CDX2: A Prognostic Marker in Metastatic Colorectal Cancer Defining a Better. Front. Oncol. *10*, 8. https://doi.org/10.3389/fonc.2020.00008.

112. Li, X., Sun, K., Liao, X., Gao, H., Zhu, H., and Xu, R. (2020). Colorectal carcinomas with mucinous differentiation are associated with high frequent mutation of KRAS or BRAF mutations, irrespective of quantity of mucinous component. BMC Cancer *20*, 400. https://doi.org/10.1186/s12885-020-06913-2.

113. Feng, Y., Bommer, G.T., Zhao, J., Green, M., Sands, E., Zhai, Y., Brown, K., Burberry, A., Cho, K.R., and Fearon, E.R. (2011). Mutant KRAS promotes hyperplasia and alters differentiation in the colon epithelium but does not expand the presumptive stem cell pool. Gastroenterology *141*, 1003–1013.e10. https://doi.org/10.1053/j.gastro.2011.05.007.

114. Brandt, R., Sell, T., Lüthen, M., Uhlitz, F., Klinger, B., Riemer, P., Giesecke-Thiel, C., Schulze, S., El-Shimy, I.A., Kunkel, D., et al. (2019). Cell type-dependent differential activation of ERK by oncogenic KRAS in colon cancer and intestinal epithelium. Nat. Commun. *10*, 2919. https://doi.org/10.1038/s41467-019-10954-y.

115. Aasebø, K., Dragomir, A., Sundström, M., Mezheyeuski, A., Edqvist, P.-H., Eide, G.E., Ponten, F., Pfeiffer, P., Glimelius, B., and Sorbye, H. (2020). CDX2: a prognostic marker in metastatic colorectal cancer defining a better BRAF mutated and a worse KRAS mutated subgroup. Front. Oncol. *10*, 8.

116. Slik, K., Turkki, R., Carpén, O., Kurki, S., Korkeila, E., Sundström, J., and Pellinen, T. (2019). CDX2 loss with microsatellite stable phenotype predicts poor clinical outcome in stage II colorectal carcinoma. Am. J. Surg. Pathol. *43*, 1473–1482.

117. Delhorme, J.-B., Bersuder, E., Terciolo, C., Vlami, O., Chenard, M.-P., Martin, E., Rohr, S., Brigand, C., Duluc, I., Freund, J.-N., and Gross, I. (2022). CDX2 controls genes involved in the metabolism of 5-fluorouracil and is associated with reduced efficacy of chemotherapy in colorectal cancer. Biomed. Pharmacother. *147*, 112630.

118. Takakura, Y., Hinoi, T., Oue, N., Sasada, T., Kawaguchi, Y., Okajima, M., Akyol, A., Fearon, E.R., Yasui, W., and Ohdan, H. (2010). CDX2 regulates multidrug resistance 1 gene expression in malignant intestinal epithelium. Cancer Res. *70*, 6767–6778.

119. Fontana, E., Meyers, J., Sobrero, A., Iveson, T., Shields, A.F., Taieb, J., Yoshino, T., Souglakos, I., Smyth, E.C., Lordick, F., et al. (2021). Early-Onset Colorectal Adenocarcinoma in the IDEA Database: Treatment Adherence, Toxicities, and Outcomes With 3 and 6 Months of Adjuvant Fluoropyrimidine and Oxaliplatin. J. Clin. Oncol. *39*, 4009–4019.

120. Sakamoto, N., Feng, Y., Stolfi, C., Kurosu, Y., Green, M., Lin, J., Green, M.E., Sentani, K., Yasui, W., McMahon, M., et al. (2017). BRAFV600E cooperates with CDX2 inactivation to promote serrated colorectal tumorigenesis. eLife *6*, e20331.

121. Tong, K., Pellón-Cárdenas, O., Sirihorachai, V.R., Warder, B.N., Kothari, O.A., Perekatt, A.O., Fokas, E.E., Fullem, R.L., Zhou, A., Thackray, J.K., et al. (2017). Degree of tissue differentiation dictates susceptibility to BRAF-driven colorectal cancer. Cell Rep. *21*, 3833–3845.

122. Felipe De Sousa, E.M., Wang, X., Jansen, M., Fessler, E., Trinh, A., De Rooij, L.P., De Jong, J.H., De Boer, O.J., Van Leersum, R., and Bijlsma, M.F. (2013). Poor-prognosis colon cancer is defined by a molecularly distinct subtype and develops from serrated precursor lesions. Nature medicine *19*, 614–618.

123. Coskun, M. (2014). The role of CDX2 in inflammatory bowel disease. Dan. Med. J. *61*, B4820.

124. Coskun, M., Olsen, A.K., Holm, T.L., Kvist, P.H., Nielsen, O.H., Riis, L.B., Olsen, J., and Troelsen, J.T. (2012). TNF-α-induced down-regulation of CDX2 suppresses MEP1A expression in colitis. Biochim. Biophys. Acta *1822*, 843–851.

125. Yin, F., Xie, H., Lai, J., Chen, Y., Dong, J., Zhang, X., and Liu, X. (2020). Double negativity for expression of YAP1 and CDX2 defines an aggressive type of colitis-associated cancer. Anticancer Res. *40*, 5411–5416.

126. Lourenço, F.C., and Winton, D.J. (2018). Metaplastic Cdx2-depleted cells can be very disruptive neighbors. J. Exp. Med. *215*, 725–727. https://doi.org/10.1084/jem.20180215.

127. Balbinot, C., Armant, O., Elarouci, N., Marisa, L., Martin, E., De Clara, E., Onea, A., Deschamps, J., Beck, F., Freund, J.N., and Duluc, I. (2018). The Cdx2 homeobox gene suppresses intestinal tumorigenesis through non-cell-autonomous mechanisms. J. Exp. Med. *215*, 911–926. https://doi.org/10.1084/jem.20170934.

128. Bae, J.M., Lee, T.H., Cho, N.-Y., Kim, T.-Y., and Kang, G.H. (2015). Loss of CDX2 expression is associated with poor prognosis in colorectal cancer patients. World J. Gastroenterol. *21*, 1457–1467.

129. Reggiani Bonetti, L., Lionti, S., Vitarelli, E., and Barresi, V. (2017). Prognostic significance of CDX2 immunoexpression in poorly differentiated clusters of colorectal carcinoma. Virchows Arch. *471*, 731–741.

130. Alarid-Escudero, F. (2021). CDX2 testing+ FOLFOX for CDX2-negative colon cancer patients cost-effective strategy. PharmacoEcon. Outcomes News *892*, 9–27.

131. Sayed, I.M., Suarez, K., Lim, E., Singh, S., Pereira, M., Ibeawuchi, S.R., Katkar, G., Dunkel, Y., Mittal, Y., Chattopadhyay, R., et al. (2020). Host engulfment pathway controls inflammation in inflammatory bowel disease. FEBS J. *287*, 3967–3988. https://doi.org/10.1111/febs.15236.

132. Sayed, I.M., El-Hafeez, A.A.A., Maity, P.P., Das, S., and Ghosh, P. (2021). Modeling colorectal cancers using multidimensional organoids. Adv. Cancer Res. *151*, 345–383. https://doi.org/10.1016/bs.acr.2021.02.005.

133. Sato, T., Vries, R.G., Snippert, H.J., van de Wetering, M., Barker, N., Stange, D.E., van Es, J.H., Abo, A., Kujala, P., Peters, P.J., and Clevers, H. (2009). Single Lgr5 stem cells build crypt-villus structures in vitro without a mesenchymal niche. Nature *459*, 262–265. https://doi.org/10.1038/nature07935.

134. Sato, T., Stange, D.E., Ferrante, M., Vries, R.G.J., Van Es, J.H., Van den Brink, S., Van Houdt, W.J., Pronk, A., Van Gorp, J., Siersema, P.D., and Clevers, H. (2011). Long-term expansion of epithelial organoids from human colon, adenoma, adenocarcinoma, and Barrett's epithelium. Gastroenterology *141*, 1762–1772. https://doi.org/10.1053/j.gastro.2011.07.050.

135. Sayed, I.M., Chakraborty, A., Abd El-Hafeez, A.A., Sharma, A., Sahan, A. Z., Huang, W.J.M., Sahoo, D., Ghosh, P., Hazra, T.K., and Das, S. (2020). The DNA Glycosylase NEIL2 Suppresses. Cells *9*, 1980. https://doi.org/10.3390/cells9091980.

136. Sayed, I.M., Sahan, A.Z., Venkova, T., Chakraborty, A., Mukhopadhyay, D., Bimczok, D., Beswick, E.J., Reyes, V.E., Pinchuk, I., Sahoo, D., et al. (2020). infection downregulates the DNA glycosylase NEIL2, resulting in increased genome damage and inflammation in gastric epithelial cells. J. Biol. Chem. *295*, 11082–11098. https://doi.org/10.1074/jbc.RA119.009981.

137. Edgar, R., Domrachev, M., and Lash, A.E. (2002). Gene Expression Omnibus: NCBI gene expression and hybridization array data repository. Nucleic Acids Res. *30*, 207–210.

138. Barrett, T., Wilhite, S.E., Ledoux, P., Evangelista, C., Kim, I.F., Tomashevsky, M., Marshall, K.A., Phillippy, K.H., Sherman, P.M., Holko, M., et al. (2013). NCBI GEO: archive for functional genomics data sets–update. Nucleic Acids Res. *41*, D991–D995. https://doi.org/10.1093/nar/gks1193.

139. Barrett, T., Troup, D.B., Wilhite, S.E., Ledoux, P., Rudnev, D., Evangelista, C., Kim, I.F., Soboleva, A., Tomashevsky, M., and Edgar, R. (2007). NCBI GEO: mining tens of millions of expression profiles–database and tools

update. Nucleic Acids Res. *35*, D760–D765. https://doi.org/10.1093/nar/gkl887.

140. Sahoo, D. (2012). The power of boolean implication networks. Front. Physiol. *3*, 276. https://doi.org/10.3389/fphys.2012.00276.

141. Love, M.I., Huber, W., and Anders, S. (2014). Moderated estimation of fold change and dispersion for RNA-seq data with DESeq2. Genome Biol. *15*, 550. https://doi.org/10.1186/s13059-014-0550-8.

142. Jassal, B., Matthews, L., Viteri, G., Gong, C., Lorente, P., Fabregat, A., Sidiropoulos, K., Cook, J., Gillespie, M., Haw, R., et al. (2020). The reactome pathway knowledgebase. Nucleic Acids Res. *48*, D498–D503. https://doi.org/10.1093/nar/gkz1031.

143. Avila Cobos, F., Alquicira-Hernandez, J., Powell, J.E., Mestdagh, P., and De Preter, K. (2020). Benchmarking of cell type deconvolution pipelines for transcriptomics data. Nat. Commun. *11*, 5650. https://doi.org/10.1038/s41467-020-19015-1.

144. Monaco, G., Lee, B., Xu, W., Mustafah, S., Hwang, Y.Y., Carré, C., Burdin, N., Visan, L., Ceccarelli, M., Poidinger, M., et al. (2019). RNA-Seq Signatures Normalized by mRNA Abundance Allow Absolute Deconvolution of Human Immune Cell Types. Cell Rep. *26*, 1627–1640.e7. https://doi.org/10.1016/j.celrep.2019.01.041.

145. Varghese, F., Bukhari, A.B., Malhotra, R., and De, A. (2014). IHC Profiler: an open source plugin for the quantitative evaluation and automated scoring of immunohistochemistry images of human tissue samples. PLoS One *9*, e96801. https://doi.org/10.1371/journal.pone.0096801.

146. Grabinger, T., Luks, L., Kostadinova, F., Zimberlin, C., Medema, J.P., Leist, M., and Brunner, T. (2014). Ex vivo culture of intestinal crypt organoids as a model system for assessing cell death induction in intestinal epithelial cells and enteropathy. Cell Death Dis. *5*, e1228. https://doi.org/10.1038/cddis.2014.183.

147. Smith, M.C.P., Luker, K.E., Garbow, J.R., Prior, J.L., Jackson, E., Piwnica-Worms, D., and Luker, G.D. (2004). CXCR4 regulates growth of both primary and metastatic breast cancer. Cancer Res. *64*, 8604–8612. https://doi.org/10.1158/0008-5472.CAN-04-1844.

STAR★METHODS

KEY RESOURCES TABLE

REAGENT or RESOURCE	SOURCE	IDENTIFIER
Antibodies		
Anti-CDX2 antibody	Abcam	Cat#76541, RRID: AB_1523334
AMPK β1 Polyclonal antibody	Proteintech	Cat#10308-1-AP, RRID: AB_513239
Anti-pSer^{245}-GIV antibody	21st Century Biochemicals (Marlboro, MA)	Customized
Anti p-YAP antibody	Cell Signaling Technology	Cat#13008, RRID: AB_2650553
YAP antibody	Santa Cruz	Cat#101199, RRID: AB_1131430
Anti-beta catenin	Santa Cruz	Cat#7963, RRID: AB_626807
Goat anti Rabbit IgG (H + L) Secondary Antibody, Alexa Fluor 647	Invitrogen	Cat#A-21245, RRID: AB_2535813
F(ab')2-Goat anti-Rabbit IgG (H + L) Cross-Absorbed Secondary Antibody, Alexa Fluor 594	Invitrogen	Cat#A-11072, RRID: AB_2534116
Chemicals, peptides, and recombinant proteins		
eTox red	Agilent	Cat#8711009
Guava Cell Cycle Reagent	Millipore Sigma	Cat#SKU4500-0220
Dead Cell Apoptosis Kits with Annexin V	Thermo Fisher Scientific	Cat# V13242
TRIzol™ Reagent	Life Technologies	Cat#15596018
Quick-RNA MiniPrep Kit	Zymo Research	Cat#R2052
qScript™ cDNA SuperMix	QuantaBio	Cat#101414
PowerUp™ SYBR™ Green Master Mix	Applied Biosciences	Cat#A25741
Direct-zol RNA Miniprep Kit	Zymo Research	Cat#R1051
Zinc Formalin Fixative	Sigma-Aldrich	Cat#Z2902
DMEM/High glucose with L-glutamine; without sodium pyruvate	Cytiva	Cat#SH30022.FS
Fetal Bovine Serum	Sigma-Aldrich	Cat#F8067-500ML
Penicillin Streptomycin 100X Solution	Cytiva	Cat#SV30010
Trypsin-EDTA (0.25%), phenol red	Fisher Scientific	Cat#25-200-114
Dulbecco's Phosphate Buffered Saline (DPBS), 1X, without calcium, magnesium	Cytiva	Cat#16750-076
Matrigel	Corning	Cat#354234
Prochlorperazine dimaleate salt	Selleck Chemicals	Cat#S4631
Mayer's hematoxylin	BioGenex	Cat#HK1005K-GP
Two-component DAB brown	BioGenex	Cat#HK542XAKE-GPE
Collagenase I	GIBCO	Cat#17100-017
DNase I	Zymo Research	Cat#E1011-A
Human TruStain FcX™	BioLegend	Cat#422302
Poly-L-lysine coated slides	Fisher Scientific	Cat#63410-02
3% hydrogen peroxide	Fisher Scientific	Cat#S25359
Fluid Thioglycollate Medium	BD Difco	Cat#225650
2% paraformaldehyde	Electron Microscopy Services	Cat#15700
0.2% gluteraldehyde	Electron Microscopy Services	Cat#16200
0.1% Probumin® BSA	Millipore Sigma	Cat#820452
2% gelatin	Sigma-Aldrich	Cat# ES-006-B
Protease inhibitor cocktail	Roche Diagnostics	Cat#5056489001
Glutathione Sepharose beads	Sigma-Aldrich	Cat#17-0756-04
RPMI 1640 medium	Thermo Scientific	Cat#11875093

(*Continued on next page*)

REAGENT or RESOURCE	SOURCE	IDENTIFIER
Continued		
Gibco TrypLE express enzyme (1X)	Thermo Fisher Scientific	Cat#12563011
Intestigro^{TM}	HUMANOID	Cat#HUM2019-125
Dimethyl sulfoxide (DMSO)	Sigma-Aldrich	Cat#D2650-100ML
Thiazolyl Blue Tetrazolium Bromide	Sigma-Aldrich	Cat#M5655-1G
Sodium Dodecyl Sulfate	Fisher Scientific	Cat#BP166-500
Dead Cell Apoptosis Kit with Annexin V Alexa Flour 488 & Propidium Iodide (PI)	Thermo Fischer Scientific	Cat#V13245
1,4-Dibromobenzene	Fisher Scientific	Cat#AC112732500
Tetrahydrofuran (THF, anhydrous)	Fisher Scientific	Cat#T425-1
n-Butyllithium (n-BuLi, 2.5 M in hexane)	Fisher Scientific	Cat#AC213351000
Cyclobutanone	Fisher Scientific	Cat#AC110930250
Ammonium chloride (NH_4Cl, saturated aqueous)	Fisher Scientific	Cat#A2037300G
Ethyl acetate (EtOAc)	Fisher Scientific	Cat#AA31344M4
Brine (saturated NaCl solution)	Fisher Scientific	Cat#S271-10
Magnesium sulfate ($MgSO_4$)	Fisher Scientific	Cat#M65-3
Sodium tert-butoxide	Fisher Scientific	Cat#S0450100G
Potassium acetate	Fisher Scientific	Cat#P171-500
XPhos Pd G2 (palladium catalyst)	Sigma-Aldrich	Cat#741825-1G
XPhos (ligand)	Sigma-Aldrich	Cat#638064-1G
Tetrahydroxydiboron	Sigma-Aldrich	Cat#754242-25G
Argon gas	Matheson Tri-Gas	SKU: AR 200
5-Bromo-6-chloro-1H-indole	Combi-Blocks	Cat# QB-9262
4-Dimethylaminopyridine (DMAP)	Fisher Scientific	Cat#D1450100G
Pyridine	Fisher Scientific	Cat#P368-500
Trichloroacetyl chloride	Fisher Scientific	Cat#AAB2167518
Sodium methoxide (25% in methanol)	Fisher Scientific	Cat#AA46585AP
2-Methyltetrahydrofuran (2-MeTHF)	Fisher Scientific	Cat#AC396621000
Potassium carbonate (K_2CO_3, aqueous)	Fisher Scientific	Cat#P208-500
Sodium hydroxide (NaOH, 3 M aqueous)	Fisher Scientific	Cat#S318-1
Hydrochloric acid (HCl, 1 M aqueous)	Fisher Scientific	Cat#A144-212
Acetonitrile	Fisher Scientific	Cat#A998-4
Dichloromethane (DCM)	Fisher Scientific	Cat#D37-20
Trifluoroacetic acid (TFA, 0.1% in water for HPLC)	Fisher Scientific	Cat#AA44630AP
Deposited data		
RNA Sequencing: Induction of CDX2 as a differentiation therapy in colorectal cancers [cell lines]	This study	GEO: GSE237624
RNA Sequencing: Induction of CDX2 as a differentiation therapy in colorectal cancers [xenograft]	This study	GEO: GSE237625
RNA sequencing: Induction of CDX2 as a differentiation therapy in colorectal cancers [CRC organoids]	This study	GEO: GSE237623
Experimental models: Cell lines		
HCT-116	ATCC	CCL-247
Sw480	ATCC	CCL-228
DLD1	ATCC	CCL-221
Caco2	ATCC	HTB-37

(*Continued on next page*)

Continued

REAGENT or RESOURCE	SOURCE	IDENTIFIER
Experimental models: Organisms/strains		
Athymic nude male mice	Jackson Laboratory	002019
Patient-Derived Model-255	ATCC	HCM-CSHL-0245-C18-A
Patient-Derived Model-2	ATCC	HCM-CSHL-0057-C18
Patient-Derived Model-4	ATCC	HCM-CSHL-0060-C18
Patient-Derived Model-50	ATCC	HCM-SANG-0273-C18
Patient-Derived Model-255	ATCC	HCM-CSHL-0245-C18-A
Patient-Derived Model-275	ATCC	HCM-CSHL-0245-C18-B
Patient-Derived Model-1	ATCC	HCM-CSHL-0056-C18
Patient-Derived Model-279	ATCC	HCM-CSHL-0384-D37
Patient-Derived Model-185	ATCC	HCM-CSHL-0238-C18
Patient-Derived Model-191	ATCC	HCM-CSHL-0246-C19
Patient-Derived Model-9	ATCC	HCM-CSHL-0065-C20
Patient-Derived Model-264	ATCC	HCM-CSHL-0382-C19
Patient-Derived Model-94	ATCC	HCM-CSHL-0141-C18
Patient-Derived Model-356	ATCC	HCM-CSHL-0257-C18
Patient-Derived Model-7	ATCC	HCM-CSHL-0063-C18
Patient-Derived Model-257	ATCC	HCM-CSHL-0248-C19
Patient-Derived Model-363	ATCC	HCM-CSHL-0426-C18
Patient-Derived Model-276	ATCC	HCM-CSHL-0461-D12
Patient-Derived Model-277	ATCC	HCM-CSHL-0247-C18
Patient-Derived Model-359	ATCC	HCM-CSHL-0317-C18
Patient-Derived Model-5	ATCC	HCM-CSHL-0061-C18
Patient-Derived Model-103	ATCC	HCM-CSHL-0164-C20
Patient-Derived Model-8	ATCC	HCM-CSHL-0064-C18
Patient-Derived Model-95	ATCC	HCM-CSHL-0142-C18
Human colon-derived healthy organoids	UC San Diego HUMANOID^{TM} Center	N/A
Software and algorithms		
ImageJ	National Institute of Health	https://imagej.net/Welcome
Prism 9	GraphPad	https://www.graphpad.com/scientific-software/prism/
Molsoft	Molsoft, LLC	https://www.molsoft.com/index.html
LAS-X	Leica	www.leica-microsystems.com/products/microscope-software/p/leica-las-x-ls
Illustrator	Adobe	https://www.adobe.com/products/illustrator.html
ImageStudio Lite	LI-COR	https://www.licor.com/bio/image-studio-lite/
ClueGO	Cytoscape	https://academic.oup.com/bioinformatics/article/25/8/1091/324247
NetworkX	Python	https://networkx.org
NumPy	Python	https://numpy.org/
Pandas	Python	https://pandas.pydata.org/
Seaborn	Python	https://seaborn.pydata.org/
Gephi	Gephi	https://gephi.org
Photoshop	Adobe	https://www.adobe.com/products/photoshop.html
FlowJo	Flow Jo V10, BD BioSciences	RRID:SCR_008520

(*Continued on next page*)

Continued		
REAGENT or RESOURCE	SOURCE	IDENTIFIER
BioTek Gen5	Agilent	https://www.agilent.com/en/product/cell-analysis/cell-imaging-microscopy/cell-imaging-microscopy-software/biotek-gen5-software-for-imaging-microscopy-1623226
Code	Custom computer code	https://github.com/sinha7290/Prodiff
Other		
6-well Tissue Culture Plate	Genesee Scientific	Cat# 25-105
12-well Tissue Culture Plate	CytoOne	Cat# CC7682-7512
Transwell Inserts (6.5 mm, 0.4 μm pore size)	Corning	Cat# 3470
Cell Scraper	Millipore Sigma	Cat# C5981-100EA
Millicell EZ Slide 8-Well Chamber	Millipore Sigma	Cat# PEZGS0816
Trypan Blue Stain	Invitrogen	Cat# T10282
70 μm Cell Strainer	Thermo Fisher Scientific	Cat# 22-363-548
Noyes Spring Scissors - Angled	Fine Science Tools	Cat# 15013-12
gentleMACS^{TM} C Tubes	Miltenyi Biotec	Cat# 130-093-237
DAPI	Invitrogen	Cat# D1306; RRID: AB_2629482
Phalloidin, Alexa Fluor 594	Invitrogen	Cat# A12381; RRID: AB_2315633
Propidium Iodide	Invitrogen	Cat# V-13245 B
Countess II Automated Cell Counter	Thermo Fisher Scientific	AMQAX1000
Epithelial Volt-Ohm (TEER) Meter	Millipore	MERS00002
Automated TEER measurement system [REMS AutoSampler]	World Precision Instruments (WPI)	N/A
MESO QuickPlex SQ 120	MSD	N/A
Leica TCS SPE Confocal	Leica Microsystems	TCS SPE
Power Pressure Cooker XL	Tristar Products	N/A
Canon Rebel XS DLSR	Canon	N/A
MiniAmp Plus Thermal Cycler	Applied Biosystems	Cat# A37835
QuantStudio5	Applied Biosystems	Cat# A28140 RRID: SCR_020240
Light Microscope (brightfield images)	Carl Zeiss LLC	Axio Observer, Inverted; 491917-0001-000
Fisherbrand^{TM} 150 Handheld Homogenizer	Fisher Scientific	Cat# 15340168
Spark 20M Multimode Microplate Reader	Tecan	N/A
NanoQuant Infinite M200	Tecan	N/A
Guava® easyCyte Benchtop Flow Cytometer	Millipore	Guava easyCyte 6 2L
MESO QuickPlex instrument	Mesoscale Discovery Inc.	SQ 120
gentleMACS^{TM} Dissociator	Miltenyi Biotec	Cat# 130-093-235
Transwell Inserts (6.5 mm, 0.4 μm pore size)	Corning	Cat# 3470
Cell Scraper	Millipore Sigma	Cat# C5981-100EA
Millicell EZ Slide 8-Well Chamber	Millipore Sigma	Cat# PEZGS0816
Trypan Blue Stain	Invitrogen	Cat# T10282
70 μm Cell Strainer	Thermo Fisher Scientific	Cat# 22-363-548
Noyes Spring Scissors - Angled	Fine Science Tools	Cat# 15013-12
16% Paraformaldehyde	Electron Microscopy Sciences	Cat#15710
ProLong Glass Antifade Mountant	Thermo Fisher Scientific	Cat#P36984
2.5% Normal Goat Serum	Vector Laboratories	Cat#S-1012-50
2.5% Normal Horse Serum	Vector Laboratories	Cat#S-2012-50

EXPERIMENTAL MODEL AND STUDY PARTICIPANT DETAILS

Cell culture

All cell lines used in this study were obtained from American Type Culture Collection (ATCC). Cell line identity was authenticated using short tandem repeat (STR) profiling, and all lines were routinely tested for mycoplasma contamination using commercially available PCR-based kits. STR profiles of commercial cell lines were cross-referenced against established databases (ATCC, RIKEN, JCRB, DSMZ) to confirm identity. Only validated, mycoplasma-free cells were expanded and used for experiments.

HCT-116

This human colon cancer cell line was obtained from the ATCC and maintained in Roswell Park Memorial Institute 1640 (RPMI-1640) medium (Gibco-BRL). The medium was supplemented with 10% fetal calf serum (Hyclone), 1% L-Glutamine, and 1% penicillin/streptomycin (Gibco-BRL). The cells were incubated at 37°C in a 5% CO2 incubator and were routinely passaged at a dilution of 1:5 to 1:10.

SW480

This human colon cancer cell line was obtained from the ATCC and maintained in DMEM/F12, supplemented with 10% FBS. The cells were incubated at 37°C in a 5% CO2 incubator and were routinely passaged at a dilution of 1:2 to 1:5.

DLD1

This human colon cancer cell was obtained from the ATCC and were cultured using RMPI media containing 10% FBS. The cells were incubated at 37°C in a 5% CO2 incubator and were routinely passaged at a dilution of 1:5 to 1:10.

Caco2

This human CRC cell line was obtained from the ATCC and was cultured in high glucose DMEM/F12 supplemented with 10% FBS and 1% penicillin/streptomycin (Cytiva). The cells were incubated at 37°C in a 5% CO2 incubator and were routinely passaged at a dilution of 1:3 to 1:8.

Animal model

Athymic nude male mice ages 4–6 weeks purchased from Jackson Laboratories (Bar Harbor, ME) were utilized for this study. Mice were maintained in a barrier facility with high-efficiency particulate air filtration and fed an autoclaved laboratory diet. Prior to surgical procedures, mice were anesthetized with an intraperitoneal injection of ketamine and xylazine reconstituted in phosphate-buffered saline (PBS). When the study concluded or if tumor burden became too large, defined as tumor volume >1500 cm^3, mice were euthanized with CO2 inhalation followed by cervical dislocation. This study was carried out in strict accordance with the recommendations in the Guide for the Care and Use of Laboratory Animals of the National Institutes of Health. All animal studies were approved by the San Diego Veterans Administration Medical Center Institutional Animal Care and Use Committee (protocol A17-020).

Tumor establishment with cell injection

Subcutaneous injection of HCT-116 cells (1 x 10^6) reconstituted in PBS and Matrigel Matrix (Corning, NY) was performed on the bilateral shoulders and flanks of nude mice. Tumors were allowed to grow until 50 mm^3 in diameter. The tumors were then resected and divided into 1 mm^3 pieces for subcutaneous implantation.

Tumor establishment with tumor implantation

Subcutaneous models were established by surgical implantation of 1 mm^3 HCT-116 tumor fragment into the bilateral flank of nude mice. A small incision was made on the back of the mice and tumor fragment was directly implanted in the bilateral flank. The skin was closed with 6-0 nylon suture (Ethicon Inc., Sommerville. NJ). Tumors were allowed to grow until 150 mm^3 in diameter.

Patient-derived organoid culture

Twenty-three human colorectal cancer (CRC) patient-derived model (PDM) organoids were acquired from the Human Cancer Models Initiative (HCMI) catalog of the ATCC. Five healthy colon PDOs were used from the biobank of the UC San Diego HUMANOID Center of Research Excellence at the University of California, San Diego, USA.[131] Organoids were cultured and passaged according to previously described methods.[78,131–136] For maintenance, organoids were cultured in Matrigel in 12- or 24-well tissue culture plates incubated at 37°C, 5% CO_2. Organoids were provided 50% L-WRN-conditioned medium supplemented with differentiation inhibitors (Intestigrow; Cat#: HUM2019-125, HUMANOID, San Diego, CA). Organoids were subcultured every 7–14 days, dissociated by non-enzymatic digestion in TrypLE and passaged to achieve the desired density. All relevant demographic, clinical and tumor mutation related information is provided in Table S3. The study does not involve sex- or gender-specific hypotheses and is not restricted to one sex or gender.

Ethics statement

All animal studies were conducted in strict accordance with the NIH *Guide for the Care and Use of Laboratory Animals* and were approved by the Institutional Animal Care and Use Committee of the San Diego Veterans Administration Medical Center (protocol A17-020; PI Bouvet). CRC PDOs were obtained from the ATCC, a nonprofit global biorepository and distributor of organoid models curated under the Human Cancer Models Initiative (HCMI) to enhance reproducibility. Deidentified case-associated data, including molecular characterizations and tissue origins, were used in this study; harmonized datasets are publicly accessible via the NCI's

Genomic Data Commons. Healthy colon PDOs were derived from individuals undergoing routine colonoscopy for cancer screening at UC San Diego, with informed consent under an IRB-approved protocol (#190105; PI Ghosh).

METHOD DETAILS

Computational methods

Data processing

We built a human colon tissue database (n = 1,911) derived from the previously published "Human Colon Global Database," refined by filtering for EpCAM and Albumin expression and restricted to the Affymetrix Human U133 Plus 2.0 platform (GPL570). The dataset was obtained from the NCBI Gene Expression Omnibus (GEO) repository,[137–139] as described previously,[52] and was expanded by including 68 additional adenoma samples and FACS-purified human colon crypt samples (GSE31255). This dataset contained experiments from 23 independent NCBI-GEO data-series (GSEs) from the same platform (GPL570). A list of the 23 NCBI-GEO GSEs contained within the "Human colon tissue database" is provided in (Table S1). Then, the dataset was prepared for Boolean analysis by selecting genes that had the best dynamic range (identified by the biggest percentile range between the 10th and 90th percentile of low and high expression value based on the *StepMiner*[81] threshold for each gene in this dataset). All training and validation dataset (Table S1) were downloaded from the NCBI GEO website.[137–139] All gene expression datasets (Table S1) were processed separately using the Hegemon data analysis framework.[40,51,52] We did not combine datasets that belong to two different platforms.

Boolean analysis

Boolean logic is a simple mathematical relationship of two values, i.e., high/low, 1/0, or positive/negative. The Boolean analysis of gene expression data requires first the conversion of expression levels into two possible values. Here, *StepMiner*[81] algorithm is used to perform Boolean analysis of gene expression data.[7] StepMiner is an algorithm designed to detect stepwise transitions in time-series gene expression data.[81] It fits step functions to expression profiles by identifying the sharpest changes in signal, corresponding to gene expression switching events. The algorithm evaluates all possible step positions and calculates the average expression on either side of each step to define constant segments. An adaptive regression approach is then used to select the step position that minimizes the sum of squared errors between the observed and fitted data. The selected step is used as the *StepMiner* threshold. *This* threshold is used to convert gene expression values into Boolean values. A noise margin of 2-fold change is applied around the threshold to determine intermediate values, and these values are ignored during Boolean analysis. Finally, a regression test statistic is computed to assess the significance of the identified step transition as follows:

$$F\ stat = \frac{\sum_{i=1}^{n} (\widehat{X}_i - \overline{X})^2 \Big/ (m-1)}{\sum_{i=1}^{n} (X_i - \widehat{X}_i)^2 \Big/ (n-m)}$$

where X_i for $i = 1$ to n are the values, $\widehat{X}_i$ for $i = 1$ to n are fitted values. m is the degrees of freedom used for the adaptive regression analysis. $\overline{X}$ is the average of all the values: $\overline{X} = \frac{1}{n} * \sum_{j=1}^{n} X_j$. For a step position at k, the fitted values $\widehat{X}_I$ are computed by using $\frac{1}{k} * \sum_{j=1}^{n} X_j$ for $i = 1$ to k and $\frac{1}{(n-k)} * \sum_{j=k+1}^{n} X_j$ for $i = k+1$ to n.

In a scatterplot, there are four possible quadrants based on Boolean values: (low, low), (low, high), (high, low), (high, high). In all our experimental settings, we applied the same logic to classify samples into CDX2-high and CDX2-low groups based on the dynamic range of CDX2 expression levels.

Invariant Boolean implication relationships. A Boolean implication relationship (BIR) is observed if any one of the four possible quadrants or two diagonally opposite quadrants are sparsely populated. Based on this rule, there are six different kinds of BIRs. Two of them are symmetric: equivalent (corresponding to the highly positively correlated genes), opposite (corresponding to the highly negatively correlated genes). Four of the Boolean relationships are asymmetric, and each corresponds to one sparse quadrant: (low => low), (high => low), (low => high), (high => high). BooleanNet statistics (Equations listed below) is used to assess the sparsity of a quadrant and the significance of the BIRs.[32,50] Given a pair of genes A and B, four quadrants are identified by using the *StepMiner* thresholds on A and B by ignoring the Intermediate values defined by the noise margin of 2-fold change (±0.5 around *StepMiner* threshold). Number of samples in each quadrant are defined as a_{00}, a_{01}, a_{10}, and a_{11}. Total number of samples where gene expression values for A and B are low is computed using following equations.

$$nA_{low} = (a_{00} + a_{01}), nB_{low} = (a_{00} + a_{10})$$

Total number of samples considered is computed using following equation.

$$total = a_{00} + a_{01} + a_{10} + a_{11}$$

Expected number of samples in each quadrant is computed by assuming independence between A and B. For example, expected number of samples in the bottom left quadrant $e_{00} = \widehat{n}$ is computed as probability of A low (($a_{00} + a_{01}$)/total) multiplied by probability of B low (($a_{00} + a_{10}$)/total) multiplied by total number of samples.

CellPress
OPEN ACCESS

Following equation is used to compute the expected number of samples for the low-low quadrant.

$$n = a_{ij}, \hat{n} = (nA_{low} / total * nB_{low} / total) * total$$

To check whether a quadrant (here low-low) is sparse, a statistical test for ($e_{00} > a_{00}$) or ($\hat{n} > n$) is performed by computing S_{00} and p_{00} using following equations. A quadrant is considered sparse if S_{00} is high ($\hat{n} > n$) and p_{00} is small.

$$S_{ij} = \frac{\hat{n} - n}{\sqrt{\hat{n}}}$$

$$p_{00} = \frac{1}{2}\left(\frac{a_{00}}{(a_{00}+a_{01})} + \frac{a_{00}}{(a_{00}+a_{10})}\right)$$

A threshold of S_{00} > sthr and p_{00} < pthr to check sparse quadrant. A BIR is identified when a sparse quadrant is discovered using following equation.

$$\textit{Boolean Implication} = (S_{ij} > \text{sthr}, p_{ij} < \text{pthr})$$

A relationship is called Boolean equivalent if top-left and bottom-right quadrants are sparse.

$$\text{Equivalent} = (S_{01} > sthr, P_{01} < pthr, S_{10} > sthr, P_{10} < pthr)$$

Boolean opposite relationships have sparse top-right (a_{11}) and bottom-left (a_{00}) quadrants.

$$\textit{Opposite} = (S_{00} > sthr, P_{00} < pthr, S_{11} > sthr, P_{11} < pthr)$$

Boolean equivalent and opposite are symmetric relationship because the relationship from A to B is same as from B to A. Asymmetric relationship forms when there is only one quadrant sparse (A low => B low: top-left; A low => B high: bottom-left; A high=> B high: bottom-right; A high => B low: top-right). These relationships are asymmetric because the relationship from A to B is different from B to A. For example, A low => B low and B low => A low are two different relationships.

A low => B high is discovered if bottom-left (a_{00}) quadrant is sparse and this relationship satisfies following conditions.

$$A\ low = > B\ high = (S_{00} > sthr, P_{00} < pthr)$$

Similarly, A low => B low is identified if top-left (a_{01}) quadrant is sparse.

$$A\ low = > B\ low = (S_{01} > sthr, P_{01} < pthr)$$

A high => B high Boolean implication is established if bottom-right (a_{10}) quadrant is sparse as described below.

$$A\ high = > B\ high = (S_{10} > sthr, P_{10} < pthr)$$

Boolean implication A high => B low is found if top-right (a_{11}) quadrant is sparse using following equation.

$$A\ high = > B\ low = (S_{11} > sthr, P_{11} < pthr)$$

For each quadrant, a statistic S_{ij} and an error rate p_{ij} is computed. $S_{ij} > 10$ and $p_{ij} < 0.15$ are the thresholds used on the BooleanNet statistics to identify BIRs. False discovery rate is computed by randomly shuffling each gene and computing the ratio of the number of BIR discovered in the randomized dataset and original dataset. The false discovery rate for our dataset was less than 0.001.

Boolean analysis on the large human colon tissue database (n = 1911; Global 2018 GPL570 Colon Purged), uses a threshold of sThr = 10 and pThr = 0.15. We increased sThr and relax the pThr to focus on reasonable number of gene candidates. Boolean Implication analysis looks for invariant relationship across all the different types of samples regardless of the conditions and treatment protocols. Therefore, it does not distinguish the sample types when discovering BIRs. We assume that there are fundamental invariant Boolean implication formula that are satisfied by every sample regardless of their type.

CANDiT (Cancer Associated Nodes for Differentiation Targeting). We developed a machine learning framework, *CANDiT*, designed to identify actionable nodes that drive differentiation within cancer networks to guide therapeutic intervention. *CANDiT* is agnostic to tissue of origin and begins with a single 'seed' gene that meets two essential criteria: (i) it must be a lineage-defining transcriptional determinant along the stemness-to-differentiation axis in the cancer's tissue of origin, and (ii) it must hold prognostic relevance in human tissue datasets, linking its expression to patient outcomes. Thus, the seed gene must bridge two levels of biological scale—cellular differentiation and clinical prognosis.

CANDiT constructs a BIN by identifying all statistically significant pairwise BIRs[8] within a curated expression compendium, the Human Colon Global Database, as previously described.[52] The resulting BIN is a directed graph in which nodes represent genes and edges represent one of six possible Boolean relationships (e.g., high ⇒ high, high ⇒ low, low ⇒ high, low ⇒ low, Equivalent, Opposite). While Equivalence relationships often form a scale-free network, other asymmetric BIRs do not.

Boolean analysis was performed on genes with sufficient dynamic range to permit clear binary classification (high/low) via the *StepMiner* algorithm. Based on over a decade of experience with Boolean implication methods, BIRs are most reliable in datasets with >200 samples.[140] Our dataset was prepared for Boolean analysis by filtering genes that had a reasonable dynamic

range of expression values. Genes with fewer than 5% of samples in either the high or low state were excluded to reduce noise and improve robustness. This filtering step ensures the resulting network highlights biologically meaningful and robust relationships.

Generation of Clustered Boolean Implication Network. Clustering was performed in the BIN to dramatically reduce the complexity of the network. A CBIN was created by clustering nodes in the original BIN by following the equivalent BIRs. One approach is to build connected components in an undirected graph of Boolean equivalences. However, because of noise, the connected components become internally inconsistent e.g., two genes opposite to each other become part of the same connected component. In addition, the size of clusters became unusually big with almost everything in one cluster. To avoid such a situation, we need to break the component by removing the weak links. To identify the weakest links, we first computed a minimum spanning tree for the graph and computed the Jaccard similarity coefficient for every edge in this tree. Ideally if two members are part of the same cluster, they should share as many connections as possible. A threshold of 0.7 is considered for the Jaccard similarity coefficient and if they share less than 70% of their total individual connections (Jaccard similarity coefficient less than 0.7) the edges are dropped from further analysis. Thus, many weak equivalences were dropped using the above algorithm leaving the clusters internally consistent. We removed all edges that have Jaccard similarity coefficient less than the selected threshold and built the connected components with the rest. The connected components were used to cluster the BIN that is converted to the nodes of the CBIN. The choice of the threshold on the Jaccard similarity coefficient play an important role in determining the size and the number of clusters as well as whether they are internally consistent. A new graph was built that connected the individual clusters to each other using Boolean relationships. The link between two clusters (A, B) was established by using the top representative node from A that was connected to most of the members of A and sampling 6 nodes from cluster B and identifying the overwhelming majority of BIRs between the nodes from each cluster.

Here, CBIN was created using our pooled dataset (human colon tissue database (n = 1911) to capture the differentiation events in colon tissue (Figure S1C). The edges between the clusters represented the Boolean relationships that are color-coded as follows: orange for low => high, dark blue for low => low, green for high => high, red for high => low, light blue for the equivalent and black for the opposite. A subnetwork is selected using low=>low (blue), high => low (red) and opposite (merged with high=>low as red) edges among the top 10 clusters (Figure S1D).

Charting Boolean paths. Boolean paths have been previously leveraged to predict the underlying time series events in biological processes such as B cell differentiation[32,33] and early differentiation events in CSC.[40,43,51,52] The core algorithm enabling this, *MiDReG* (Mining Developmentally Regulated Genes), uses two seed genes infer intermediate genes and transitional states within a developmental or differentiation process. *MiDReG* identifies these intermediates through a series of asymmetric BIRs.[10]

In this study, we apply the *MiDReg* framework to traverse the CBIN, identifying gene clusters whose start and endpoints may define biologically meaningful differentiation trajectories in colon tissue. The asymmetric BIRs offer a directional, causal logic that distinguishes this network from conventional co-expression or correlation-based gene networks.

A Boolean path refers to a directed sequence of BIR-linked nodes (genes) within the BIN. A simple Boolean path includes two nodes and a single directed edge, while a complex Boolean path involves multiple nodes and edges, representing multi-step transitions between regulatory states. These paths allow for a high-resolution mapping of potential developmental hierarchies or transitions embedded in steady-state transcriptomic data.

Ordering samples based on composite scores along Boolean paths. Each Boolean path comprises one or more clusters. To assign a composite score to each sample, we first computed a normalized average expression value for the genes within each cluster. Normalization was performed using a modified Z score centered around the *StepMiner* threshold (SThr), defined as: (formula = (expr - SThr)/3*stddev).

Next, a weighted linear combination of these cluster averages was used to generate a final composite score for each sample. Weights along the Boolean path were assigned to either monotonically increase or decrease—ensuring that the sample ordering remained consistent with the logical sequence dictated by the asymmetric BIRs. Clusters highly expressed in disease samples were assigned a weight of +1, while those predominant in healthy samples received a weight of −1. The directionality of the Boolean path—from healthy to disease states—guided this weighting scheme. Samples were then ordered based on their final composite score, enabling biologically meaningful stratification. The genes contained within each cluster of the Boolean network are detailed in Table S2.

Measurement of classification strength or prediction accuracy

To evaluate classification strength and prediction accuracy, Receiver Operating Characteristic (ROC) curves were generated for each gene. These curves assess the performance of a binary classifier, e.g., high vs. low *StepMiner* normalized gene expression levels, across varying discrimination thresholds. ROC curves plot the True Positive Rate against the False Positive Rate at multiple threshold levels. The AUC quantifies the classifier's ability to correctly distinguish between the various types of samples, in this instance, CRC or healthy and treated or not with drug. ROC-AUC values were computed using the Python Scikit-learn package.

Correlation coefficients analysis

Linear correlation analysis was performed between ΔCT values of *CCDC88A*, CDX2 and *PRKAB1* of all the CRC PDOs with their IC50 values. Regression analysis was performed using SciPy Stat package. Scatterplot with regression lines were generated using a Seaborn package (0.12.1).

Integrated differential expression analysis

Differential gene expression analysis was performed using RNAseq raw count data processed through DESeq2.[141] Genes with an absolute log2 fold change > 10 and an adjusted *p*-value <0.05 were considered DEGs. Pathway analysis of gene lists was carried out via the Reactome database and algorithm.[142] Reactome identifies signaling and metabolic molecules and organizes their relations into biological pathways and processes. A complete catalog of DEGs and Reactome pathway enrichments are provided in Tables S4 and S5, respectively. The 50-gene signature that emerged from the integration of all DEGs is provided in Table S6.

Survival analysis

StepMiner based survival analysis was performed by dividing the patients into two groups, based on high or low levels of expression of the 50-genes signature of therapeutic response [composite score expression values], as determined by implementing the *StepMiner*-derived threshold for each cohort (https://github.com/sinha7290/Prodiff). Log rank analysis and visualization were performed using GraphPad Prism version 9.1. Default settings of GraphPad Prism ignore events at time zero (t0) for the assessment of the impact of treatment modalities on outcome (https://www.graphpad.com/support/faq/events-deaths-at-time-zero-in-survival-analysis/). These default settings were manually modified to include all samples (without exception) in the cohort for which outcome was known. Survival analysis on KRAS^{WT} patient groups based on microsatellite stability status was performed using KM-plotter[92] based on *auto selected best percentile cutoff* of 50 genes.

Univariate and multivariate analyses

To assess which factor(s) may influence the IC50 of the PDOs, multivariate regression has been performed over all the clinical conditions. Here, the statsmodels module from python has been used to perform Ordinary least-squares (OLSs) regression analysis of each of the variables. The *p*-value for each term tests the null hypothesis that the coefficient is equal to zero (no effect).

We also performed univariate analysis on GSE39582 using the same OLS regression analysis to assess the impact of each variables individually, such as tumor stage, age, gender, tumor location, CIMP status, mutation status (*BRAF* and *TP53*) and gene expression [either *CDX2* alone or the composite score of the 50-gene signature of therapeutic response, both converted into a binary value, high vs. low, as determined by *StepMiner* threshold] on survival. The analysis was done on the entire cohort, as well as on *KRAS* WT and *KRAS* mutant patients as two independent sub-cohorts. Both the univariate and multivariate analysis data are represented based on the coefficient values of associated variables and their upper and lower bounds of 95% confidence interval as the error bars using Matplotlib. The significance of the variables was determined by t-test; * = $p < 0.05$, ** = $p < 0.01$, and *** = $p < 0.001$.

Unsupervised clustering and heatmap

Expression patterns of the genes that are differentially expressed in *CDX2*-low and -high groups, with or without PF treatment, are clustered without bias based on their z-normalized cpm expression values, in all the samples. The data are visualized using the seaborn clustermap package (v 0.12) in python.

Bulk RNAseq deconvolution

In silico deconvolution of bulk RNA-seq data from xenograft models was performed using the Granulator R package[143] to estimate monocyte abundance. Cell-type abundance estimates were normalized using the immune cell signature matrix developed by Monaco et al.[144]

Chemical synthesis of PRKAB1 agonist

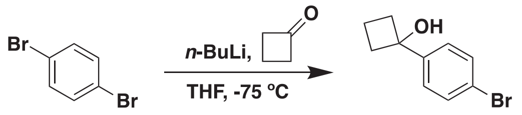

1-(4-Bromophenyl)cyclobutan-1-ol

An oven-dried 50 mL round-bottom flask was charged with 1,4-dibromobenzene (3.43 g, 14.6 mmol, 1.2 equiv) and anhydrous tetrahydrofuran (THF, 14 mL) and was cooled in a dry ice-acetone bath (−75°C). *n*-Butyllithium (*n*-BuLi, 2.5 M in hexane, 5.8 mL, 14.6 mmol, 1.2 equiv) was added dropwise. After stirring for 30 min, cyclobutanone (0.850 g, 12.1 mmol, 1 equiv) was added dropwise. The reaction mixture was stirred for 60 min at −75°C. The reaction was quenched by the addition of a saturated NH_4Cl (aq) solution (~7 mL). Water was added (~30 mL) and the solution mixture was extracted twice with Ethyl Acetate (EtOAc, ~100 mL x 2), the combined organics were washed with saturated brine (~50 mL), dried over $MgSO_4$, filtered, and concentrated *in vacuo*. The product was purified via SiO_2 column chromatography (using a gradient of 0%–10% to 15% EtOAc in hexanes as eluent) to give the title compound as a white solid (2.40 g, **87% yield**). 1H NMR (300 MHz, $CDCl_3$) δ (ppm) = 7.49–7.46 (d, 2H), 7.37–7.34 (d, 2H), 2.54–2.46 (m, 2H), 2.39–2.29 (m, 2H), 2.28 (s, 1H), 2.08–1.94 (m, 1H), 1.75–1.61 (m, 1H).

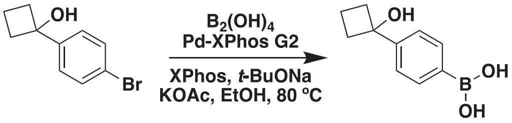

(4-(1-Hydroxycyclobutyl)phenyl)boronic acid

An oven-dried 100 mL round-bottom flask was charged with 1-(4-bromophenyl)cyclobutan-1-ol (2.00 g, 8.81 mmol, 1 equiv) and anhydrous ethanol (25 mL). Sodium tert-butoxide (8.5 mg, 0.088 mmol, 0.01 equiv), oven-dried potassium acetate (1.82 g, 18.5 mmol, 2.1 equiv), XPhos Pd G2 (chloro(2-dicyclohexylphosphino-2′,4′,6′-triisopropyl-1,1′-biphenyl)- [2-(2′-amino-1,1′-biphenyl)]palladium(II), 36 mg, 0.044 mmol, 0.005 equiv), and XPhos (2-dicyclohexylphosphino-2′,4′,6′-triisopropylbiphenyl, 42 mg, 0.088 mmol, 0.01 equiv) were added and the mixture was sparged with Argon. Tetrahydroxydiboron (1.58 g, 17.6 mmol, 2.0 equiv) was added and was stirred at 80°C for 3 h. The mixture was cooled to room temperature, and the solids were filtered through Celite to give a light-yellow solution. Water was added (~20 mL) and the solution mixture was extracted twice with EtOAc (~50 mL x 2), the combined organics were washed with saturated brine (~20 mL), dried over $MgSO_4$, filtered, and concentrated *in vacuo*. The product was purified via SiO_2 column chromatography (using a gradient of 50%–60% to 70% EtOAc in hexanes as eluent) to give the title compound as a white solid (0.900 g, **53% yield**). 1H NMR (300 MHz, DMSO-d6) δ (ppm) = 7.96 (s, 2H), 7.77–7.74 (d, 2H), 7.45–7.43 (d, 2H), 5.45 (s, 1H), 2.42–2.33 (m, 2H), 2.30–2.24 (m, 2H), 1.98–1.84 (m, 1H), 1.71–1.56 (m, 1H).

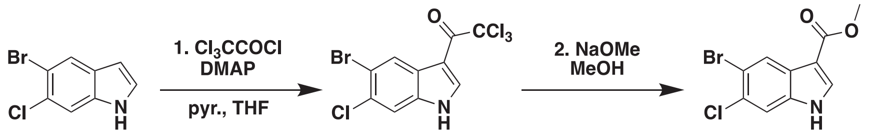

Methyl 5-bromo-6-chloro-1H-indole-3-carboxylate

An oven-dried 100 mL round-bottom flask was charged with 5-bromo-6-chloro-1H-indole (2.10 g, 9.11 mmol, 1 equiv), DMAP (Dimethylaminopyridine, 111 mg, 0.911 mmol, 0.1 equiv), pyridine (1.87 g, 23.7 mmol, 2.6 equiv), and anhydrous THF (14 mL) and then cooled to 0°C. Trichloroacetyl chloride (3.98 g, 21.9 mmol, 2.4 equiv) was then added dropwise, and then stirred for 3 days at room temperature. The mixture was then cooled to 0°C, and methanol (3.7 mL, 10 equiv) was added dropwise, then 25% sodium methoxide in methanol solution (4.2 mL, 2 equiv) was added dropwise. The mixture was stirred at 55°C for 2 h. Water was added (~20 mL) and the solution mixture was extracted twice with EtOAc (~50 mL x 2). The combined organics were washed with saturated brine (~20 mL), dried over $MgSO_4$, filtered, and concentrated *in vacuo*. The product was purified via SiO_2 column chromatography (using a gradient of 20%–30% EtOAc in hexanes as eluent) to give the title compound as an off-white solid (1.67 g, **63% yield**). 1H NMR (300 MHz, $CDCl_3$): δ (ppm) = 8.52 (br s, 1H), 8.45 (s, 1H), 7.92 (d, 1H), 7.56 (s, 1H), 3.93 (s, 3H).

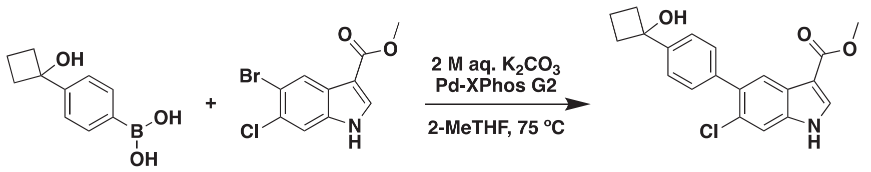

Methyl 6-chloro-5-(4-(1-hydroxycyclobutyl)phenyl)-1H-indole-3-carboxylate

An oven-dried 10 mL round-bottom flask was charged with methyl 5-bromo-6-chloro-1H-indole-3-carboxylate (280 mg, 0.970 mmol, 1 equiv), (4-(1-hydroxycyclobutyl)phenyl)boronic acid (205 mg, 1.07 mmol, 1.1 equiv), 2-MeTHF (2 mL), XPhos Pd G2 (chloro(2-dicyclohexyl-phosphino-2′,4′,6′-triisopropyl-1,1′-biphenyl)- [2-(2′-amino-1,1′-biphenyl)] palladium(II), 16 mg, 0.019 mmol, 0.02 equiv). Aqueous K_2CO_3 (362 mg, 2.62 mmol, 2.7 equiv in 1.1mL water, ~2.4 M) was added, and the mixture was sparged with Argon. The reaction mixture was heated to 75°C overnight (18 h). After cooling, the solvent was evaporated to dryness. Water was added (~10 mL) and the solution mixture was extracted three times with EtOAc (~10 mL x 3), the combined organics were washed with saturated brine (~10 mL), dried over $MgSO_4$, filtered, and concentrated *in vacuo*. The product was purified via SiO_2 column chromatography (using a gradient of 30%–40% to 50% EtOAc in hexanes as eluent) to give the title compound as a white solid (170 mg, **49% yield**). 1H NMR (300 MHz, DMSO-d6): δ (ppm) = 12.08 (br s, 1H), 8.17 (s, 1H), 7.93 (s, 1H), 7.66 (s, 1H), 7.58–7.55 (d, 2H), 7.41–7.39 (d, 2H), 5.55 (s, 1H), 3.79 (s, 3H), 2.48–2.41 (m, 2H), 2.35–2.26 (m, 2H), 2.00–1.91 (m, 1H), 1.76–1.64 (m, 1H).

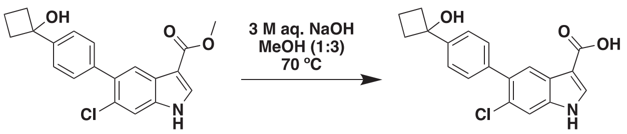

6-Chloro-5-(4-(1-hydroxycyclobutyl)phenyl)-1H-indole-3-carboxylic acid (PF06409577)

A 4-dram vial under Argon was charged with methyl 6-chloro-5-(4-(1-hydroxycyclobutyl)phenyl)-1H-indole-3-carboxylate (140 mg, 0.393 mmol, 1 equiv), 3M NaOH (aq) solution (0.787 mL, 2.36 mmol, 6 equiv), and methanol (MeOH, 2.36 mL). The reaction mixture was heated to 70°C overnight (18 h). After cooling, the solvent was evaporated to dryness. Water was added (~10 mL), the pH was adjusted to ~2 using 1M HCl (aq) solution, and the solution mixture was extracted three times with EtOAc (~10 mL x 3), the combined organics were washed with saturated brine (~10 mL), dried over $MgSO_4$, filtered, and concentrated *in vacuo*. The product was purified via SiO_2 column chromatography (using a gradient of 30%–60% Acetonitrile in Dichloromethane as eluent) to give the title compound as a white solid (54 mg, **40% yield**). 1H NMR (300 MHz, DMSO-d6): δ (ppm) = 11.94 (br s, 1H), 8.05 (s, 1H), 7.96 (s, 1H), 7.63 (s, 1H), 7.58–7.55 (d, 2H), 7.41–7.39 (d, 2H), 5.53 (s, 1H), 2.48–2.40 (m, 2H), 2.35–2.26 (m, 2H), 2.00–1.89 (m, 1H), 1.74–1.63 (m, 1H).

HPLC characterization was conducted using the Agilent 1260 Infinity II Quaternary Pump System with a 150 mm × 3 mm, 2.7 mm particle size C18 column (693975-302T). Solvent conditions (Solvent A: H2O (0.1% TFA), Solvent B: Acetonitrile): 0–2 min isocratic 5% B, 2–20 min gradient to 95% B, 20–22 min isocratic 95% B, at a flow rate of 1 mL/min. The chromatogram was monitored at 254 nm wavelength and the compound had a retention time of 11.956 min. The purity of this final product was 96% by HPLC.

Experimental methods

MTT (3-[4,5-dimethylthiazol-2-yl]-2,5 diphenyl tetrazolium bromide) assays on colorectal cancer cell lines

Cell survival was measured using the MTT reagent and cells cultured in 96-well plates. HCT116 and Sw480 cells were cultured and treated with different concentrations of PF compound (0, 1, 5, 10, 20 μM) using DMSO as a negative control for 16–48 h in various assays. Then the cell lines were incubated with MTT for 4 h at 37°C. After incubation, culture media was removed, and replaced with phosphate buffered saline (PBS), and 150 μL of DMSO was added in order to solubilize the MTT formazan crystals. Optical density was determined at 590 nm using a TECAN plate reader. At least three independent experiments were performed.

Cell cycle and apoptosis analyses

Cell cycle distribution and apoptosis were assessed using the Guava Cell Cycle Reagent (Millipore Sigma) and the Annexin V/Propidium Iodide (PI) staining kit (Thermo Fisher Scientific), respectively, following the manufacturers' protocols. Samples were analyzed on a BD LSR II flow cytometer, and data were processed using FlowJo software (FlowJo LLC, Ashland, OR).

Cell toxicity assays

Confluent flasks of HCT116 and Caco2 cells were rinsed with PBS and detached using 1X trypsin-EDTA in PBS. Cells were resuspended in 1 mL of their respective medias (HCT116: McCoy's 5A modified medium with 10% FBS and 1% Pen/Strep; Caco2: High glucose DMEM with 10% FBS, 1% NEAA, 1% P/S). Prior to seeding, 50 μL of media was added to each well of an Agilent xCELLigence 96-well impedance plate, and baseline cell index readings (2 sweeps) were recorded using the Agilent eSight live-cell analysis system. Cells were then counted and seeded at 10,000 cells/well in 50 μL of media. Cell index measurements were collected at 15-min intervals for 24 h to monitor attachment and proliferation.

After 24 h, the assay was paused to allow treatment with compounds or vehicle. Each well received 100 μL of treatment media containing 2X concentrations of the PF compound or DMSO (vehicle), along with 2X Annexin V. The plate was returned to the instrument and continuous monitoring resumed. Cell index measurements and live-cell images (4/well) were collected throughout the remainder of the 126-h assay. Cell index values were normalized to the final pre-treatment time point. Dose-response curves and Annexin-based toxicity were assessed at the 126-h endpoint.

Patient-derived organoid toxicity assay

PDOs were cultured for 72 h prior to treatment. Organoids were then exposed to complete medium containing either PF compound (5 μM or 20 μM), DMSO (vehicle), or 200 nM H2O2 (positive control), with 500 nM eTox Red added to detect cell death. After 72 h of treatment, Hoechst 33342 (5 μg/mL) was added to label nuclei, followed by a PBS wash and replacement with phenol red- and serum-free DMEM. Organoids were imaged using a BioTek Cytation 10 system. eTox Red-positive cells (defined by red fluorescence within nuclei) were quantified using BioTek Gen5 software, and the percentage of positive cells was calculated per condition. Data were analyzed and visualized using GraphPad Prism.

Immunohistochemistry

Tumor sections of 4 μm thickness were cut and placed on glass slides coated with poly-L-lysine, followed by deparaffinization and hydration. Heat-induced epitope retrieval was performed using sodium citrate buffer (pH 6) or Tris-EDTA (pH9) in a pressure cooker. Tumor sections were incubated with 3% H2O2 for 5-15 min to block endogenous peroxidase activity, followed by incubation with primary antibody overnight in a humidified chamber at 4°C. Antibodies used for immunostaining were anti-CDX2 (1:300, rabbit host, cat no. ab76541), AMPK β1 (1:200, rabbit host, cat no. 10308-1-AP), anti-pSer^{245}GIV (1:50, anti-rabbit), anti-YAP (clone 63.7; Santa Cruz: sc-101199), anti-phospho(p)YAP Ser^{87} (Cell Signaling Technology Cat#13008) and anti-β-Catenin (1:300, mouse host, SC-7963). Antigen retrieval protocols were optimized based on manufacturer's protocols. Immunostaining was visualized with a labeled streptavidin–biotin using 3,3′-diaminobenzidine as a chromogen and counterstained with hematoxylin. Immunohistochemistry (IHC) images were randomly sampled at different regions of interest (ROIs). The ROIs were analyzed using IHC Profiler.[145] IHC Profiler uses a spectral deconvolution method of DAB/hematoxylin color spectra using optimized optical density vectors of the color deconvolution plugin to properly segregate the DAB color spectra. The histogram of the DAB intensity was divided into 4 zones: high

positive (0–60), positive (61–120), low positive (121–180), and negative (181–235). High positive, positive, and low positive percentages were combined to compute the final percentage positive for each ROI. The range of values for the percent positive was compared among the different experimental groups.

Immunofluorescence

Tumor sections of 4 μm thickness were cut and placed on glass slides coated with poly-L-lysine, followed by deparaffinization and hydration. Heat-induced epitope retrieval was performed using Tris-EDTA (pH9) in a pressure cooker. Samples were blocked for 1 h using an in-house blocking buffer (1% BSA and 0.01% Tween 20 in TBS). Primary antibodies were diluted in blocking buffer and allowed to incubate overnight at 4°C; antibodies used were E Cadherin (H-108) cat no. SC7870, 1:50, and Anti-Beta Catenin cat no. SC7963, 1:50. Secondary antibodies, Goat anti-mouse IgG H&L secondary antibody, Alexa Fluor 488 and Goat anti-rabbit IgG H&L secondary antibody, Alexa Fluor 594, were diluted in blocking buffer and allowed to incubate for 2 h in the dark. ProLong Glass was used as a mounting medium. #1 Thick Coverslips were applied to slides and sealed. Samples were stored at 4°C until imaged.

Quantification of nuclear area

FFPE xenograft sections were stained with hematoxylin and eosin (H&E) using standard protocols. Whole slide images were acquired using a high-resolution slide scanner. Representative fields from vehicle- and PF-treated xenografts were exported as individual JPG files for image analysis. To quantify nuclear morphology, images were processed using ImageJ with the StarDist plugin (v0.3.0), trained on an H&E-compatible model for nuclear detection. Each image was converted to 8-bit grayscale, and contrast was adjusted as needed. StarDist-generated nuclear segmentation masks were used to define ROI, and nuclear area was quantified for all detected nuclei in each field. Data visualization and statistical analysis were performed using GraphPad Prism.

Estimation of IC_{50} by MTT assays on patient-derived organoids

To attain IC50 values for CRC PDM lines and healthy colon organoid lines in response to PF-06409577, thiazolyl blue tetrazolium bromide (MTT) reduction assays were performed, based on a previous protocol[146] with modifications. Prior to experiment, $5x10^3$-$1x10^4$ dissociated single cells were seeded in Matrigel in 96-well tissue culture plates. After 3 days in culture, organoid images were captured on EVOS XL Core Imaging System. Organoids were administered complete medium or complete medium treated on alternate days with 1 μM, 5 μM, 10 μM, 20 μM, or 40 μM PF-06409577 (final concentration), while ensuring that only 50% of the media was replenished during subsequent repeated treatments. After 7–9 days of treatment, when apoptosis was most apparent, organoids were imaged and MTT reagent was added to each well for a final MTT concentration of 588 μg/mL. Organoids were incubated in MTT for 4 h at 37°C, 5% CO_2, after which images were acquired to document the extent of formazan formation. To prepare the plate for quantification, Matrigel was dissolved in 2% SDS, followed by formazan dissolution in dimethyl sulfoxide. The optical density of each well was measured at 562 nm using Tecan Spark Multimode Microplate Reader.[146] The measured optical densities at each concentration were used to calculate an IC50 for each CRC PDO and healthy colon line.

Immunoblotting

To verify the expression of markers of stemness and differentiation, equal aliquots of whole-cell lysates (prepared using RIPA buffer) were loaded on an 8% SDS PAGE gel, and immunoblotting was carried out for various targets as described previously.[147] Immunoblots were analyzed using a Gel Doc system (BioRad, Hercules, CA).

RNA extraction for transcriptomic analysis

To collect RNA from CRC PDOs, dissociated single cells were seeded in Matrigel in 24-well tissue culture plates. After 3 days in culture, organoids were administered complete medium or complete medium treated with 10 μM PF-06409577. Prior to treatment with PF-06409577 and after 2 days of treatment, whole organoids were collected in Dulbecco's phosphate-buffered saline with 0.5 mM ethylenediaminetetraacetic acid (PBS-EDTA). Organoids were subsequently incubated in cell recovery solution, pelleted, and stored at −80°C. Total RNA was extracted using the Quick-RNA Microprep Kit and concentration was measured using Nanodrop One^C Spectrophotometer. For qPCR, first-strand cDNA synthesis was performed using qScript cDNA SuperMix. For RNA sequencing, isolated RNA has been processed for RNA sequencing in the Illumina NovaSeq 6000 platform. Fastq sequence files have been mapped using the human GRCh38 genome.

QUANTIFICATION AND STATISTICAL ANALYSIS

Optimal gene expression cut-off values were determined using the *StepMiner* algorithm within each individual dataset.[81] Gene signatures were used to classify sample categories, and multi-class classification performance was evaluated using ROC-AUC (ROC AUC) values. Statistical comparisons were performed using Welch's two-sample t-test (unpaired, unequal variance and sample size) via the scipy.stats.ttest_ind function in Python (version 0.19.0; equal_var = False). Multiple hypothesis correction was applied using the Benjamini-Hochberg method (fdr_bh) via statsmodels.stats.multitest.multipletests. Differential expression analysis was conducted using DESeq2 in R (version 1.16.1). Kaplan–Meier survival analysis and log rank tests for statistical significance were performed using GraphPad Prism 9.1. Violin and bubble plots were generated using Seaborn (Python, version 0.12). BIRs between genes were determined using the BooleanNet algorithm (criteria: statistic >3 and error rate <0.1).

Definition of n: For PDO and cell line experiments, n represents the number of independent biological replicates (organoid lines, independent wells, or passages). For xenograft experiments, n represents the number of individual mice per group. For transcriptomic datasets, n corresponds to the number of patients included. Exact values of n are provided in figure legends, Results text, and Supplemental Tables.

Dispersion and precision measures: Data are reported as mean ± standard deviation (SD) or mean ± standard error of the mean (SEM), as indicated in figure legends. Confidence intervals (95% CI) are reported for odds ratios, hazard ratios, and regression coefficients.

Power analysis: Effect sizes (mean difference/SD) were estimated at 2.5 in PDOs and 1.0 in animal experiments for CDX2 induction with the PRKAB1 agonist. A minimum of 4 PDOs and 17 animals per group was determined to provide adequate power ($\alpha = 0.05$, power = 0.80).

All statistical details of experiments, including exact *p*-values, test types, and sample sizes, are reported in the figure legends, Results, and Supplemental Tables.

Cell Reports Medicine, Volume *6*

Supplemental information

CANDiT: A machine learning framework

for differentiation therapy in colorectal cancer

Saptarshi Sinha, Joshua Alcantara, Kevin Perry, Vanessa Castillo, Annelies K. Ondersma, Satarupa Banerjee, Ella McLaren, Celia R. Espinoza, Sahar Taheri, Eleadah Vidales, Courtney Tindle, Adel Adel, Siamak Amirfakhri, Joseph R. Sawires, Jerry Yang, Michael Bouvet, and Pradipta Ghosh

SUPPLEMENTARY FIGURES AND LEGENDS

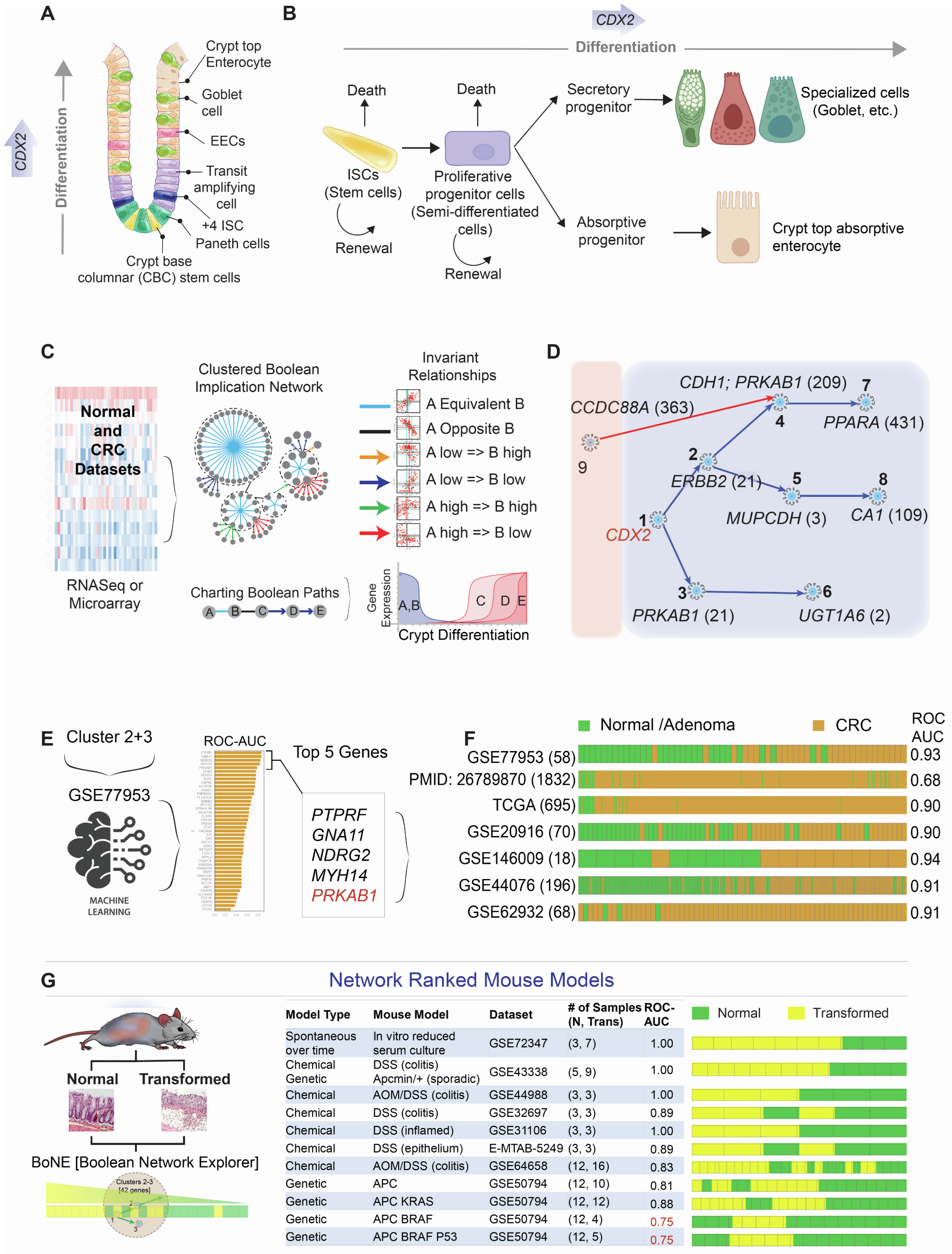

Figure S1 [Related to Figure 1].

Boolean Network-Based Identification of CDX2-Linked Differentiation Targets. A. Schematic of epithelial differentiation in the colonic crypt, where stem cells reside at the crypt base, and fully differentiated goblet cells and enterocytes localize toward the top. **B**.

Illustration of the continuum of crypt differentiation, highlighting various progenitor cell states along the axis from stemness to terminal differentiation. **C**. Overview of the Clustered Boolean Implication Network (BoNE) workflow. Diverse transcriptomic datasets are integrated to generate clusters of co-expressed genes, which are interconnected through one of six invariant Boolean relationships. **D**. Boolean network centered on *CDX2* as a seed gene, generated using the computational platform, Boolean Network Explorer (BoNE[30]), using a pooled microarray dataset (1662 CRCs, 68 adenomas, 170 normal colon tissues). Directed edges indicate Boolean relationships. **E-F**. Machine learning-driven target prioritization. Boolean clusters directly connected to *CDX2* (clusters #2 and #3) were evaluated by linear regression on test datasets. Top five genes (E) from each cluster were selected based on their ability to distinguish CRC from normal tissue (F: GSE dataset identifiers, bar plots, and ROC-AUC values shown). **G**. ROC-AUC analysis showing the ability of Boolean clusters #2 and #3 (which include *PRKAB1* and *CDX2*) to distinguish normal from transformed (and/or inflamed colitic) states in murine colitis models based on gene expression patterns.

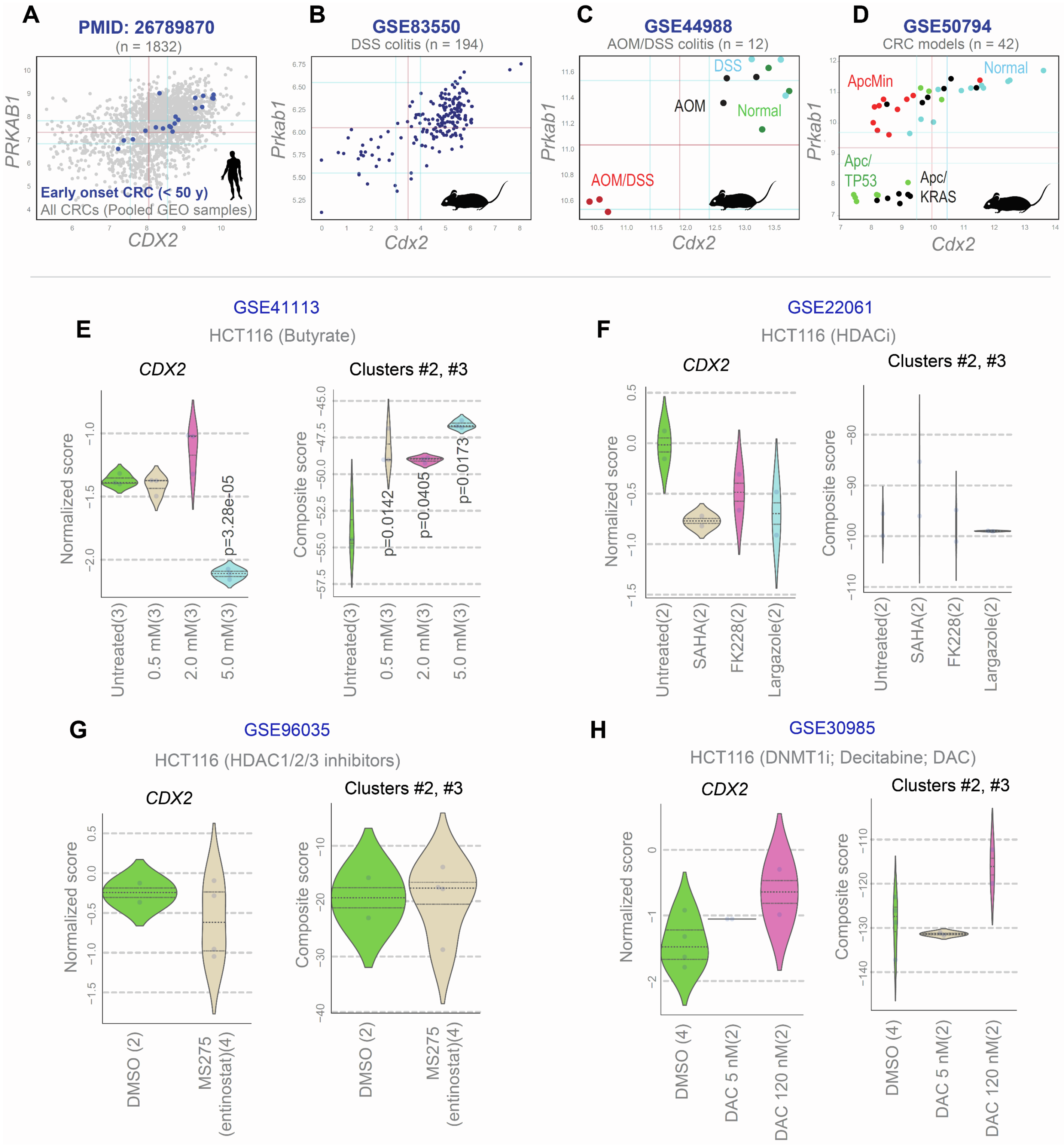

Figure S2 [Related to Figure 1].

PRKAB1 Exhibits a Conserved Universal Relationship with and Predicted to Restore CDX2 when Prior CDX2-Restorative Therapeutics have Failed. A-D. Scatter plots demonstrating a universal equivalent Boolean relationship between *PRKAB1* and *CDX2* across multiple pooled transcriptomic datasets from human and murine models of colorectal cancer (CRC) and colitis. **E-H**. Violin plots show the expression pattern of *CDX2* (left) and network-derived gene clusters #2 and #3 (right) as an effect of various treatments in HCT116 cell lines. *Statistics*: p-values were calculated by Welch's t-test (compared to untreated or control sample#1 in each plot). Only significant p-values are shown.

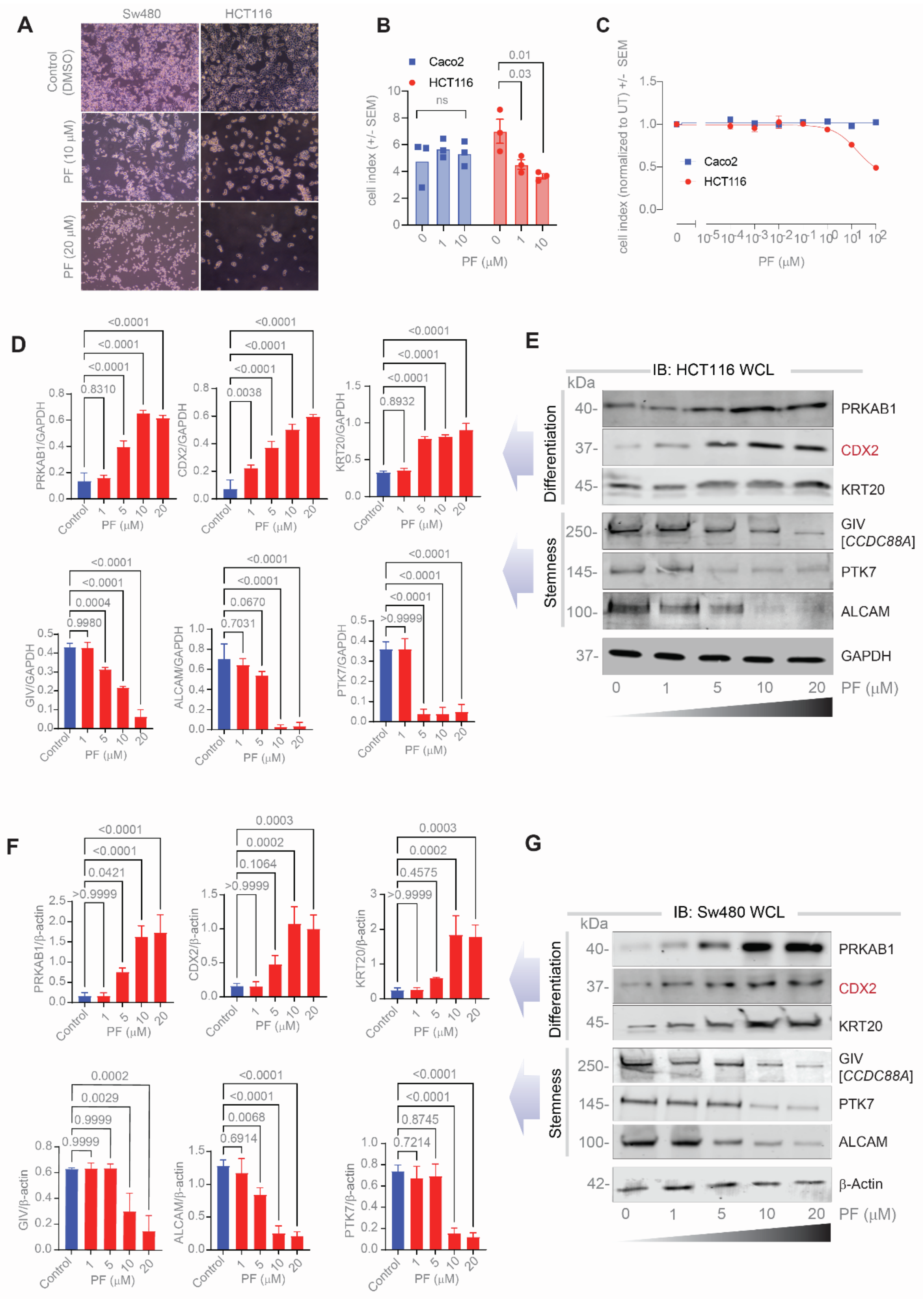

Figure S3 [Related to Figure 2].

PRKAB1 Agonist (PF) Induces Differentiation in CRC Cell Lines. A. Bright-field images (10×) of CRC cell lines treated with 10 µM and 20 µM PF compound for 16 hours. **B-C**. Bar plot (B) showing cell index, measured as impedance, for CDX2-high Caco2 cells and CDX2-low HCT116 cells after 128 h of incubation with varying concentrations of PF compound. Line plot showing normalized cell index (relative to untreated control) during 128 h of incubation with varying concentrations of PF compound. **D-G**. Immunoblots and quantification of HCT116 WCLs (D-E) and Sw480 WCLs (F-G) treated with increasing PF doses for 16 hours prior to lysis (when there is <1-2% cell death). *Statistics*: p-values were calculated using one-way ANOVA. Error bars represent S.E.M. from three independent biological replicates.

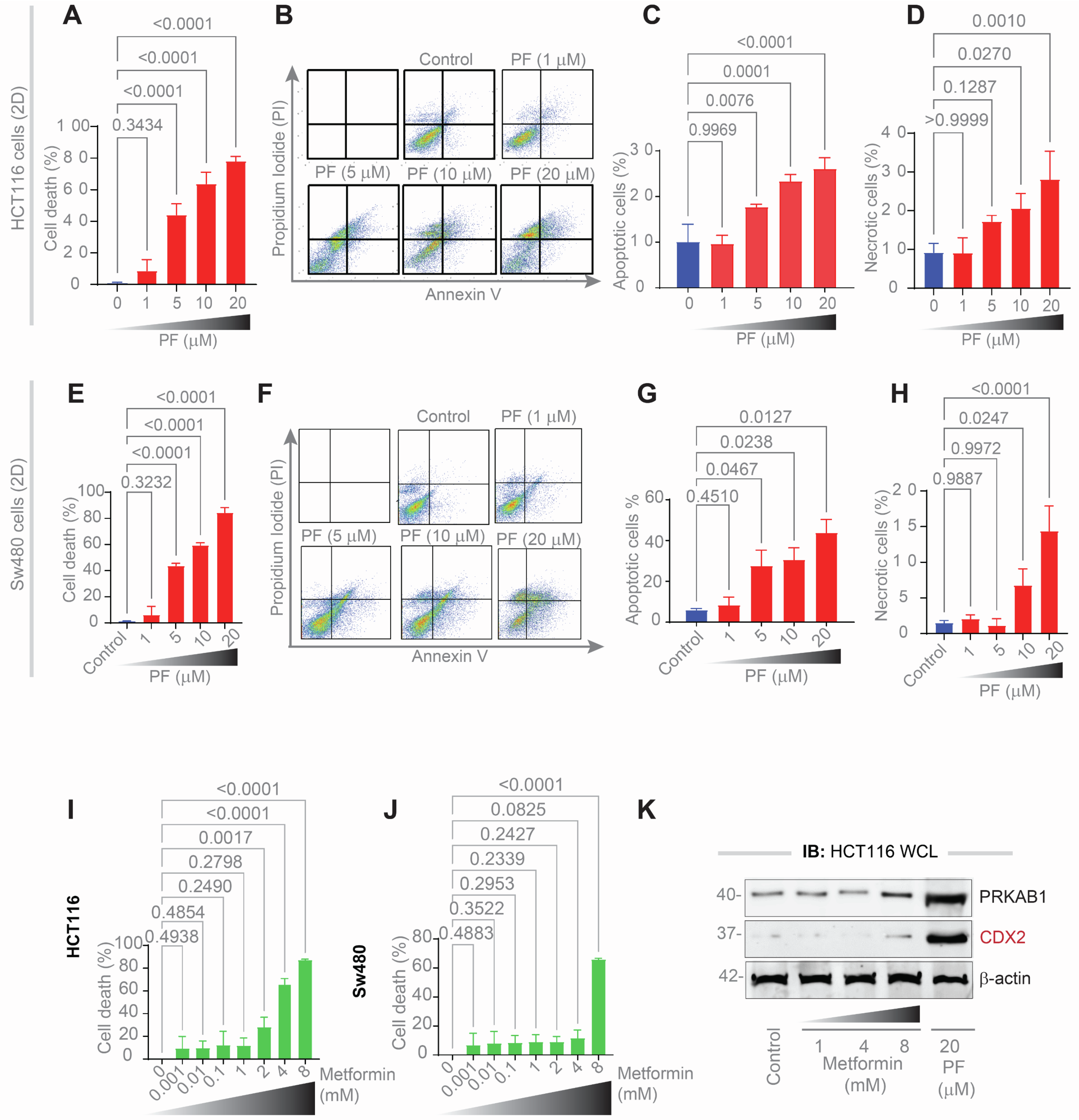

Figure S4 [Related to Figure 2].

β1-AMPK Specific Agonist (PRKAB1-agonist, PF), but Not the Indirect AMPK Activator Metformin, Induces Dose-Dependent Cell Death. A-H. Monolayers of HCT116 (A-D) and Sw480 (E-H) cells were treated with increasing concentrations of the PRKAB1 agonist PF and assessed for apoptosis using Annexin V staining followed by flow cytometry. Representative scatter plots from flow cytometry are shown in B and F. **I-J**. Monolayers of HCT116 (I) and Sw480 (J) cells were treated with various concentrations of the indirect AMPK activator Metformin, and cell viability was measured via MTT assay. **K**. Immunoblots of equal aliquots of whole cell lysates (WCL) of HCT116 cells treated with increasing doses of Metformin and 20 µM PF for 16 h prior to lysis (when there is virtually no cell death). β-actin was used as loading control. *Statistics*: p-values were calculated by one-way ANOVA. Error bars indicate S.E.M of 3 independent biological replicates.

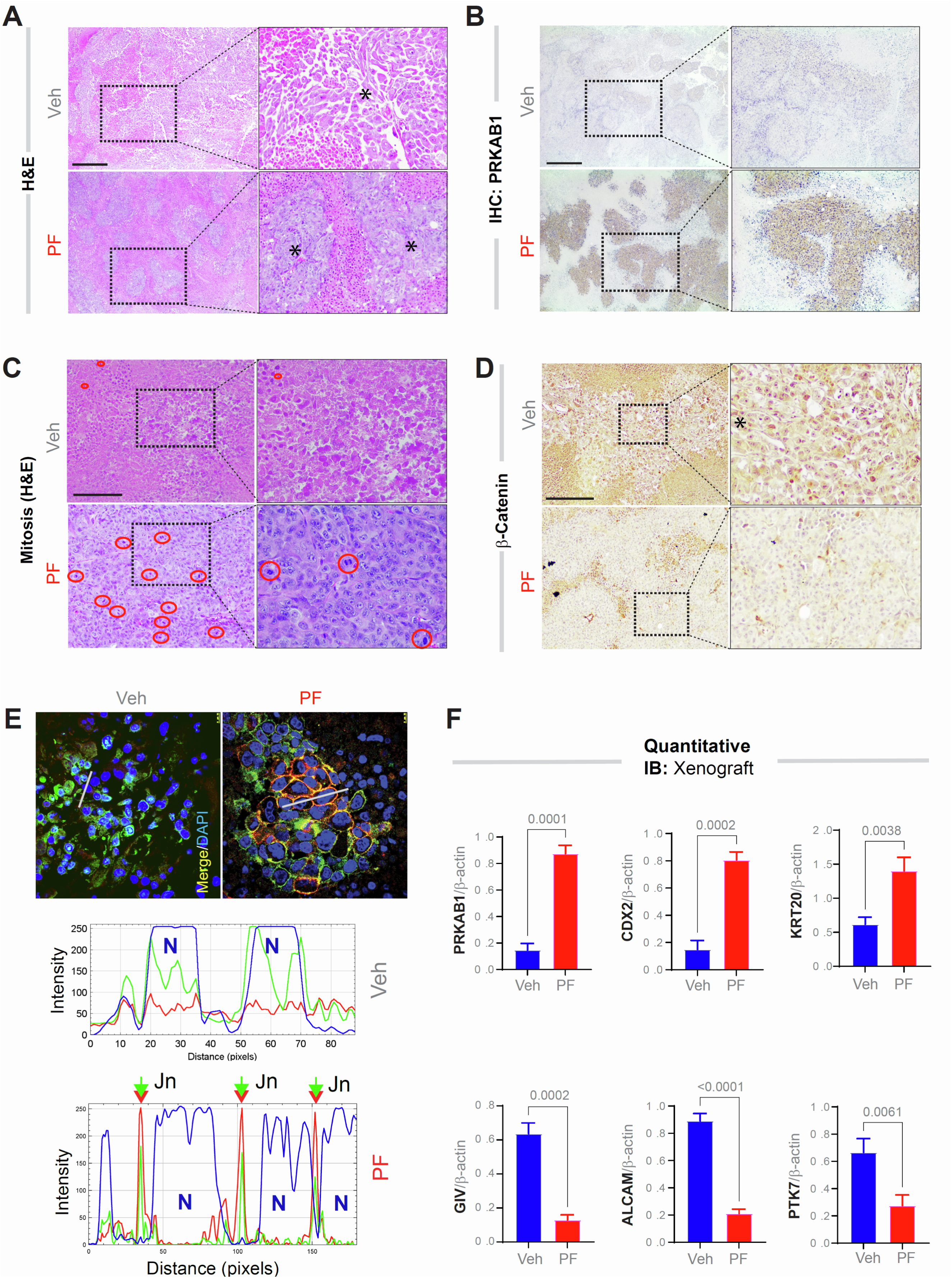

Figure S5 [Related to Figure 2].

PRKAB1-agonist (PF) Promotes Differentiation in Xenotransplants. A. Representative H&E-stained sections of FFPE tumor xenografts. Vehicle (Veh) treated tumors exhibit poorly differentiated, rhomboid-shaped cells arranged in sheet-like patterns with

minimal extracellular matrix. In contrast, PF-treated tumors display focal regions of glandular architecture with prominent cell-cell contacts. Asterisks (*) denote viable tumor cell islands; red circles in panel C highlight mitotic figures. **B-D**. Representative fields of H&E and IHC-stained FFPE tumor sections. Scale Bars = 100 µm. **E**. Representative merged images (top) of xenograft sections co-stained for β-catenin (green), E-cadherin (red), and DAPI (blue). RGB intensity plots (bottom, generated in ImageJ) demonstrate co-localization of β-catenin and E-cadherin at cell-cell junctions, indicating membrane-localized β-catenin specifically in PF-treated tumors. Scale bar = 10 µm. See individual channels in **Figure 2N**. **F**. Quantification of differentiation and stemness markers from immunoblots of xenograft lysates in **Figure 2O**. *Statistics*: p-values were calculated by paired t-test. Error bars indicate S.E.M of 3 independent biological replicates.

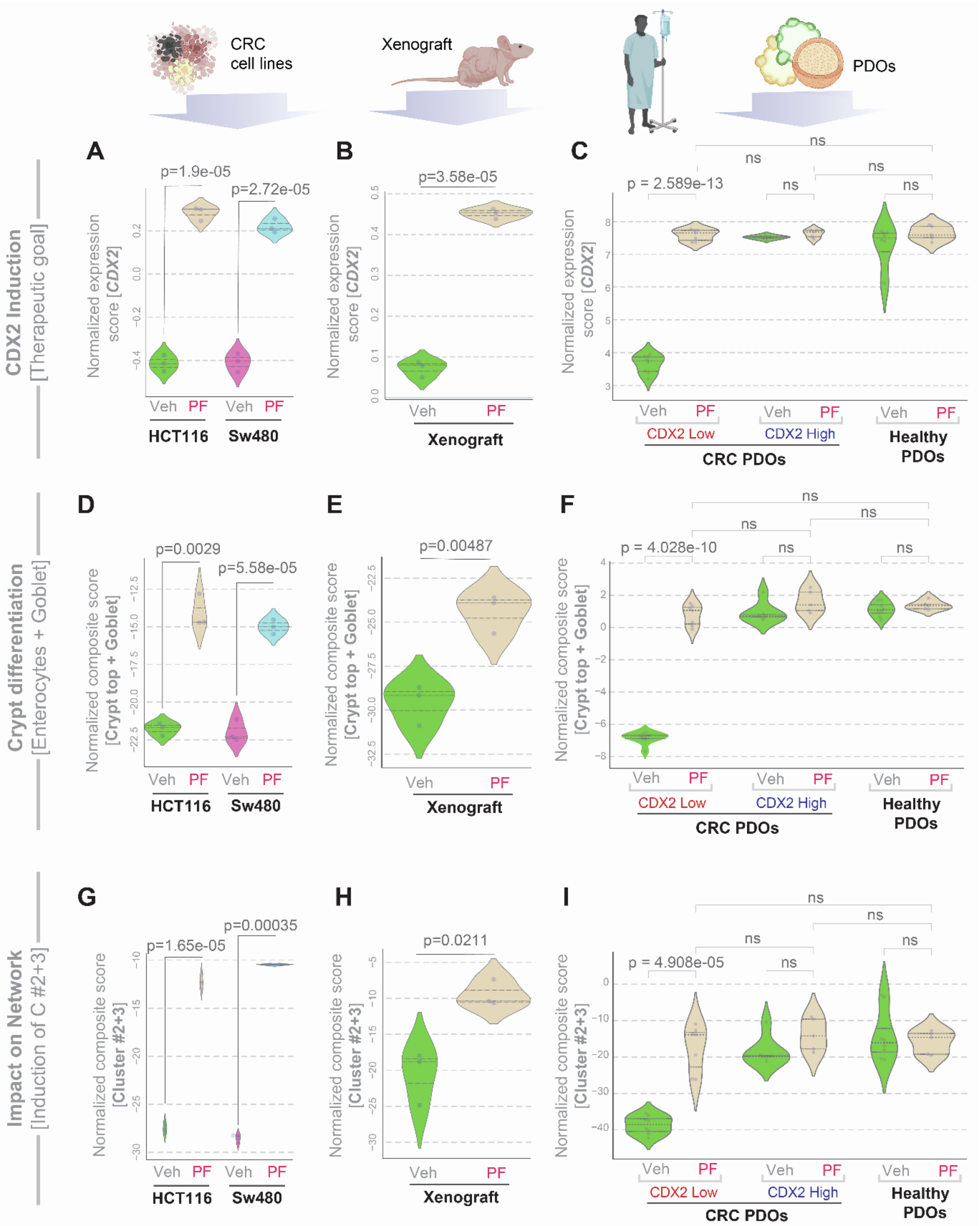

Figure S6 [Related to Figure 4].

PF treatment induces *CDX2*, markers of crypt differentiation, and genes in cluster #2 + #3 in the CRC network in CRC cell lines, xenografts, and PDOs. Violin plots display the normalized expression of *CDX2* (A-C), markers of absorptive enterocytes and Goblet cells indicative of crypt differentiation (D-F), and composite score of genes in clusters #2 and #3 from proposed CRC network (G-I) in CRC cell lines (A, D, G), tumor xenografts (B, E, H) and PDOs (C, F, I). See **Supplemental Table 2** for a compete catalogue of genes in clusters #2 and #3 and the markers of crypt differentiation. *Statistics*: p-values were calculated by Welch's t-test.

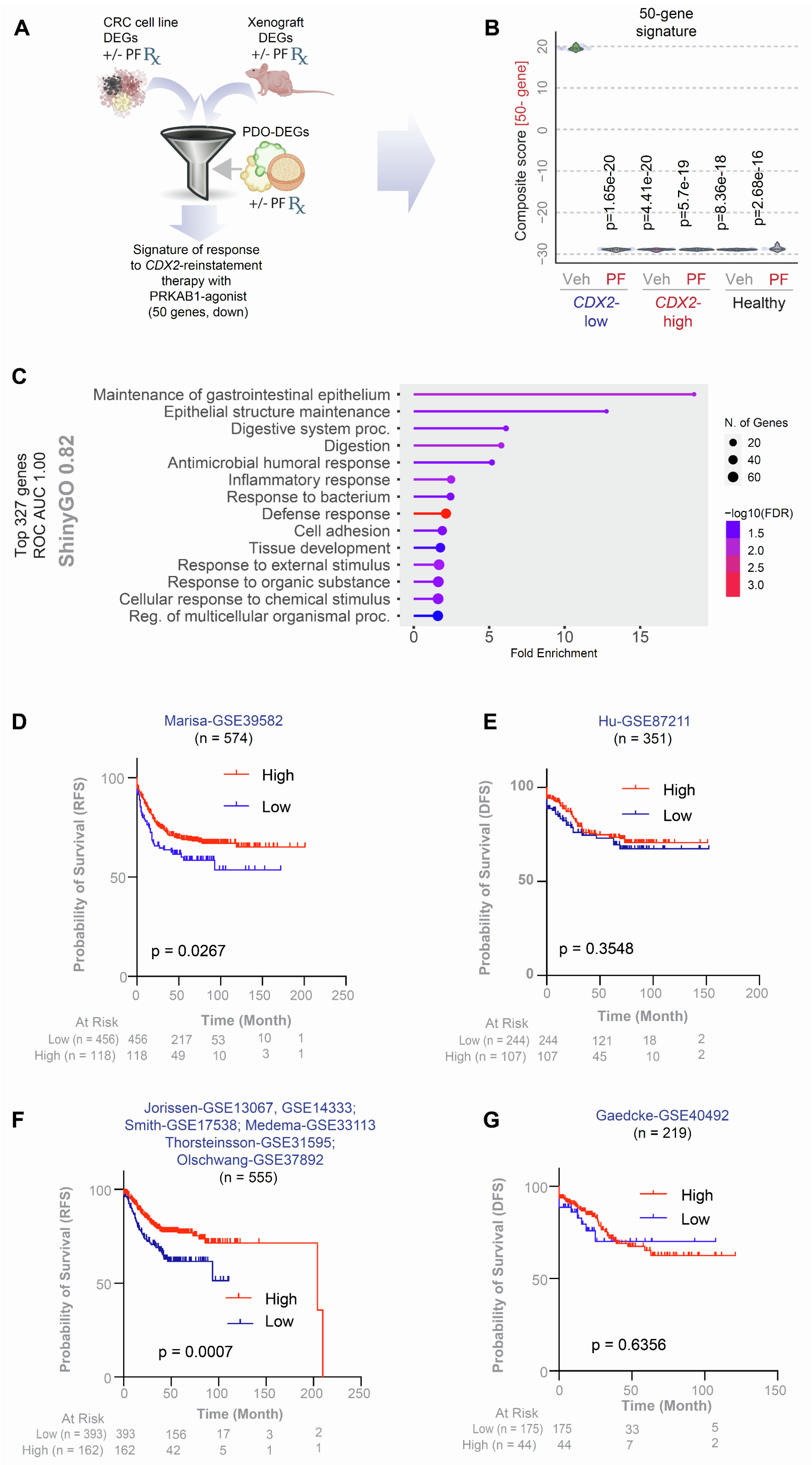

Figure S7 [Related to Figure 5].
A gene signature of therapeutic response predicts better survival in patients.

A. Computational workflow used in the derivation of a 50-gene signature of therapeutic response using an integrated DEA approach. Differentially expressed genes (DEGs) from CRC cell lines and xenografts (see **Supplemental Table 4-5**) were further refined based on their ability to accurately classify PDO samples with/without PF treatment (see **Supplemental Table 6** for gene list rank-ordered by ROC-AUC score). **B.** Violin plots show composite scores of the 50-gene signature in PDOs. p-values are based on Welch's t-test compared to vehicle (Veh)-treated *CDX2*-low PDOs. **C**. Lollipop plots depict pathway enrichment for the top 327 differentially expressed genes (DEGs) induced upon PF treatment in CRC cell lines and xenografts, which also achieved 100% classification accuracy (i.e., ROC AUC 1.00; **Supplemental Table 6**) in PDO samples. **D-G.** Kaplan-Meier plots showing probability of survival (DFS/RFS) over time in various cohort, segregated by high vs low expression scores of *CDX2*, computed using the *StepMiner* algorithm[82] within each cohort. p-value was determined by Log Rank test. See **Supplemental Table 7** for the corresponding O.R.

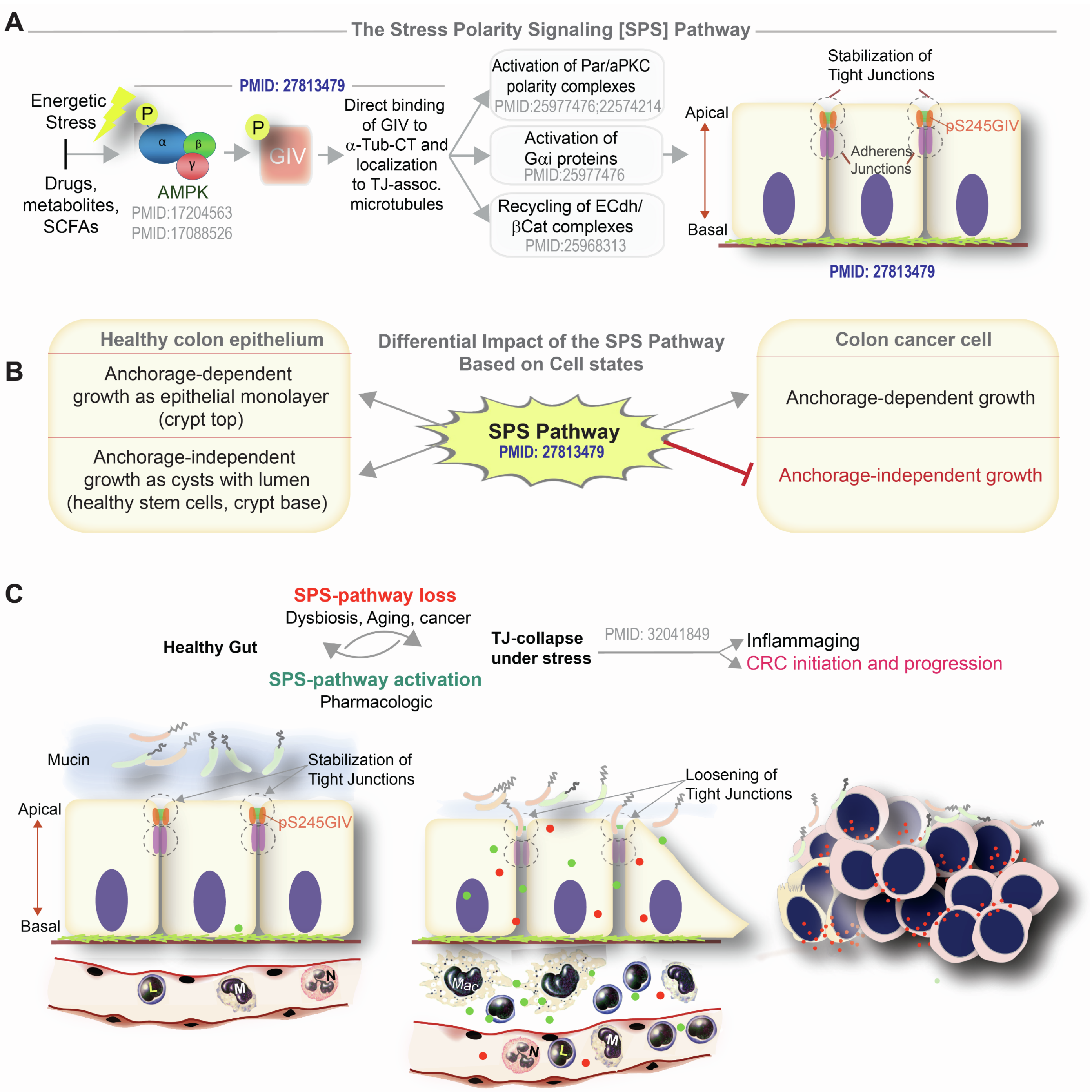

Figure S8 [Related to Figure 6].

The Stress Polarity Signaling (SPS) Pathway Maintains Epithelial Polarity in Health and Restrains Malignant Transformation.

A. Schematic overview of the SPS pathway[78-80]. Activation of a junction-localized β1-specific pool of trimeric αβγ-AMPK leads to phosphorylation of GIV at a Serine in position 245 (pSer[245]), which in turn activates Par/aPKC complexes and Gαi proteins. This cascade promotes the recycling of E-cadherin/β-catenin complexes, thereby stabilizing tight junctions and reinforcing apical polarity in epithelial cells. Corresponding PMID#s are listed.

B. Diagram illustrating previously published[6] work on the cell state-dependent cell fates induced by the SPS pathway. In healthy epithelial cells, SPS pathway activation permits anchorage-independent cystic growth with preserved lumens and anchorage-dependent growth as monolayers with enhanced barrier function. By contrast, activation of the SPS pathway in SPS-deficient transformed CRC cells is permissive only to anchorage-dependent growth as polarized monolayer but virtually abolishes growth in anchorage independent mode.

C. Schematic summarizes published work on the status of the SPS pathway in health and disease. In healthy intestinal epithelium, physiological or drug-induced SPS activation enhances apico-basal polarity and tight junction integrity via GIV phosphorylation. In pathological states (e.g., dysbiosis, aging, or cancer), SPS pathway is suppressed or lost, leading to tight junction breakdown, compromised barrier function, and increased susceptibility to inflammation and CRC progression

SUPPLEMENTARY TABLES

Supplemental Table 1 [Related to Figure 1 and S1]: A catalogue of all datasets used in model training and validation.

Model Training					
**Dataset Identifier**	**Species**	**No. of samples used**	**Normal colon**	**Cancer colon**	**Adenomas**
GSE2109	Human	393	0	393	0
GSE14333	Human	225	0	225	0
GSE26682	Human	175	0	175	0
GSE13294	Human	155	0	155	0
GSE37892	Human	129	0	129	0
GSE18105	Human	110	16	94	0
GSE20916	Human	145	44	91	10
GSE13067	Human	73	0	73	0
GSE9348	Human	82	12	70	0
GSE17538	Human	64	0	64	0
GSE26906	Human	57	0	57	0
GSE18088	Human	53	0	53	0
GSE31595	Human	37	0	37	0
GSE4183	Human	38	8	15	15
GSE4107	Human	20	10	10	0
GSE10714	Human	15	3	7	5
GSE15960	Human	18	6	6	6
GSE13471	Human	8	4	4	0
GSE10961	Human	4	0	4	0
GSE8671	Human	64	32	0	32
GSE9254	Human	18	18	0	0
GSE11831	Human	17	17	0	0
GSE31255	Human	11	0	0	11
**Model Validation**					
**Dataset Identifier**	**Species**	**No. of samples used**	**Normal colon**	**Cancer colon**	**Adenomas**
GSE77953	Human	58	30	28	-
PMID:26789870	Human	1832	170	1662	-
TCGA	Human	695	51	644	-
GSE20916	Human	70	34	36	-
GSE146009	Human	18	9	9	-
GSE44076	Human	196	98	98	-
GSE62932	Human	68	4	64	-
GSE83550	Mouse	194	-	-	-

GSE44988	Mouse	12	-	-	-
GSE50794	Mouse	42	-	-	-
GSE72347	Mouse	10	-	-	-
GSE43338	Mouse	14	-	-	-
GSE32697	Mouse	6	-	-	-
GSE31106	Mouse	6	-	-	-
E-MTAB-5249	Mouse	6	-	-	-
GSE64658	Mouse	28	-	-	-

Supplemental Table 3 [Related to Figure 3]: A catalogue of all covariates used in the multivariate analysis of the patient-derived organoids.

Catalog number	Age	Gender	Race	Primary Site	Acquisition Site	Clinical Stage Group	Histological Grade	APC	BRAF	KRAS	TP53	PTEN	PIK3CA	Number of mutated genes	Neoadjuvant therapy	IC50
PDM-255	73	Female	White	Colon	Cecum	Stage IVC	GB	no	no	no	no	no	no	218	yes	0.84
PDM-356	58	Male	Unknown	Colon	Sigmoid colon	ungrouped	G2	yes	no	no	yes	no	no	151	no	0.95
PDM-8	75	Male	Asian	Colon	Sigmoid colon	Stage IIA	G2	yes	no	no	yes	no	no	343	no	2
PDM-191	56	Male	White	Rectum	Sigmoid Colon	Stage IIIC	GX	no	yes	no	yes	no	no	219	yes	3.61
PDM-264	67	Female	White	Rectosigmoid junction	Rectosigmoid junction	Stage IIIB	G2	yes	no	no	yes	no	no	203	no	4.4
PDM-5	60	Female	White	Colon	Sigmoid colon	Stage IIIB	G2	yes	no	no	yes	no	no	131	no	4.5
PDM-275	73	Female	White	Colon	Lymph node(s)	ungrouped	ungrouped	no	no	no	no	no	no	211	yes	4.67
PDM-4	50	Male	White	Colon	Sigmoid colon	Stage IIIB	G2	yes	no	no	yes	no	no	102	no	5.14
PDM-95	61	Male	Black or African American	Colon	Sigmoid colon	Stage IIIB	GB	yes	no	no	yes	no	no	116	no	5.45
PDM-279	51	Male	Black	Colon	Cecum	Stage 0	GX	yes	yes	no	no	no	yes	173	no	6.9
PDM-2	68	Male	White	Colon	Sigmoid colon	Stage IVA	GB	yes	no	no	yes	no	no	129	yes	7.86
PDM-50	78	Male	White	Colon	Ascending colon	Stage IIIB	G3	no	no	no	no	no	no	0	no	8.53
PDM-9	63	Male	Asian	Colon	Liver	Stage IVA	G2	yes	no	no	yes	no	no	139	no	11.1
PDM-276	54	Male	Black or African American	Colon	Transverse colon	ungrouped	ungrouped	yes	no	yes	no	no	no	109	no	19.85
PDM-1	75	Male	White	Colon	Cecum	Stage I	G2	yes	no	yes	yes	no	no	191	no	27.9
PDM-185	71	Male	White	Colon	Cecum	Stage IIA	G2	yes	no	yes	no	no	no	154	no	40
PDM-94	67	Male	White	Colon	Sigmoid colon	ungrouped	G2	yes	no	no	yes	yes	no	168	no	40
PDM-7	75	Male	Black or African American	Colon	Sigmoid colon	Stage IIIA	G2	yes	no	yes	yes	no	no	156	no	40
PDM-257	53	Female	Unknown	Rectosigmoid junction	Rectosigmoid junction	Stage IIIB	G2	yes	no	yes	yes	yes	no	155	no	40
PDM-363	72	Male	Unknown	Colon	Cecum	ungrouped	GX	yes	yes	no	yes	yes	yes	2701	no	40
PDM-277	76	Female	White	Colon	Lymph node(s)	Stage IVA	G2	yes	no	yes	yes	no	no	209	no	40

PDM-359	64	Female	Unknown	Colon	Ascending colon	ungrouped	G2	yes	yes	no	no	no	yes	1502	no	40
PDM-103	51	Female	Black or African American	Colon	Liver	ungrouped	ungrouped	yes	no	yes	yes	no	no	171	yes	40

Supplemental Table 7 **[Related to Figure 5 and S7]:** A catalogue of all odds ratios and associated statistics for the 50-gene signature and CDX2 alone across various publicly available datasets.

50 gene signature	
Marisa-GSE39582 (n = 545; Colorectal cancers; cohort enriched for KRAS/BRAF mut) [Entire cohort]	
Odds ratio	1.546
95 % CI:	1.0826 to 2.2077
z statistic	2.397
Significance level	P = 0.0166
Hu-GSE87211 (n = 353; Colon cancers)	
Odds ratio	2.1917
95 % CI:	1.3313 to 3.6082
z statistic	3.085
Significance level	P = 0.0020
Gaedcke-GSE40492 (n = 219; Rectal cancers)	
Odds ratio	1.7234
95 % CI:	0.8983 to 3.3065
z statistic	1.637
Significance level	P = 0.1015
Jorissen-GSE13067, GSE14333; Smith-GSE17538; Medema-GSE33113; Thorsteinsson-GSE31595; Olschwang-GSE37892 (Pooled: n = 555; Colon cancers)	
Odds ratio	2.1306
95 % CI:	1.4365 to 3.1599
z statistic	3.761
Significance level	P = 0.0002
CDX2	
Marisa-GSE39582 (n = 545; Colorectal cancers; cohort enriched for KRAS/BRAF mut) [Entire cohort]	
Odds ratio	0.6751
95 % CI:	0.4424 to 1.0301
z statistic	1.822
Significance level	P = 0.0684
Hu-GSE87211 (n = 353; Colon cancers)	
Odds ratio	0.86
95 % CI:	0.5096 to 1.4512
z statistic	0.565
Significance level	P = 0.5721
Gaedcke-GSE40492 (n = 219; Rectal cancers)	
Odds ratio	1.0074
95 % CI:	0.4579 to 2.2163
z statistic	0.018
Significance level	P = 0.9854

Jorissen-GSE13067, GSE14333; Smith-GSE17538; Medema-GSE33113; Thorsteinsson-GSE31595; Olschwang-GSE37892 (Pooled: n = 555; Colon cancers)	
Odds ratio	0.5603
95 % CI:	0.3737 to 0.8399
z statistic	2.805
Significance level	P = 0.005
